# Development of an *in-vivo* active reversible butyrylcholinesterase inhibitor

**DOI:** 10.1038/srep39495

**Published:** 2016-12-21

**Authors:** Urban Košak, Boris Brus, Damijan Knez, Roman Šink, Simon Žakelj, Jurij Trontelj, Anja Pišlar, Jasna Šlenc, Martina Gobec, Marko Živin, Larisa Tratnjek, Martina Perše, Kinga Sałat, Adrian Podkowa, Barbara Filipek, Florian Nachon, Xavier Brazzolotto, Anna Więckowska, Barbara Malawska, Jure Stojan, Irena Mlinarič Raščan, Janko Kos, Nicolas Coquelle, Jacques-Philippe Colletier, Stanislav Gobec

**Affiliations:** 1 Faculty of Pharmacy, University of Ljubljana, Aškerčeva 7, 1000 Ljubljana, Slovenia; 2 Institute of Pathological Physiology, Faculty of Medicine, University of Ljubljana, Vrazov trg 2, 1000 Ljubljana, Slovenia; 3 Institute of Pathology, Faculty of Medicine, University of Ljubljana, Korytkova 2, 1000 Ljubljana, Slovenia; 4 Faculty of Pharmacy, Jagiellonian University, Medyczna 9 St., 30-688 Krakow, Poland; 5 Institut de Recherche Biomédicale des Armées, 91223 Brétigny sur Orge, France; 6 Institute of Biochemistry, Faculty of Medicine, University of Ljubljana, Vrazov trg 2, 1000 Ljubljana, Slovenia; 7 University Grenoble Alpes, IBS, F-38044 Grenoble, France; 8 CNRS, IBS, F-38044 Grenoble, France; 9 CEA, IBS, F-38044 Grenoble, France

## Abstract

Alzheimer’s disease (AD) is characterized by severe basal forebrain cholinergic deficit, which results in progressive and chronic deterioration of memory and cognitive functions. Similar to acetylcholinesterase, butyrylcholinesterase (BChE) contributes to the termination of cholinergic neurotransmission. Its enzymatic activity increases with the disease progression, thus classifying BChE as a viable therapeutic target in advanced AD. Potent, selective and reversible human BChE inhibitors were developed. The solved crystal structure of human BChE in complex with the most potent inhibitor reveals its binding mode and provides the molecular basis of its low nanomolar potency. Additionally, this compound is noncytotoxic and has neuroprotective properties. Furthermore, this inhibitor moderately crosses the blood-brain barrier and improves memory, cognitive functions and learning abilities of mice in a model of the cholinergic deficit that characterizes AD, without producing acute cholinergic adverse effects. Our study provides an advanced lead compound for developing drugs for alleviating symptoms caused by cholinergic hypofunction in advanced AD.

Alzheimer’s disease (AD) is a complex neurodegenerative disorder that is characterized by progressive and chronic deterioration of memory and other cognitive functions. This disease leads to death within 3 years to 9 years after diagnosis, and as the leading cause of dementia, it affects 44 million people worldwide^1^.

Even though the etiology of AD is very complex, several conditions are known to participate in the associated neurodegeneration. Accumulation of amyloid β peptide (Aβ) deposits^2^,^3^, abnormal modification and accumulation of the protein tau^4^ accompanied with oxidative stress in the brain lead to synaptic dysfunction and neurodegeneration^1^. This most severely affects the cholinergic system^5^ and results in a decrease in the levels of the neurotransmitter acetylcholine (ACh)^6^, which in turn produces memory and cognitive deficits^7^, characteristic for patients with AD. Cholinergic neurotransmission in the brain is terminated by the hydrolysis of ACh, which is catalyzed by two cholinesterases (ChEs): acetylcholinesterase (AChE) and butyrylcholinesterase (BChE)^8^. In the brain of healthy adults, AChE accounts for 80% of the ChE activity, with BChE accounting for the remainder^9^.

As long as AD cannot be prevented, symptomatic treatment is essential. Three out of the four approved drugs for treatment of patients with AD are ChE inhibitors: the selective reversible AChE inhibitors donepezil^10^ and galantamine^11^, and the pseudo-irreversible dual ChE inhibitor rivastigmine^12^ (Fig. 1a). These drugs exploit ChE inhibition to alleviate the symptoms of AD, or to temporarily slow down its progression, by restoring the cholinergic activity in the brain. However, inhibition of AChE in the peripheral nervous system and the parasympathetic autonomic nervous system represents the basis for the adverse side effects (e.g., nausea, vomiting, diarrhea, tremors)^13^, and also limits the doses^14^ of these drugs that can be administered. Additionally, the clinical efficacy of this drug class is mostly limited to mild and moderate stages of AD^15^,^16^.

The assumed co-regulatory function of BChE in termination of cholinergic neurotransmission in the healthy brain changes in brains of patients with progressive AD. In specific brain regions of these patients enzymatic activity of BChE is increased^7^. Increased BChE activity^7^ in addition to reduced expression of neuronal AChE in advanced AD^9^, findings that AChE knockout mice are viable and develop a primitive cholinergic neuronal network with BChE acting as a surrogate for AChE and taking over the hydrolysis of ACh in the brain^17^, along with the fact that BChE knockout mice show no physiological disadvantages^18^, have led to the hypothesis that BChE takes over the ChE activity in advanced AD, and thus needs to be inhibited to restore the brain levels of ACh^17^. In line with this hypothesis, in aged rats, selective inhibition of BChE with cymserine analogs raises ACh levels in the brain and improves cognitive performance^19^ without any adverse parasympathetic side effects^13^. Altogether, these data suggest that BChE is a potential therapeutic target for restoring ACh levels in the brain and thus improving cognitive impairment, while also minimizing adverse effects in patients with progressive AD.

Although a number of structurally diverse selective reversible and pseudo-irreversible BChE inhibitors have been reported^19^,^20^,^21^,^22^,^23^,^24^,^25^, cymserine analogs are the only BChE inhibitors with reported *in-vivo* activity^19^, and one of them has successfully advanced to Phase I clinical trials: bisnorcymserine (Fig. 1a). Cymserine analogs are pseudo-irreversible carbamate inhibitors, the mechanism of action of which involves a rapid initial covalent reaction between their carbamate carbonyl group and the catalytic serine in the active site of ChEs (carbamoylation), followed by a slower regeneration (decarbamylation) of the active enzyme^26^. Pseudo-irreversible inhibitors are thus reversible covalent inhibitors, and since they derive their activity by forming a covalent bond with their target, concerns remain over their potential nonspecific or off-target reactivity-based toxicities^27^,^28^. *In-vivo* active selective reversible BChE inhibitors are thus highly desirable not only as potential clinical candidates for improving memory and cognitive deficits in patients with progressive AD, but also as molecular tools for studying the potential of BChE as a therapeutic target. The structures of human BChE (huBChE) and its active site gorge are shown in Fig. 1b,c.

Recently, a novel piperidine-3-ylmethanamine–based selective huBChE inhibitor **1** was reported (Fig. 1a) that shows reversible, slow–tight binding inhibition of huBChE with low nanomolar IC_50_^29^. This inhibitor was used here as the starting point for the design, synthesis, and biochemical evaluation of a comprehensive series of sulfonamides. The most potent of these, compound **2**, shows selective reversible nanomolar huBChE inhibition with a new binding mode, as revealed by the crystal structure of its complex with huBChE. Furthermore, compound **2** improves memory, cognitive functions, and learning abilities of mice in a model that mimics the symptoms of AD caused by cholinergic hypofunction, without producing acute cholinergic adverse effects.

## Results

### Design and synthesis of inhibitors

Our quest for novel sulfonamide BChE inhibitors began before the crystal structure of hit compound **1** in complex with huBChE was solved. Thus, the initial design efforts were based solely on the chemical structure of hit compound **1**, which made it imperative that this design and synthesis yielded compounds with drug-like properties^30^ and good synthetic tractability.

**Figure 2:**
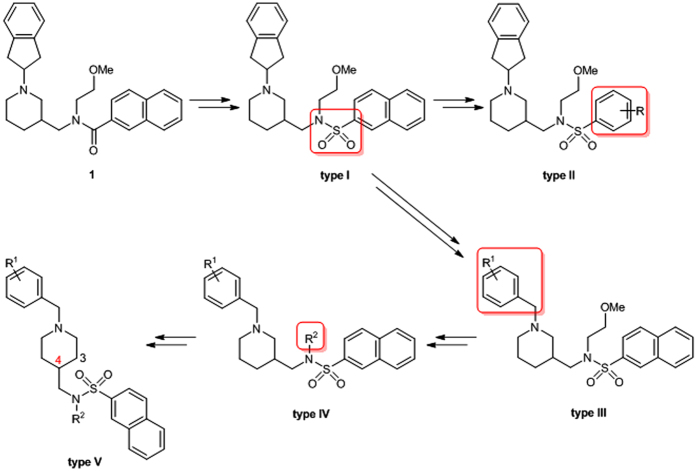
Design of sulfonamide analogs of hit compound 1. The modifications introduced during each design step are indicated in red.

The bioisosteric replacement of a carboxamide with a sulfonamide group is a common design strategy in medicinal chemistry that has been used to improve biological activity, bioavailability, and metabolic stability of drug candidates^31^. Accordingly, the **type I** inhibitor was designed by replacing the carboxamide group of compound **1** with a sulfonamide group. Additional types of inhibitors were designed to study the effects of these structural modifications on the inhibitory potencies against huBChE. The naphthalene moiety was truncated and replaced with various substituted phenyl rings (**type II** inhibitors). Analogously, the 2,3-dihydro-1*H*-inden moiety was replaced with various benzyl rings (**type III** inhibitors), and the importance of the *N*-alkyl chain on the sulfonamide nitrogen of these inhibitors was studied by its removal or replacement with linear alkyl substituents (**type IV** inhibitors). Finally, 1,4-disubstituted piperidine derivatives (**type V** inhibitors) were designed to determine the effects of a piperidine ring disubstitution pattern (Fig. 2). In conjunction with this, a synthesis route was planned that provided the simple and rapid generation of new inhibitors. Details regarding the synthesis of all of the 41 inhibitors are given in Supplementary Figures 1 to 5.

### *In-vitro* ChE inhibition and structure–activity relationships

The inhibitory potencies against huBChE and murine AChE (mAChE) were determined for all of these synthesized compounds using the method of Ellman^32^. mAChE was chosen as the surrogate for huAChE on the basis that the distribution of the amino-acid residues along the active site gorge of these two enzymes is strictly conserved^33^. Table 1 gives the inhibitory potencies (i.e., IC_50_ values, % residual activities [RAs]) and structures of the most potent of these **type I**–**V** inhibitors (**2**–**6**), and for a comparison, those of initial hit compound **1**. The complete list of all 41 sulfonamides is given in Supplementary Table 1. Briefly, these IC_50_ values against huBChE ranged from 4.9 nM to 8270 nM, and all of these compounds showed significant selectivity towards huBChE over mAChE. Bioisosteric replacement of carboxamide in hit compound **1** with sulfonamide in the **type I** inhibitor (compound **3**) slightly reduced the inhibitory potency against huBChE. When the naphthalene ring of the **type I** inhibitor compound **3** was replaced with substituted phenyl rings (e.g., **type II** inhibitors), the inhibitory potencies against huBChE dropped significantly. The naphthalene ring was therefore retained, and the left-hand side of the **type I** inhibitor structure illustrated in Table 1 was modified further. The replacement of 2,3-dihidro-1*H*-inden ring with benzyl groups yielded **type III** inhibitors, among which compound **2** was the most potent inhibitor of the series, with an IC_50_ of 4.9 nM against huBChE. The **type IV** inhibitors revealed that variations in the *N*-alkyl substituent on the sulfonamide nitrogen were permitted for maintained inhibitory potency against huBChE, although its removal was not (Table 1 and Supplementary Table 1). Additionally, introduction of substituents to the benzyl and naphthalene rings of **type III** and **type IV** inhibitors were not tolerated for inhibitory activity against huBChE (Supplementary Table 1). **Type V** inhibitors showed that 1,3 disubstituted piperidines were more potent huBChE inhibitors than their 1,4 disubstituted counterparts (Table 1 and Supplementary Table 1).

### Chiral HPLC resolution and kinetic evaluation of compound 2

**Figure 3:**
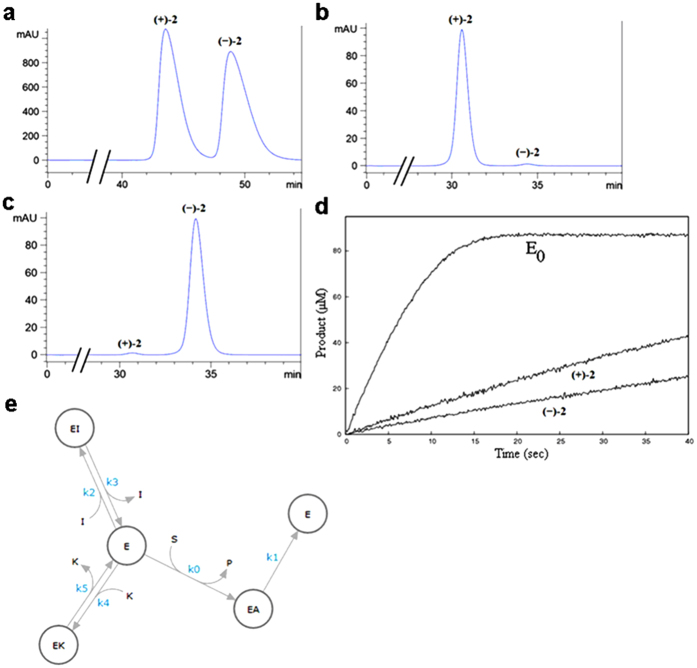
Chiral HPLC resolution of racemic compound (±)-2 into pure enantiomers, and their kinetic evaluation. (**a**) Semi-preparative reversed-phase HPLC of racemic compound (**±**)**-2** (Supplementary Methods [method A; 254 nm]). (**b**) Analytical reversed-phase chiral HPLC of (**+**)**-2** (Supplementary Methods [method B; 254 nm]). (**c**) Analytical reversed-phase chiral HPLC of **(−)-2** (Supplementary Methods [method B; 254 nm]). (**d**) Progress curves for hydrolysis of 44 μM butyrylthiocholine iodide by huBChE in the absence (E_0_) and presence of 50 nM of each pure enantiomer of compound **2** (as indicated). Data were obtained using a stopped-flow apparatus. (**e**) A competitive single-step inhibition mechanism with fast association (high k2, k4) for each pure enantiomer fully reproduced the progress curves obtained. S, substrate; P, product; E, enzyme; EA, acylated enzyme; I, **(−)**-2; K, (**+**)-2; k0–k5, kinetic constants.

Compound **2** was resolved into its pure enantiomers using semi-preparative reversed-phase chiral HPLC (Fig. 3a and Supplementary Methods). The optical purities of the enantiomers were determined by measurement of the optical rotation (Supplementary Methods) and by determination of the enantiomeric excess (e.e.) using analytical reversed-phased chiral HPLC (Fig. 3b,c, and Supplementary Methods). Both of these enantiomers were obtained with e.e. 97%. The inhibitory potencies of these separated enantiomers were evaluated, whereby eutomer **(−)-2** had an IC_50_ of 4.5 nM and was a 3.4-fold more potent huBChE inhibitor than the distomer (**+**)**-2** (IC_50_ = 15.3 nM). Given the affinity of compound **(−)-2** for its target enzyme huBChE, standard methods for *K*_*i*_ determination (e.g., Lineweaver-Burk plots) or approximation (e.g., Cheng-Prusoff equations) are inappropriate, as i) the target enzyme huBChE does not obey the typical Michaelis-Menten kinetics^34^ and ii) the concentration of the inhibitor is approaching the enzyme concentration (50 and 8.2 nM, respectively) in the assay system. This situation is referred to as tight binding inhibition. Given these facts, in order to obtain the true affinity of the pure enantiomers of compound **2**, the enzyme kinetics experiments were carried out in a stopped-flow apparatus, according to Copeland’s guidelines for evaluation of tight-binding inhibitors^35^. The full progress curves of product formation were followed in the absence and presence of each pure enantiomer of **2** (Fig. 3d). Of note, a high enzyme concentration was used in order to investigate the time-dependent inhibition and to reach the plateau of the reaction, which allowed precise quantification of the substrate concentration. No curvature in the initial phase of the inhibited enzyme reaction was observed, indicating that the compound **2** does not display slow-binding inhibition. The curves were analyzed simultaneously using the ENZO application^36^. Several inhibition mechanisms were tested, and the single-step competitive mechanism allowed full reproduction of the progress curves (Fig. 3e). The inhibition constants of 1.29 nM and 2.01 nM were determined for the enantiomers **(−)-2** and (**+**)**-2**, respectively. The determined rates and the calculated dissociation constants for each of these enantiomers, along with the K_m_ and k_cat_ values for butyrylthiocholine iodide, are given in Supplementary Table 2. The low degree of stereoselective inhibition provided an advantage for compound **2**, as it enabled the use of the readily available synthetic racemic mixture in further *in-vitro* and *in-vivo* studies.

### Crystal structures of huBChE in complex with compounds 2, 7, and 8

**Figure 4:**
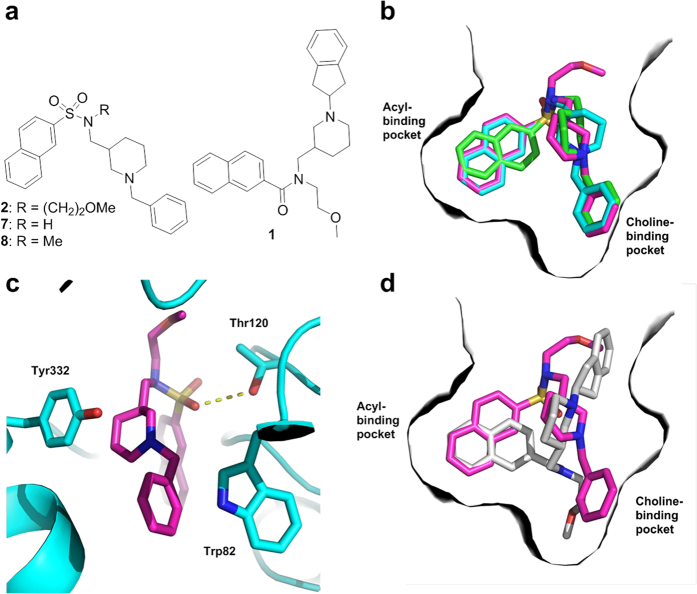
Crystal structures of huBChE in complex with compounds 2, 7, and 8. (**a**) Two-dimensional structures of the co-crystalized sulfonamides **2**, **7**, and **8** and hit compound **1**. (**b**) Alignment of crystal structures of compounds **2** (purple), **7** (green), and **8** (cyan) in their complexes with huBChE (gray surface). These inhibitors fully occupy the acyl-binding and choline-binding pockets with their naphthalene and benzyl moieties, respectively. (**c**) The polar interactions of compound **2** (purple sticks) with the amino-acid residues of the huBChE active site (blue) contribute significantly to the binding affinity. The observed H-bond between Thr120 and the sulfonamide moiety is shown as yellow dashes (distance, 3.1 Å). Compound **2** forms cation-π and π-π aromatic interactions with Tyr332 and Trp82, respectively. (**d**) Alignment of crystal structures of compound **2** (purple) and hit compound **1** (white) in their complexes with huBChE (white surface). PDB codes: 5DYW (compound **2**), 5DYY (compound **7**), and 5DYT (compound **8**).

The crystallographic structures of huBChE in complex with compounds **2** [-(CH_2_)_2_-OMe], **7** (-H), and **8** (-Me) were solved. These compounds differ only in terms of the presence and nature of their *N*-alkyl group (Fig. 4a), and hence share a similar binding modes for huBChE (Fig. 4b and Supplementary Figure 6). Specifically, the positively charged nitrogen of their piperidine moiety engages in cation-π interaction with the Tyr332 side chain, while the attached benzyl ring fits into a groove contributed by Tyr82, Tyr332, Trp430, and Tyr440 side chains, in the choline-binding pocket. The naphthalene moiety is T-stacked (i.e., π-π interaction) to Trp231 in the acyl-binding pocket, while one of the sulfonamide oxygens forms a H-bond to the hydroxyl oxygen of Thr120 (Fig. 4c and Supplementary Figure 1). The -(CH_2_)_2_-OMe (compound **2**) or methyl (compound **8**) side chains point out of the gorge (Fig. 4b). This binding mode was not expected, given that the -(CH_2_)_2_-OMe side chain of hit compound **1** (Fig. 4a), which served as the scaffold for the design of these sulfonamide inhibitors, is instead oriented toward the choline-binding pocket (Fig. 4d)^28^. This phenomenon can be explained by the different hydrogen-bonding and geometrical preferences of the carboxamide (compound **1**) and sulfonamide (compound **2**) groups^37^. Furthermore, a H-bond is observed between the ether oxygen of compound **2** and a structural water, which is itself H-bonded to Asn68. The position of this oxygen appears to be important for the affinity of compound **2**, as the additional methylene group in the *N*-alkyl chain [(CH_2_)_3_OMe] of compound **5** resulted in a 3-fold higher IC_50_ against huBChE than for compound **2** (Table 1). As observed already in the complex structure of huBChE with compound **1**^28^, no conformational change is undergone by the enzyme upon binding of compounds **2**, **7**, and **8**. This observation suggests that the active site of huBChE is fairly rigid upon binding and that tailor-made compounds are required to achieve potent huBChE inhibition. The larger binding surface and the greater surface complementarity (Supplementary Table 3) indeed correlate with higher affinity (Supplementary Table 1). The 2m*Fo*–D*Fc* electron density map of compound **2** bound in the active site of huBChE is shown in Supplementary Figure 7. As both the *R* and *S* enantiomers of these sulfonamide inhibitors provided a fit to the electron density maps, all of the inhibitors were assigned to the *R*-enantiomers, on the basis of a possible cation-π interaction between the positively charged nitrogen of the piperidine moiety and the side chain of Tyr332, which is not possible for *S*-enantiomers.

### Inhibition of BChE in rat brain slices

**Figure 5:**
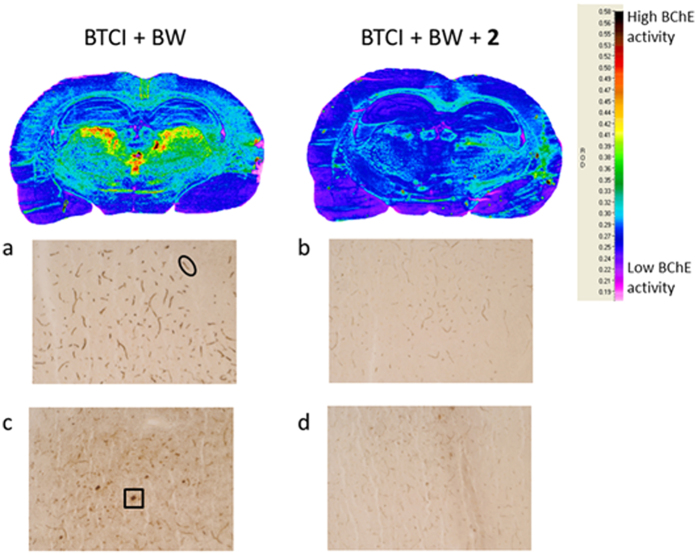
Inhibition of BChE in rat brain slices. Representative coronal section from a rat brain in the region of the thalamus processed for BChE histochemical staining using 4 mM butyrylthiocholine iodide (BTCI), in the absence (left) and presence (right) of 300 μM compound **2**. Here, 10 mM BW-284C51 (BW) was added to completely block AChE activity. Vascular structures in the cortex (black oval highlight) and BChE-rich neurons in the laterodorsal thalamic nucleus (black box highlight) in the absence (**a**,**c**) (respectively) and presence (**b**,**d**) (respectively) of compound **2**. Magnification, 10×. The relative optical density (ROD) scores for the staining intensity for BChE activity are shown on the right.

To investigate whether compound **2** can efficiently inhibit BChE in an environment that is more similar to *in-vivo* conditions, inhibition of BChE in rat brain slices was performed. To detect the regions with the highest BChE activities in the rat brain, these were mapped and processed using a modified Koelle-Karnovsky histochemical method^38^. The dark brown/black granular BChE reaction product was mainly associated with glial cells, and vascular and neuronal structures. Most of the BChE-positive neurons were in the thalamus, neocortex, and amygdala, while very few were detected in the striatum and hippocampus, which is in agreement with previously published studies^39^,^40^. The highest BChE activity was detected in the laterodorsal thalamic nucleus, and the sections containing this region were further used in the inhibition experiments. There was significant reduction in the dark brown/black BChE reaction product that was formed when these sections were incubated with 300 μM compound **2** (Fig. 5).

### Cytotoxicity and neuroprotective effects of compound 2

Prior to any *in-vivo* investigations, the cytotoxicity profiles of compound **2** on the human liver cancer HepG2 (Supplementary Figure 8a) and neuroblastoma SH-SY5Y (Supplementary Figure 8b) cell lines were determined. The LD_50_ value for compound **2** with HepG2 cells was 58.67 μM, and with SH-SY5Y cells, 31.40 μM. These LD_50_ values were almost 12,000-fold and >6,400-fold greater, respectively, than the concentrations needed to achieve 50% *in-vitro* inhibition of huBChE.

Neurotoxicity induced by Aβ species (mainly Aβ_(1–42)_) is a contributor to the pathogenesis of neurodegeneration in AD^41^. To determine whether compound **2** can protect neuronal cells from toxic Aβ-species, comparisons were made of the neuronal death induced by Aβ_(1–42)_ in the absence and presence of various concentrations of compound **2**. As shown in Supplementary Figure 8c, treatment of SH-SY5Y cells with 5 μM Aβ_(1–42)_ caused significant toxicity, whereas a clear dose-response neuroprotective effect was observed when the cells were exposed to Aβ_(1–42)_ in the presence of compound **2**. Indeed, at 10 μM, this BChE inhibitor reversed Aβ_(1–42)_-induced cell death. The neuroprotective effects of compound **2** are independent of Aβ_(1–42)_ aggregation as 10 μM compound **2** did not inhibit Aβ_(1–42)_ aggregation (Supplementary Table 4).

### *In-vitro* pharmacokinetics of compound 2

Since permeability values (*P*_app_) of compounds in Caco-2 cells (heterogeneous human epithelial colorectal adenocarcinoma cells) correlate well with *in-vivo* permeation^42^, simple *in-vitro* bidirectional permeability measurements in these cells are used to predict the absorption in humans^43^. Caco-2 cells are also used to investigate a compounds transport mechanisms^43^, as they express membrane transport proteins like P-glycoprotein or breast cancer resistance protein (BCRP)^44^, which actively efflux drugs^42^. The *P*_app_ of compound **2** was determined in both directions, in eliminatory [basolateral-to-apical (B–A)] and absorptive [apical-to-basolateral (A–B)], and the efflux ratio [*P*_app(B–A)_/*P*_app(A–B)_] was calculated. The *P*_app(B–A)_ of compound **2** (29.1 ± 2.9) × 10^−6^ cm s^−1^, was “high” according to the biopharmaceutical classification system of drug permeability since it is comparable to those of antipyrine, naproxen, propranolol and theophylline previously measured in the same experimental setting in the same laboratory^45^. In the opposite direction the *P*_app(A–B)_ was (30.0 ± 10.7) × 10^−6^ cm s^−1^ which results in an efflux ratio (*P*_B–A_/*P*_A–B_) of 0.97 and demonstrates that compound **2** is not subject to any significant active efflux mechanisms^46^.

10 mM compound **2** was incubated with human blood plasma to determine its plasma protein binding. Equilibrium dialysis showed that compound **2** is 96% protein bound (Supplementary Table 5), which is higher than the threshold of 90%, above which a compound is classified as highly protein bound^47^.

The *in-vitro* metabolic stability was studied by performing incubations of compound **2** in the presence of human blood plasma to determine the plasma half-life and with human cryoperserved hepatocytes (0.7 million viable cells/mL) to determine the intrinsic hepatic metabolic clearance. The plasma metabolic stability and hepatic clearance were estimated by the substrate depletion method. Qualitative aspect of metabolic degradation was performed by metabolite identification using LC-high resolution mass spectrometry (HRMS). We determined that the *in-vitro* half-life of compound **2** in human plasma was >120 min (Supplementary Table 6), while its hepatic metabolism assessed by cryoperserved hepatocytes was more pronounced (t_1/2_ = 54 min) (Supplementary Table 7). This half-life of compound **2** is comparable to other high extraction ratio reference compounds cleared by CYP enzymes that were used in the metabolic stability experiment as positive controls (Supplementary Table 7). The marked hepatocyte clearance (18.2 μL/min/million cells) (Supplementary Table 7) of compound **2** is in agreement with the three possible metabolites identified with LC-HRMS: *N*-debenzylated, demethylated, and a demethylated + glucoronide products (Supplementary Figure 9 and Supplementary Table 8).

### *In-vivo* blood plasma–brain distribution of compound 2

Partition into the brain is essential for compounds designed to be active in the central nervous system, and the most reliable evaluation of this crossing of the blood–brain barrier (BBB) by any molecule comes from direct *in-vivo* measurements of the distribution between the blood plasma and the central nervous system^42^. The distribution of compound **2** between the blood plasma and the brain was therefore measured in 3-month-old female Wistar Han rats, using liquid chromatography combined with tandem mass spectrometry (LC-MS/MS) for the analysis. One hour after these rats had an intraperitoneal (IP) injection of 10 mg kg^−1^ compound **2**, or 2 mg kg^−1^ donepezil (positive control), or vehicle (negative control), they were euthanized, and blood samples were collected and the brain tissue was isolated. The methods developed for the preparation of the blood plasma and the brain tissue included homogenization, centrifugation, and solid-phase extraction for preparation of the samples for LC-MS/MS quantification (Methods, Supplementary Methods).

Using a validated UHPLC-MS/MS method (Supplementary Methods), the mean ± SEM concentration of compound **2** in the brain tissue was determined as 78 ± 7 μg kg^−1^, while that in the blood plasma was 168 ± 10 μg L^−1^. The brain-to-plasma ratio of compound **2** was thus 0.44. This procedure was verified using the donepezil positive control, which provided a brain-to-plasma ratio of 6.3, which is in agreement with previous reports^48^. This lower BBB permeability of compound **2** did not prevent us from carrying out further investigates into this compound. High BBB permeability is essential for acute treatments, e.g., analgesics for acute pain, and it is of lesser importance for chronic administration schedules, e.g., ChE inhibitors for AD, where even compounds with modest permeability can produce significant pharmacological effects^42^.

Among the major reason for limited brain exposure of compounds designed to be active in the central nervous system is their low passive permeability combined with active efflux by membrane transport proteins like P-glycoprotein or breast cancer resistance protein (BCRP) and high molecular weight^42^. The results of permeability measurments with Caco-2 cells show that neither low passive permeability nor active efflux are likely not the reason for the relatively modest brain-to-plasma ratio of compound **2**. This could be due to its molecular weight (453 Da), which is considered borderline high for favorable distribution into the brain^42^.

### *In-vivo* activity of compound 2

#### Passive avoidance task

**Figure 6:**
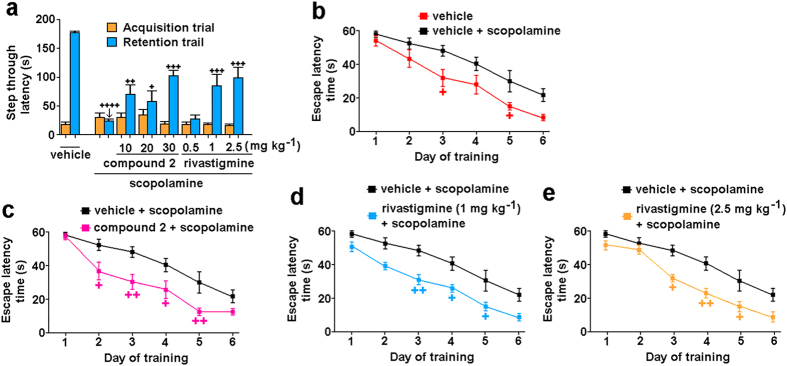
Effects of compound **2** and rivastigmine on scopolamine-induced memory impairment in the passive avoidance (**a**) and the Morris water maze tasks (**b**–**e**). (**a**) Data are mean step-through latencies ± SEM (n = 9–10 mice per group). Significance: ^+^p < 0.05; ^++^p < 0.01; ^+++^p < 0.001; ^++++^p < 0.001, *versus* scopolamine-treated control (in the retention phase). (**b**–**e**) Learning curves showing acquisition phase for vehicle-treated mice and scopolamine-treated control mice (**b**), scopolamine-treated control mice and scopolamine-induced memory-impaired mice treated with 30 mg kg^−1^ compound **2** (**c**), or 1 mg kg^−1^ (**d**) or 2.5 mg kg^−1^ (**e**) rivastigmine. Data are mean escape latency ± SEM from four daily trials. Significance *versus* scopolamine-treated control: ^+^p < 0.05; ^++^p < 0.01.

The first behavioral test performed was the passive avoidance (PA) task, which allows the evaluation of the effects of a drug on fear-motivated contextual memory. This is one of the most frequently used behavioral methods to measure the effects of drug candidates on cognitive abilities in rodents^49^. In the passive avoidance task, better memory performance is determined according to a longer step-through latency in the retention trial (i.e., the testing phase) than in the acquisition trial (i.e., the conditioning phase). Comparison of the retention trial step-through latencies in scopolamine-treated and vehicle-treated mice (23, 178 s, respectively; Fig. 6a) demonstrated the amnesic effect of scopolamine on learning and memory. Administration of compound **2** before scopolamine led to significant prolongation of the step-through latencies at each dose tested (10, 20, 30 mg kg^−1^, for latencies of 70, 58, 102 s, respectively). Administration of 1 mg kg^−1^ and 2.5 mg kg^−1^ rivastigmine also prolonged the step-through latency (85, 99 s, respectively), while 0.5 mg kg^−1^ rivastigmine was not active (Fig. 6a). As compound **2** was tested *in-vivo* for the first time, and as no effective concentration ranges had been established before, the two further behavioral tests were carried out with 30 mg kg^−1^ compound **2**.

#### Morris water maze task

**Figure 7:**
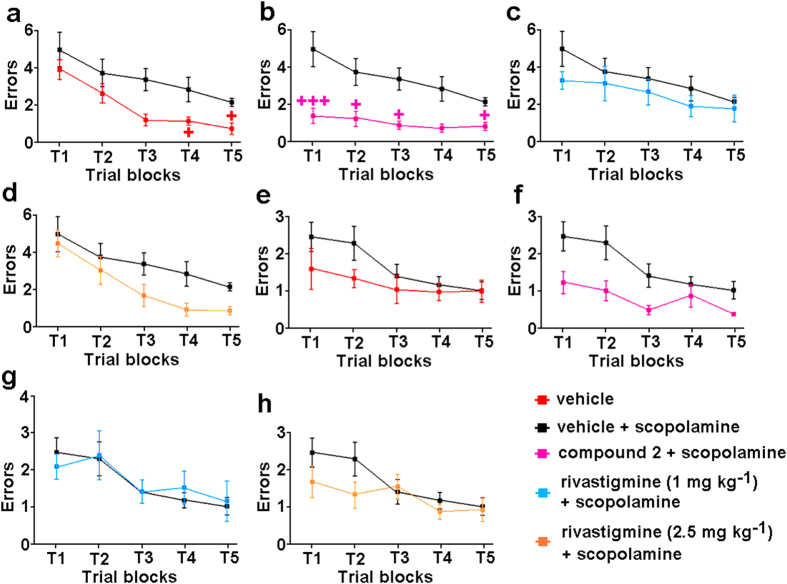
Effects of compound 2 and rivastigmine on scopolamine-induced memory impairment in the two-day radial arm water maze task. Spatial learning deficits expressed as mean number of errors ± SEM in 15 trials in five blocks (T1–T5) of three trials on day 1 (**a**–**d**) and day 2 (**e**–**h**) of the RAWM task. Vehicle-treated mice and scopolamine-treated control mice (**a**,**f**), scopolamine-treated control mice and scopolamine-induced memory-impaired mice treated with 30 mg kg^−1^ compound **2** (**b**,**f**), or 1 mg kg^−1^ (**c**,**g**) or 2.5 mg kg^−1^ (**d**,**h**) rivastigmine. Significance *versus* scopolamine-treated control mice: ^+^p < 0.05; ^+++^p < 0.001.

The Morris water maze (MWM) task is a hippocampus-dependent spatial learning and memory task that provides insight into long-term memory through decreases in escape latency time, and into cognition through the measure of the overall distance travelled to reach the platform^50^,^51^. In this task the time taken to reach the hidden platform (i.e., the escape latency time), the distance travelled, and the mean speed of swimming are recorded for each experimental group. For all of the groups of mice tested, the latency times to reach the platform tended to decrease along with the training, according to characteristic learning curves (Fig. 7b–e). The amnesic effect of scopolamine was seen from day 3, with a statistically significant increase in the distance travelled to reach the platform (vehicle treated, 3.77 m; scopolamine treated, 8.71 m), which was more pronounced on day 5 (1.94, 5.40 m, respectively). Mice treated with scopolamine and compound **2** travelled 5.38 m on day 3, and 1.69 m on day 5, which demonstrated the procognitive effect of compound **2**. In comparison, the mice treated with scopolamine and 2.5 mg kg^−1^ rivastigmine travelled 3.64 m and 1.74 m on days 3 and 5, respectively. This effect was also observed at the lower treatment of 1 mg kg^−1^ rivastigmine (day 3, 4.62 m; day 5, 1.58 m). The mean speed of the swimming was also determined to exclude any underlying sensorimotor deficits in the animals treated with compound **2** and rivastigmine^52^. Compound **2** did not reduce the mean speed of swimming (vehicle, 0.17 m s^−1^; scopolamine, 0.15 m s^−1^; scopolamine + compound **2**, 0.16 m s^−1^), although rivastigmine decreased it (1 mg kg^−1^, 0.15 m s^−1^; 2.5 mg kg^−1^, 0.12 m s^−1^).

#### Two-day radial arm water maze task

The radial arm water maze (RAWM) learning and memory task is a common method used to study memory deficits in mice, and it combines the spatial complexity of the dry radial arm with the rapid learning and strong motivation observed in the MWM^53^,^54^. In this task incorrect arm entries (i.e., errors) are counted. The vehicle-treated mice had a mean of 4 errors during the first trial block, but <1 error by the end of the first day (Fig. 7a). In comparison, by the end of the five blocks of trials, the scopolamine-treated mice had a mean of nearly 3 errors (Fig. 7a). Mice treated with scopolamine and either compound **2** (30 mg kg^−1^) (Fig. 7b) or rivastigmine (2.5 mg kg^−1^) (Fig. 7d) all finished the acquisition phase making <1 error. Of note, the dose of 1 mg kg^−1^ rivastigmine was not effective in this task (Fig. 7c,g). At the end of day two, all of the experimental groups completed the RAWM task with a mean performance of 1 error, with the exception of the mice treated with scopolamine and compound **2**, which showed better performance than the other groups, by making <1 error in the last trial block (Fig. 7e–h).

#### *In-vivo* safety profile of compound 2

To determine the *in-vivo* safety profile of compound **2** and properly interpret the results obtained in all three behavioural tasks, the impact of this inhibitor on motor coordination, sensorimotor functions and locomotor activity was determined. The effects of 30 mg kg^−1^ and 100 mg kg^−1^ compound **2** on motor coordination were tested with the rotarod test (Supplementary Methods). Here, compound **2** had no impact on the motor coordination of the mice, as it did not induce motor deficits, even at 100 mg kg^−1^ (Supplementary Table 9). Also, after administration of compound **2**, there were no acute cholinergic adverse effects in the mice, even at 100 mg kg^−1^ compound **2**. Any underlying sensorimotor deficits in the animals treated with compound **2** have also been excluded, as it did not reduce the mean speed of swimming in the MWM task (see Chapter “***Morris water maze task***”). Therefore, the observed transient reduction in the locomotor activity of mice (Supplementary Figure 9) did not appear to have any relevant impact on the data obtained in the learning and memory tests.

## Discussion

Using the previously reported huBChE inhibitor as a starting point, a comprehensive series of 41 new sulfonamide analogs were designed and synthesized. Five types of inhibitors were developed that showed nanomolar to micromolar inhibition of huBChE. See Supplementary Discussion for details regarding the structure–activity relationships of the complete series of inhibitors and the discussion regarding other sulfonamide ChE inhibitors. The solved crystal structures of complexes with three inhibitors revealed their binding modes and represent an excellent basis for their further structure-based optimization. A thorough biological evaluation of our most potent inhibitor **2** revealed that this compound inhibits BChE activity on rat brain slices, is not cytotoxic and protects neuronal cells from toxic Aβ-species. *In-vitro* Caco-2 cell permeability of compound **2** can be classified as “good” according to the biopharmaceutical classification system of drug permeability. This compound is also highly plasma protein bound, has a half-life in human plasma longer than 2 hours, and its metabolic stability in cryopreserved hepatocytes is comparable to flurazepam and naloxone. Furthermore, compound **2** permeates into the brain of rats and we assume that its relatively modest brain-to-plasma ratio is actually quite good considering its molecular weight.

Since test animals with an increased brain BChE/AChE enzymatic activity ratio are currently not available, animals with an unaltered brain BChE/AChE enzymatic activity ratio (CD-1 Krf and C57BL/6 J mice) were used. Procognitive properties of compound **2**, which targets the cholinergic system (BChE), were evaluated in an *in-vivo* model relevant to its mode of action. Scopolamine-induced amnesia was chosen since it is a model related to the cholinergic deficit that characterizes AD and is used in AD research for preclinical evaluation of cholinomimetics (compounds having an action similar to that of ACh)^55^ e.g., ChE inhibitors^55^,^56^,^57^,^58^,^59^. This model is useful for the evaluation of cognition-enhancing properties of ChE inhibitors without the risk of these drugs counteracting with the mechanism of the model^56^,^57^,^58^,^59^. Scopolamine, a non-selective muscarinic ACh receptor antagonist is administrated to test subjects to induce blockade of muscarinic ACh receptors in the central nervous system^60^,^61^ and produce cholinergic hypofunction at the pathophysiological level, and memory and cognitive deficits at the behavioural level^55^. Scopolamine thus produces memory and cognitive deficits comparable to those caused by the cholinergic hypofunction in AD^60^,^61^. Procognitive effects of ChE inhibitors in scopolamine-induced amnesia are the result of increased brain levels of ACh. ChE inhibitors interfere with the breakdown of ACh and cause its accumulation in the synapse, which then displaces scopolamine from muscarinic ACh receptors, and thus ameliorates the scopolamine-induced memory and cognitive deficits^60^,^62^.

The dose of scopolamine used to model cholinergic deficit varies among studies, with a range from 0.2 mg kg^−1^ to 2 mg kg^−1^, depending on the behavioral assay or drug considered. In the present study, 1 mg kg^−1^ scopolamine was injected IP before the acquisition trial of each test. This resulted in clear learning impairments in the negative controls used for the three behavioral tests performed. The anti-AD drug rivastigmine, which served as the positive control in these *in-vivo* assays, was used at concentrations comparable to those used in the clinic^63^. This selection was justified by the literature data that indicate that treatments with >2.5 mg kg^−1^ rivastigmine provide less ameliorating effects on scopolamine-induced cognitive deficits than those <2.5 mg kg^−1^ 63,64 while also producing significant adverse effects, including hypersalivation, intestinal hyperperistalsis, and muscle cramps (i.e., tremor and retraction of the hindlimbs)^63^.

Three behavioral tests were performed to determine whether compound **2** can attenuate the effects of scopolamine by increasing brain levels of ACh through selective BChE inhibition: the PA task, the MWM, and the two-day RAWM tasks. These behavioral tests and their various modifications have been used recently to model human cognition^65^. Details regarding these tests and their interpretation are given in the Methods and Supplementary Methods. The results of all three behavioral tasks performed *in-vivo* show that selective reversible BChE inhibition with compound **2** improved the memory, cognitive functions, and learning abilities of scopolamine-treated mice with an unaltered brain BChE/AChE enzymatic activity ratio. Compound **2** would thus be even more effective in alleviating symptoms caused by cholinergic hypofunction in advanced AD, in which brain BChE enzymatic activity is increased. The results also show that despite its relatively modest brain-to-plasma ratio, enough compound **2** partitions into the brain to ameliorate the induced memory and cognitive deficits.

As inhibition of ChEs in the basal ganglia, peripheral nervous system, and parasympathetic autonomic nervous system results in an excess of ACh, which is the basis for the adverse side effects of approved drugs for the treatment of patients with AD^13^, it was imperative to determine the *in-vivo* safety profile of compound **2**. We found no effects of compound **2** on motor coordination when tested with the rotarod test, no sensorimotor deficits in MWM task, and no acute cholinergic adverse effects in the mice, even at 100 mg kg^−1^. The combination of these properties makes compound **2** an advanced lead compound for developing drugs for alleviating symptoms caused by cholinergic hypofunction in advanced AD, in which brain BChE enzymatic activity is increased. Additionally, compound **2** is a powerful molecular tool to study BChE biology and to validate its potential as a therapeutic target.

## Methods

### Chemistry

Only the syntheses of the most potent **type I–V** inhibitors (i.e., compounds **2**, **3**, **4**, **5**, **6**) are described in this section. Those for all of the novel compounds are described in the Supplementary Methods.

#### Synthesis of intermediates

The syntheses of intermediates **9**, **10**, **11**, and **12** (see Supplementary Figures 1, 2, 3, and 5) were achieved according to the relevant literature procedures^28^,^66^.

### Biology

#### *In-vitro* inhibitory activity against the ChEs

The inhibitory potencies of the compounds against the ChEs were determined using the method of Ellman^32^. The assay protocol and IC_50_ calculations are described in the Supplementary Methods.

#### Kinetic studies for huBChE inhibition

The mode of action of huBChE inhibition by pure enantiomers of compound **2** was determined as previously described^28^. Briefly, the time-course of yellow color formation was followed on a Bio-logic SFM-2000 stopped flow apparatus at 25 °C. The two buffer solutions were prepared as one that contained the substrate butyrylthiocholine iodide, the 5,5′-dithiobis (2-nitrobenzoic acid) reagent (Ellman reagent), and test compound **2** (each pure enantiomer), and the other that contained huBChE. The buffer solutions were injected into a mixing chamber using syringes. The resulting solution contained 44 μM butyrylthiocholine iodide, 1 mM Ellman reagent, 8.2 nM huBChE, 50 nM test compound, and 0.007 M DMSO (i.e., 0.05% v/v). The absorbance was followed immediately at 412 nm, and until the change reached zero. The progress curves obtained were analyzed simultaneously, using the ENZO application^36^, which can derive and numerically solve a system of differential equations, and fit their coefficients. Several reaction mechanisms were tested. The simplest one for the reproduction of the progress curves in the absence and presence of (**+**)**-2** and **(−)-2** was chosen, and the corresponding inhibition constants were determined. The results file can be accessed by loading the ENZO project ID http://enzo.cmm.ki.si/kinetic.php?uwd=151006561&load=true, selecting the ‘Set Parameters’ tab, and pressing ‘Start’.

#### Crystallization, data collection, and processing

Crystallization, data collection, and processing are described in Supplementary Methods.

#### Cell culture and treatments

The HepG2 cell line was obtained from American Type Culture Collection (LGC Standards, UK) and was cultured in RPMI 1640 medium (Sigma-Aldrich, St. Louis/MO, USA) supplemented with 10% fetal bovine serum (Gibco, Grand Island/NY, USA), 2 mM L-glutamine, 100 U mL^−1^ penicillin and 100 μg mL^−1^ streptomycin (all from Sigma-Aldrich) in a humidified chamber at 37 °C and 5% CO_2_. Compound **2** was used at 0.625 μM to 125 μM, in DMSO.

Human neuroblastoma SH-SY5Y cells were obtained from American Type Culture Collection (CRL-2266, Manassas, VA, USA). They were grown in Dulbecco’s modified Eagle’s medium (Sigma, St. Louis, MO) supplemented with 10% fetal bovine serum (HyClone, Logan, UT, USA), 2 mM L-glutamine, 50 U mL^−1^ penicillin and 50 μg mL^−1^ streptomycin (Sigma, St. Louis, MO, USA), in a humidified atmosphere of 95% air and 5% CO_2_ at 37 °C, and grown to 80% confluence. Prior to cell treatments, complete medium was replaced with reduced-serum medium (i.e., with 2% fetal bovine serum). Compound **2** was prepared as a stock solution of 10 mM in DMSO and was used at concentrations of 1 μM to 100 μM. For the cytotoxic stimuli, Aβ_(1–42)_ was dissolved in DMSO to give a 1 mM stock solution and 24 h prior cell treatment, the peptide was incubated at final concentration of 5 μM in reduced-serum medium in the absence and presence of compound **2** (1–10 μM) at 37 °C, to induce Aβ aggregation. The metabolic activity and cell viability assays and the assessment of cytotoxicity are described in Supplementary Methods.

#### Aβ_(1–42)_ aggregation inhibitory activity

The thioflavin-T (ThT) fluorometric and dot-blot assays used are described in Supplementary Methods.

#### Inhibition of BChE in rat brain slices

##### Animals

Adult male Wistar rats (500–600 g; The Wistar Institute, Philadelphia, PA, USA) were used in this study. The rats were handled according to the European Community Council Directive of 23 November 1986 (86/609/EEC), and the National Veterinary Institute guide for the Care and Use of Laboratory Animals. All efforts were made to minimize the number of rats used and their suffering.

##### Preparation of brain tissue

The rats were sacrificed by decapitation under CO_2_ anesthesia. The brain of each animal was rapidly removed and immediately frozen on dry-ice powder, wrapped in Parafilm to prevent desiccation, and stored at −80 °C. Before cutting, the brains were allowed to equilibrate with the temperature of the cryostat chamber that had been adjusted to −20 °C. Brains were cut into 16 serial sections of 20 μm thickness in a coronal plane on a Leica SM2000R microtome with a Physitemp freezing stage and a BFS-30TC controller. The cytoarchitectonic parcellation of the brain areas was guided by the atlas of the adult rat brain by Paxinos and Watson^67^. Each section was thaw-mounted onto an RNAse-free glass slide coated with a 0.01% solution of (poly)L-lysine in dimethylpyrocarbonate, fixed for 5 min in 4% paraformaldehyde, and rinsed (3 × 3 min) in phosphate-buffered saline (PBS) with potassium. Sections were stained for BuChE or AChE using histochemical techniques^37^. The ChE histochemistry and the analysis of BuChE staining of brain structures were as described in the Supplementary Methods.

#### *In-vitro* pharmacokinetic of compound **2**

Caco-2 monolayer bidirectional permeability assay is described in detail in the Supporting Methods. In solution properties, *in vitro* metabolism assays, and identification and characterization of metabolites of compound **2** (in the form of its hydrochloride salt) when incubated with cryopreserved human hepatocytes were performed by Eurofins Panlab (St. Charles, MO, USA), in study no. 100028035. In each experiment the respective reference compounds were tested concurrently with test compound **2** and the data were compared with historical values determined at Eurofins. The experiment was accepted in accordance with Eurofins validation Standard Operating Procedure. Details on these experiments are described in the Supporting Information.

#### *In-vivo* blood plasma to brain distribution

##### Animals

The blood plasma–brain distribution of compound **2** was measured in 3-month-old female Wistar Han rats with body weight of 220 g ± 10%. The rats were handled according to the European Community Council Directive of 23 November 1986 (86/609/EEC), and the National Veterinary Institute guide for the Care and Use of Laboratory animals. The experimental design was evaluated by the National Ethical Committee for Animal Experimentation and approved by the Veterinary Administration of the Republic of Slovenia. All efforts were made to minimize the number of rats used and their suffering.

##### Chemicals used in the BBB assay

Here, 10 mg mL^−1^ compound **2** was dissolved in 1% Tween 80 isotonized with sodium chloride, and sterilized by filtration. Isotonized 1% Tween 80 was used as the negative control, and 2 mg mL^−1^ donepezil in the same solution was used as the positive control.

##### *In-vivo* blood plasma–brain distribution assay protocol, sample collection and work up

Four rats were given compound **2** and three rats were used for each of the positive and negative controls. The IP injected doses of compound **2** and donepezil were 10 mg kg^−1^ and 2 mg kg^−1^, respectively. The rats were euthanized by CO_2_ inhalation 1 h after administration of compound **2**. Immediately, 2 mL blood was collected in Vacutainer tubes with 10.8 mg K_2_EDTA, and the brain tissue was isolated. Blood plasma was obtained after centrifugation of the blood at 3,500 rpm for 10 min. The blood plasma and brain tissue were stored at −20 °C until further sample preparation.

Four aliquots of approximately 150 mg of each brain tissue sample were weighted and homogenized in 2-mL microcentrifuge tubes (Eppendorf, Germany) with 25 μL internal standard solution (450 μg L^−1^ haloperidol in 80% MeOH with 0.1% acetic acid), 300 μL PBS, and 125 μL methanol, in a blender (Bullet Blender; Next Advance, NY, USA) for 3 min at speed setting ‘8’ with 0.5-mm glass beads. The homogenate was centrifuged at 10,000 rpm for 7 min, and the supernatant was removed. The sample was mixed again in the blender for 1 min at speed setting ‘5’, and a second supernatant was collected after centrifugation as before. These supernatants were then combined, and 900 μL 2% H_3_PO_4_ was added prior to solid phase extraction (SPE).

Blood plasma aliquots (150 μL) in 2-mL microcentrifuge tubes (Eppendorf) were mixed with 25 μL internal standard solution (450 μg L^−1^ haloperidol in 80% MeOH with 0.1% acetic acid), 300 μL PBS and 125 μL MeOH. The samples were mixed in the blender for 1 min at minimum speed, and sonicated in an ultrasound bath for 5 min. Then 900 μL 2% H_3_PO_4_ was added in two aliquots during the quantitative sample transfer to the SPE column.

For the preparation of all of the samples for LC-MS/MS quantification, Bond Elut Plexa PCX (30 mg, 3 mL) SPE columns were used. The SPE columns were conditioned with 2 mL methanol and equilibrated with 2 mL water before the samples were slowly loaded. Then 2 mL 2% formic acid, followed by 2 mL MeOH/MeCN mixture (1:1, v/v) were used for column washing. The samples were eluted with 1 mL 25% ammonia in water/MeOH/MeCN (1:2:2, v/v), and dried for 20 min in a concentration evaporator (Turbovap) at 60 °C. The dried samples were stored at −20 °C until analysis. The samples were reconstituted in 200 μL 0.1% acetic acid in 80% MeOH using 2 × 2-min mixing in the blender at minimum speed, separated by a 10-min waiting interval.

For the calculation of the brain-to-plasma ratio of compound **2** its concentration in the brain which is given in μg kg^−1^ and its concentration in blood plasma which is given in μg L^−1^ the specific density of wet rat brain (1.05 g mL^−1^) was used^68^. LC-MS/MS analysis and LC-MS/MS method validation are described in the Supplementary Methods.

#### *In-vivo* activity assays

##### Animals

Eight-week-old male Albino Swiss (CD–1 Krf) mice weighing from 18 g to 22 g were used in the passive avoidance task, and the locomotor activity and rotarod tests, and C57BL/6 J mice of the same age were used in the MWM and the RAWM tasks. The mice were housed in groups of 10 per cage at room temperature (22 ± 2 °C) under a light/dark (12 h:12 h) cycle. The mice had free access to food and water, and the ambient temperature and humidity of the room were kept constant throughout all of the tests. For the behavioral tasks, the mice were selected at random, and each group consisted of 8 to 10 mice dose^−1^. The experiments were performed between 08:00 and 14:00. Immediately after these *in-vivo* assays, the animals were euthanized by cervical dislocation. The maintenance and treatment of the laboratory animals were carried out in full accordance with the guidelines issued by the Local Ethics Committee of the Jagiellonian University in Krakow (ZI/862/2013). All efforts were made to minimize the number of mice used and their suffering.

##### Chemicals used in the *in-vivo* activity assays

For the *in-vivo* experiments, compound **2** was suspended in 1% Tween 80 (Polskie Odczynniki Chemiczne, Poland), with IP injections 60 min before the acquisition trial of learning and memory tasks, and the locomotor activity and rotarod tests. Rivastigmine hydrogen tartrate was from Sigma Aldrich (Poland), and it was dissolved in distilled water and administered by the same route as compound **2**. Control mice were given an appropriate amount of vehicle (1% Tween 80, IP). **(−)**-Scopolamine hydrobromide was from Sigma Aldrich (Poland). To induce memory impairment, scopolamine was dissolved in distilled water, with IP administration at 1 mg kg^−1^ 30 min before the acquisition trial of each learning and memory task. Vehicle-treated mice were used as the negative control. For comparison, the same experiments were performed in mice similarly treated with scopolamine and the anti-AD drug rivastigmine, which served as the positive control in these *in-vivo* assays. The behavioral testing paradigms are described in the Supplementary Methods.

## Additional Information

**How to cite this article:** Košak, U. *et al*. Development of an *in-vivo* active reversible butyrylcholinesterase inhibitor. *Sci. Rep.* **6**, 39495; doi: 10.1038/srep39495 (2016).

**Publisher's note:** Springer Nature remains neutral with regard to jurisdictional claims in published maps and institutional affiliations.

## Supplementary Material

Supplementary Information

## Figures and Tables

**Figure 1:**
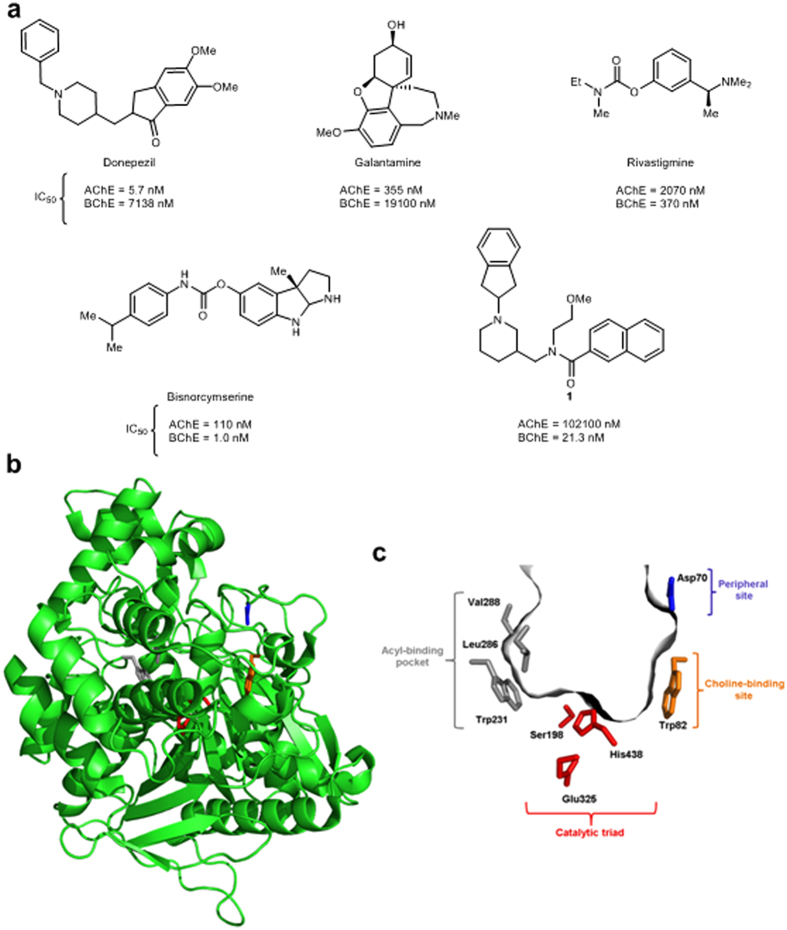
ChE inhibitors and structures of huBChE and its active site gorge. (**a**) Structures and IC_50_ values against the ChEs of the currently approved drugs for the treatment of AD symptoms that exploit ChE inhibition (donepezil, galantamine, rivastigmine) and selective huBChE inhibitors (bisnorcymserine and compound **1**). (**b**) The overall structure of huBChE (PDB code 4TPK) shown as a green cartoon with the key amino acids of the active site shown as sticks. (**c**) The active site gorge of huBChE shown as a gray surface. The principal contributors to the peripheral site (Asp70; blue), the choline-binding site (Trp82; orange), the catalytic triad (Ser198, Glu325, His438; red), and the acyl-binding pocket (Trp231, Leu286, Val288; gray) are shown as sticks.

**Table 1:**
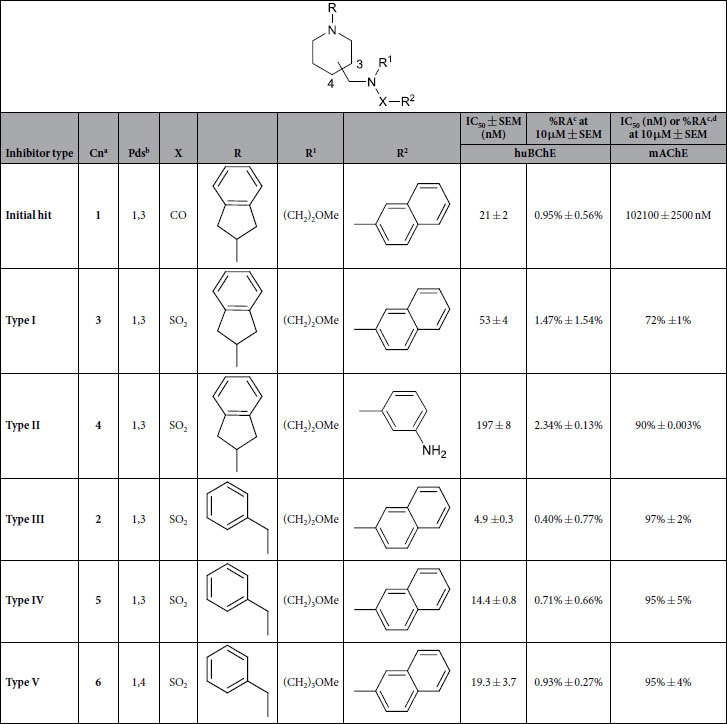
Inhibitory potencies and structures of hit compound 1 and the most potent of the type I to V inhibitors.

^a^**Cn** = compound number.^b^**Pds** = piperidine disubstitution pattern.^c^**RA** = residual activity.^d^**%RA** > 75% was considered as no inhibition.
