# Supplementary material for: Development of an in-vivo active reversible butyrylcholinesterase inhibitor
Source: Sci Rep. 2016 Dec 21;6:39495. doi: 10.1038/srep39495 (PMC5175178; doi:10.1038/srep39495)
Supplement: Supplementary Information [file srep39495-s1.doc]

***Supplementary information***

**Development of an *in-vivo* active reversible butyrylcholinesterase inhibitor**

Urban Košak,^‡^ Boris Brus,^‡^ Damijan Knez,^‡^ Roman Šink,^‡^ Simon Žakelj,^‡^ Jurij Trontelj,^‡^ Anja Pišlar,^‡^ Jasna Šlenc,^‡^ Martina Gobec,^‡^ Marko Živin,^▲^ Larisa Tratnjek,^▲^ Martina Perše,^●^ Kinga Sałat, ^∆^ Adrian Podkowa, ^∆^ Barbara Filipek, ^∆^ Florian Nachon,^ø^ Xavier Brazzolotto,^ø^ Anna Więckowska,^∆^ Barbara Malawska,^∆^ Jure Stojan,^■^ Irena Mlinarič Raščan,^‡^ Janko Kos,^‡^ Nicolas Coquelle, ^§‌‌, Ө,▼‌‌‌‌^ Jacques-Philippe Colletier^§‌‌,Ө,▼^,* and Stanislav Gobec^‡^,*

^‡^Faculty of Pharmacy, University of Ljubljana, Aškerčeva 7, 1000 Ljubljana, Slovenia

^▲^Institute of Pathological Physiology, Faculty of Medicine, University of Ljubljana, Vrazov trg 2, 1000 Ljubljana, Slovenia

^●^Institute of Pathology, Faculty of Medicine, University of Ljubljana, Korytkova 2, 1000 Ljubljana, Slovenia

*^ø^* Institut de Recherche Biomédicale des Armées, 91223 Brétigny sur Orge, France

*^∆^* Faculty of Pharmacy, Jagiellonian University, Medyczna 9 St., 30-688 Krakow, Poland

^■^Institute of Biochemistry, Faculty of Medicine, University of Ljubljana, Vrazov trg 2, 1000 Ljubljana, Slovenia

^§^University Grenoble Alpes, IBS, F-38044 Grenoble, France

^Ө^CNRS, IBS, F-38044 Grenoble, France

^▼^CEA, IBS, F-38044 Grenoble, France

**1. SUPPLEMENTARY FIGURES**

Supplementary Figure 1. Synthesis of type I inhibitor 3 and type II inhibitors 4, 13, 16-25. Conditions and reagents: (a) R-SO_2_-Cl, Et_3_N, DCM, 0 °C to rt, 24 h (3, 93%; 16, 92%; 17, 92%; 18, 59%; 19, 72%; 20, 68%; 13, 40%; 21, 82%). (b) NiCl_2_×6H_2_O, NaBH_4_, THF/MeOH (1:1, v/v), 0 °C (2 h) to rt (16 h) (22, 81%; 4, 57%). (c) Ac_2_O, pyridine, rt, 16 h (23, 98%; 24, 96%). (d) MeNH_2_ solution in EtOH, sealed reaction tube, rt to 80 °C, 10 days (25%).

Supplementary Figure 2. Synthesis of type III inhibitors 2, 37-41 and inhibitors 42, 43.

Conditions and reagents: (a) R-CHO, NaBH(OAc)_3_, AcOH, DCE, rt, under argon, 24 h (27, 79%; 28, 63%; 29, 79%, 30, 77%; 31, 66%). (b) TFA, DCM, rt, 24 h (32, 94%; 33, 92%; 34, 93%; 35, 90%; 36, 91%). (c) Naphthalene-2-sulfonyl chloride, Et_3_N, DCM, 0 °C to rt, 24 h (2, 90%, 37, 72%; 38, 87%; 39, 92%; 40, 97%; 41, 95%). (d) Naphthalene-1-sulfonyl chloride, Et_3_N, DCM, 0 °C to rt, 24 h (79%). (e) 7-Methoxynaphthalene-2-sulfonyl chloride, Et_3_N, DCM, 0 °C to rt, 24 h (92%). (f) 4 M HCl in 1,4-dioxane, Et_2_O/1,4-dioxane (5:1, v/v), 0 °C to rt, under argon, 30 min (97%).

Supplementary Figure 3. Synthesis of type IV inhibitors 7, 8, 46-48 and inhibitor 49. Conditions and reagents: (a) Naphthalene-2-sulfonyl chloride, Et_3_N, DCM, 0 °C to rt, 24 h (7, 43%; 5, 87%). (b) R-X, Cs_2_CO_3_, MeCN, NaI (cat.), rt to 60 °C, under argon, 1 h to 6 h (8, 70%; 46, 73%, 47, 82%; 48, 83%). (c) 7-(cyanomethoxy)naphthalene-2-sulfonyl chloride, Et_3_N, DCM, 0 °C to rt, 24 h (75%).

Supplementary Figure 4. Synthesis of type IV inhibitors 64-69, 71, 72. Conditions and reagents: (a) R-CH_2_-Cl, K_2_CO_3_, anhydrous DMF, rt, 20 h (52, 66%; 53, 31%; 54, 55%, 55, 69%). (b) R-CHO, NaBH(OAc)_3_, AcOH, DCE, rt, under argon, 24 h (56, 39%; 57, 62%). (c) TFA, DCM, rt, 24 h (58, 93%; 59, 92%; 60, 91%; 61, 94%; 62, 93%; 63, 93%). (d) Naphthalene-2-sulfonyl chloride, Et_3_N, DCM, 0 °C to rt, 24 h (64, 37%; 65, 86%; 66, 98%; 67, 61%; 68, 76%, 69, 66%; 71, 51%). (e) MeI, Cs_2_CO_3_, MeCN, rt to 60 °C, under argon, 1 h (54%).

Supplementary Figure 5. Synthesis of type V inhibitors 6, 75, 84, 85, 86. Conditions and reagents: (a) Naphthalene-2-sulfonyl chloride, Et_3_N, DCM, 0 °C to rt, 24 h (75, 88%; 6, 86%; 84, 75%; 85, 59%; 86, 73%). (b) Cyclohexene, Pd(OH)_2_/C cat., MeOH, rt to reflux, under argon, 20 h (78, 96%; 79, 94%). (c) 1*H*-Inden-2(3*H*)-one, NaBH(OAc)_3_, AcOH, DCE, rt, under argon, 24 h (80, 59%; 81, 67%). (d) TFA, DCM, rt, 24 h (82, 85%; 83, 88%).


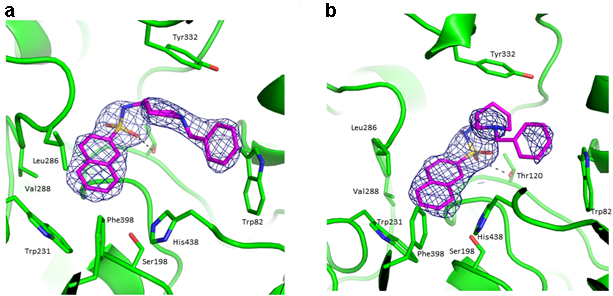


Supplementary Figure 6. Crystal structures of huBChE in complex with compounds 7 and 8. Compounds (a) 7 and (b) 8 are shown as purple sticks covered by experimental 2m*Fo*–D*Fc* electron density maps (blue mesh, contoured at 1*σ*). The H-bonds between the oxygen of the sulfonamide moiety and Thr120 are shown as yellow dashes (distances 2.8, 3.4 Å for 7, 8, respectively). Important residues in the huBChE active site are shown as green sticks.


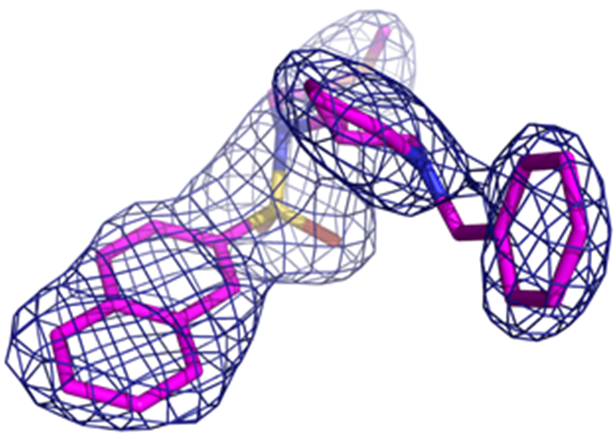


Supplementary Figure 7. 2m*Fo*–D*Fc* electron density map (blue mesh, contoured at 1 *σ*) of compound 2 (purple sticks) bound in the active site of huBChE.


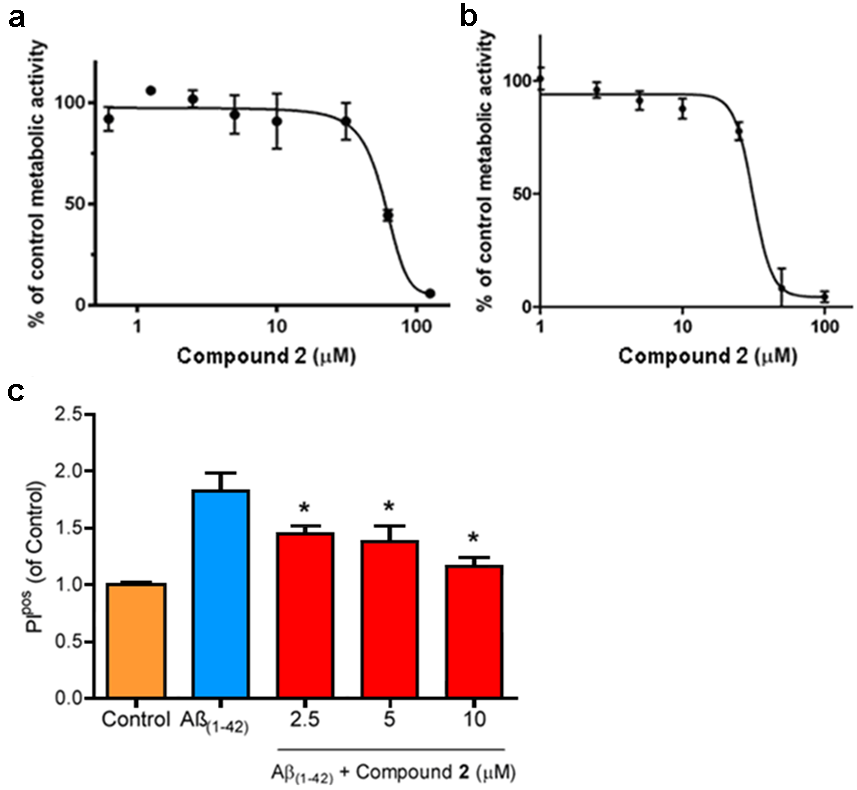


Supplementary Figure 8. *In-vitro* toxicity profile and neuroprotective effects of compound 2. Concentration-dependent toxicity of compound 2, as cytotoxicity in human HepG2 cells (a) and neurotoxicity in human neuroblastoma SH-SY5Y cells (b). The cells were incubated in the presence of the indicated concentrations of compound 2. After 24 h (a) or 48 h (b), the cell viability was evaluated using the MTS assay. The control group (DMSO) was considered as 100% cell viability. Data are means ±SD of at least three independent experiments, each carried out in triplicate (a) and quadruplicate (b). (c) Neuroprotective effects of compound 2 on Aβ_(1–42)_-induced cytotoxicity in human neuroblastoma SH-SY5Y cells. The cells were treated with Aβ_(1–42)_ (5 µM) in the absence or presence of increasing concentrations of compound 2 (2.5, 5, 10 µM). After 48 h of treatment, the cells were harvested and stained with PI, and the percentages of PI positive (PI^pos^) cells relative to control cells (DMSO control) were determined with flow cytometry. Data are means ±SD of three independent assays. *, *P* <0.05.

Supplementary Figure 9. Possible identities of hepatic metabolites for compound 2.


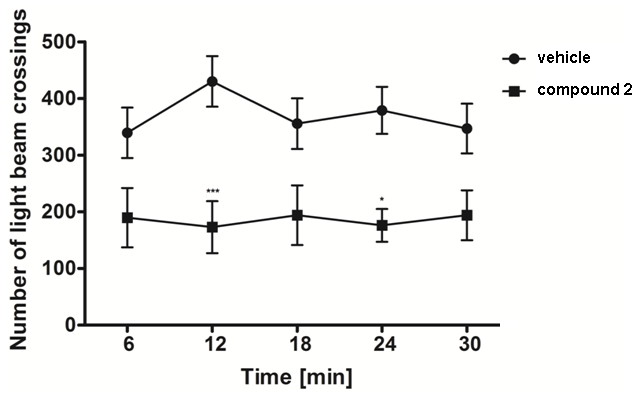


Supplementary Figure 10. Influence of compound 2 (30 mg kg^–1^) on mouse locomotor activity. Data are means ±SEM. The vehicle and the test compound were administered intraperitoneally, 1 h before the assay. Statistical analysis of the data was performed using repeated measures analysis of variance (ANOVA), followed by Bonferroni multiple comparisons. Drug effect: F[1,56]=10.70; p <0.01. Time effect: F[4,56]=0.8; p >0.05. Time × drug interaction: F[4,56]=2.07; p >0.05. Significance compared to vehicle-treated mice at the respective time points: * p <0.05, *** p <0.001.

**2.** **SUPPLEMENTARY TABLES**

Supplementary Table 1. Inhibitory structures and potencies of hit compound 1 and all of the synthesized sulfonamide inhibitors.

|  | | | | | | | | |
| --- | --- | --- | --- | --- | --- | --- | --- | --- |
| **It**^a^ | **Cn**^b^ | **Pds**^c^ | **X** | **R** | **R^1^** | **R^2^** | **IC_50_ or %RA**^d^ **at 10 µM ±SEM** | **IC_50_ or %RA**^d,e^ **at 10 µM ±SEM** |
|  |  |  |  |  |  |  | **huBChE** | **mAChE** |
| **Initial hit** | **1** | 1,3 | CO |  | (CH_2_)_2_OMe |  | 21 ±2 | 102100 ±2500 nM |
| **Type I** | **3** | 1,3 | SO_2_ |  | (CH_2_)_2_OMe |  | 53 ±4 | 72% ±1% |
| **Type II** | **4** | 1,3 | SO_2_ |  | (CH_2_)_2_OMe |  | 197 ±8 | 90% ±0.003% |
| **Type II** | **13** | 1,3 | SO_2_ |  | (CH_2_)_2_OMe |  | 3611 ±528 | 68% ±4% |
| **Type II** | **16** | 1,3 | SO_2_ |  | (CH_2_)_2_OMe |  | 343 ±5 | 97% ±4% |
| **Type II** | **17** | 1,3 | SO_2_ |  | (CH_2_)_2_OMe |  | 279 ±8 | 86% ±3% |
| **Type II** | **18** | 1,3 | SO_2_ |  | (CH_2_)_2_OMe |  | 887 ±80 | 88% ±2% |
| **Type II** | **19** | 1,3 | SO_2_ |  | (CH_2_)_2_OMe |  | 482 ±66 | 87% ±7% |
| **Type II** | **20** | 1,3 | SO_2_ |  | (CH_2_)_2_OMe |  | 3466 ±52 | 91% ±1% |
| **Type II** | **21** | 1,3 | SO_2_ |  | (CH_2_)_2_OMe |  | 359 ±18 | 94% ±0.001% |
| **Type II** | **22** | 1,3 | SO_2_ |  | -(CH_2_)_2_OMe |  | 314 ±36 | 91% ±1% |
| **Type II** | **23** | 1,3 | SO_2_ |  | (CH_2_)_2_OMe |  | 321 ±35 | 85% ±5% |
| **Type II** | **24** | 1,3 | SO_2_ |  | (CH_2_)_2_OMe |  | 8207 ±601 | 95% ±4% |
| **Type II** | **25** | 1,3 | SO_2_ |  | (CH_2_)_2_OMe |  | 3225 ±492 | 101% ±4% |
| **Type III** | **2** | 1,3 | SO_2_ |  | (CH_2_)_2_OMe |  | 4.9 ±0.3 | 97% ±2% |
| **Type III** | **37** | 1,3 | SO_2_ |  | (CH_2_)_2_OMe |  | 23.1 ±6.0 | 89% ±3% |
| **Type III** | **38** | 1,3 | SO_2_ |  | (CH_2_)_2_OMe |  | 49.1 ±1.2 | 99% ±0.001% |
| **Type III** | **39** | 1,3 | SO_2_ |  | (CH_2_)_2_OMe |  | 1464 ±115 | 97% ±2% |
| **Type III** | **40** | 1,3 | SO_2_ |  | (CH_2_)_2_OMe |  | 338 ±8 | 93% ±3% |
| **Type III** | **41** | 1,3 | SO_2_ |  | (CH_2_)_2_OMe |  | 311 ±22 | 105% ±6% |
| **Type IV** | **5** | 1,3 | SO_2_ |  | (CH_2_)_3_OMe |  | 14.4 ±0.8 | 95% ±5% |
| **Type IV** | **7** | 1,3 | SO_2_ |  | H |  | 56.9 ±9.8 | 78% ±3% |
| **Type IV** | **8** | 1,3 | SO_2_ |  | Me |  | 25 ±3.8 | 82% ±1% |
| **Type IV** | **46** | 1,3 | SO_2_ |  | Et |  | 38 ±2.6 | 93% ±2% |
| **Type IV** | **47** | 1,3 | SO_2_ |  | *n*-Pr |  | 42.8 ±6.4 | 84% ±3% |
| **Type IV** | **48** | 1,3 | SO_2_ |  | *n*-Bu |  | 35.5 ±4.4 | 90% ±1% |
| **Type IV** | **64** | 1,3 | SO_2_ |  | H |  | 1673 ±256 | 95% ±5% |
| **Type IV** | **65** | 1,3 | SO_2_ |  | H |  | 336 ±31 | 71% ±4% |
| **Type IV** | **66** | 1,3 | SO_2_ |  | H |  | 287 ±22 | 57% ±4% |
| **Type IV** | **67** | 1,3 | SO_2_ |  | H |  | 347 ±60 | 73% ±4% |
| **Type IV** | **68** | 1,3 | SO_2_ |  | H |  | 583 ±43 | 75% ±4% |
| **Type IV** | **69** | 1,3 | SO_2_ |  | H |  | 2093 ±328 | 93% ±5% |
| **Type IV** | **71** | 1,3 | SO_2_ |  | H |  | 30% ±2% | 95% 0.001% |
| **Type IV** | **72** | 1,3 | SO_2_ |  | Me |  | 427 ±44 | 96% ±2% |
| **Type V** | **6** | 1,4 | SO_2_ |  | (CH_2_)_3_OMe |  | 19.3 ±3.7 | 95% ±4% |
| **Type V** | **75** | 1,4 | SO_2_ |  | (CH_2_)_2_OMe |  | 39.4 ±2.8 | 98% ±1% |
| **Type V** | **84** | 1,4 | SO_2_ |  | (CH_2_)_2_OMe |  | 52.5 ±2.0 | 87% ±3% |
| **Type V** | **85** | 1,4 | SO_2_ |  | (CH_2_)_3_OMe |  | 71.2 ±1.9 | 86% ±0.004% |
| **Type V** | **86** | 1,4 | SO_2_ |  | H |  | 195 ±17 | 1780 ±85 nM |
| **/** | **42** | 1,3 | SO_2_ |  | (CH_2_)_2_OMe |  | 59 ±5.4 | 94% ±4% |
| **/** | **43** | 1,3 | SO_2_ |  | (CH_2_)_2_OMe |  | 156 ±33 | 95% ±7% |
| **/** | **49** | 1,3 | SO_2_ |  | (CH_2_)_3_OMe |  | 113 ±8 | 93% ±3% |

^a^**It** = inhibitor type

^b^**Cn** = compound number

^c^**Pds =** piperidine disubstitution pattern

^d^**RA** = residual activity

^d,e^%RA >75% was considered no inhibition

**Supplementary Table 2. Characteristic constants for the hydrolysis of butyrylthiocholine iodide by huBChE, and its inhibition by (–)-2 and (+)-2** at pH 8.

| **Constant** | **BTCI**^a^ | **(–)-2** | **(+)-2** |
| --- | --- | --- | --- |
| K_m_ (µM) ± SD^b^ | 38.5 ± 0.3 |  |  |
| k_cat_ (s^-1^) ± SD^b^ | 852 ± 8 |  |  |
| K_i_ (nM) ± SD^b^ |  | 1.29 ± 0.03 | 2.01 ± 0.05 |

^a^BTCI = butyrylthiocholine iodide

^b^SD = standard deviation

**Supplementary Table 3.** **Data collection and refinement statistics.**

| **Parameter** | **Compound 7** | **Compound 8** | **Compound 2** |
| --- | --- | --- | --- |
| PDB code | **5DYY** | **5DYT** | **5DYW** |
| Wavelength (Å) | 0.87260 | 0.87260 | 0.93903 |
| Resolution range (Å) | 46.21 – 2.65  (2.745 – 2.65) | 46.12 – 2.55  (2.641 – 2.55) | 48.2 – 2.50  (2.589 – 2.50) |
| Space group | P 21 21 21 | P 21 21 21 | P 21 21 21 |
| Unit cell | 75.97  80.19  232.91  90, 90, 90 | 76.47  80.23  231.28  90, 90, 90 | 72.21  78.86  226.65  90, 90, 90 |
| Total reflections | 279725 (27639) | 248718 (25878) | 335996 (33412) |
| Unique reflections | 42160 (4154) | 47154 (4671) | 45699 (4481) |
| Multiplicity | 6.6 (6.7) | 5.3 (5.5) | 7.4 (7.5) |
| Completeness (%) | 99.77 (99.90) | 99.66 (99.98) | 99.99 (99.96) |
| Mean I/sigma(I) | 13.32 (2.45) | 13.47 (2.18) | 12.48 (1.60) |
| Wilson B-factor | 51.27 | 56.52 | 44.15 |
| R-merge (%) | 0.1103 (0.671) | 0.0807 (0.7319) | 0.1596 (1.369) |
| R-meas (%) | 0.1198 | 0.08967 | 0.1717 |
| CC1/2 | 0.997 (0.804) | 0.998 (0.760) | 0.996 (0.673) |
| CC* | 0.999 (0.944) | 0.999 (0.929) | 0.999 (0.897) |
| R-work | 0.1863 (0.2581) | 0.1824 (0.2554) | 0.1898 (0.2951) |
| R-free | 0.2414 (0.3188) | 0.2391 (0.3320) | 0.2160 (0.3230) |
| Number of non-hydrogen atoms | 9015 | 9069 | 9234 |
| macromolecules | 8376 | 8382 | 8382 |
| ligands | 406 | 455 | 567 |
| Protein residues | 1052 | 1058 | 1052 |
| RMS(bonds) | 0.009 | 0.009 | 0.007 |
| RMS(angles) | 1.03 | 1.00 | 1.86 |
| Ramachandran favored (%) | 95.0 | 95.0 | 95.0 |
| Ramachandran allowed (%) | 4.3 | 4.4 | 5.0 |
| Ramachandran outliers (%) | 0.7 | 0.6 | 0.0 |
| Rotamer outliers (%) | 1.3 | 1.6 | 0.89 |
| Clashscore | 5.33 | 5.42 | 7.39 |
| Average B-factor | 48.84 | 57.96 | 47.93 |
| macromolecules | 47.32 | 56.09 | 46.27 |
| ligands | 82.73 | 97.12 | 74.32 |
| solvent | 44.25 | 52.52 | 44.19 |
| Surface occupied by compound in active site gorge (Å^2^)^a^ | 341.0 ±6.65 | 304.9 ±1.48 | 302.4 ±11.2 |
| Average per-residue interacting area in binding site (Å^2^) | 15.02 | 13.91 | 13.49 |
| Surface complementarity between compound and active site gorge^a^ | 0.743 | 0.734 | 0.715 |

Statistics for the highest-resolution shell are shown in parentheses.

^a^, as averaged over the two monomers present in the asymmetric unit

Supplementary Table 4. Aβ_1–42_ aggregation inhibitory activity of compound 2. The results are expressed as average Aβ_1–42_ aggregation inhibition ±SEM of at least two independent experiments.

| **Assay** | **Percent inhibition ±SEM (%)** |
| --- | --- |
| *ThT* | –4.89 ±5.30 |
| *Dot blot (LOC antibody)* | 12.67 ±8.90 |
| *Dot blot (OC antibody)* | 2.70 ±9.32 |

Supplementary Table 5. Human plasma protein binding.

| **Compound** | **Protein Bound**  **(%)** | | | | **Recovery**  **(%)** | | | |
| --- | --- | --- | --- | --- | --- | --- | --- | --- |
|  | **1^st^** | **2^nd^** | **Mean** |  | | **1^st^** | **2^nd^** | **Mean** |
| Compound **2** | 96.0 | 95.3 | **96** |  | | 101 | 99 | **100** |
| Acebutolol | 20.8 | 13.5 | **17** |  | | 127 | 129 | **128** |
| Quinidine | 68.5 | 69.1 | **69** |  | | 114 | 114 | **114** |
| Warfarin | 98.5 | 98.6 | **99** |  | | 112 | 109 | **110** |

Supplementary Table 6. Human plasma half-life (t_1/2_).

| **Compound** | **Half-Life t_1/2_ (min)** | | |
| --- | --- | --- | --- |
|  | **1^st^** | **2^nd^** | **Mean** |
| Compound **2** | 223.1 | 206.7 | **>120** |
| Propantheline | 17.9 | 18.0 | **18** |
| Propoxycaine | 3.3 | 3.0 | **3** |

Supplementary Table 7. Cryopreserved human hepatocytes half-life (t_1/2_) and intrinsic clearance (Cl_int_) of compound 2.

| **Compound** | **Half-Life t_1/2_ (min)** | | | | **Cl_int_ (µL/min/million cells)** |
| --- | --- | --- | --- | --- | --- |
|  | **1^st^** | **2^nd^** | **2^rd^** | **Mean** |  |
| Compound **2** | 55.7 | 51.7 | 55.6 | **54** | **18.2** |
| HFC | 40.4 | 39.3 | 39.9 | **40** | **24.8** |
| Flurazepam | 43.3 | 44.1 | 45.0 | **44** | **22.4** |
| Naloxone | 51.5 | 52.0 | 52.6 | **52** | **19** |
| Propranolol | 90.9 | 84.8 | 87.6 | **88** | **11.3** |

Supplementary Table 8. Putative metabolites identified from full-scan analysis of the test compound incubate for 2 h with human cryopreserved hepatocytes.

| **Compound** | **Possible modification** | **Observed Precursor Ion (m/z)** | **Observed Retention Time (min)** | **Major Product Ions (m/z)** |
| --- | --- | --- | --- | --- |
| Compound **2** | Parent | 453.22 | 4.92 | 361, 278, 188, 91 |
|  | Debenzylation | 363.17 | 4.18 | 216, 120, 63 |
|  | Demethylation + glucoronidation | 615.23 | 4.31 | 439, 386, 129 |
|  | Demethylation | 439.20 | 4.50 | 366, 204, 129 |

Supplementary Table 9. Influence of compound 2 on mouse motor coordination in the rotarod test.

| **Treatment** | **Dose**  **(mg kg^-1^)** | **Time spent on the rotarod, according to rotation speed (s ±SEM)** | | |
| --- | --- | --- | --- | --- |
|  |  | **6 rpm** | **18 rpm** | **24 rpm** |
| Vehicle | - | 60.0 | 60.0 | 60 |
| Compound **2** | 30 | 60.0 | 60.0 | 60 |
|  | 100 | 60.0 | 53.3 ±6.8 | 52.3 ±6.8 |

Data are means ±SEM.

Statistical analysis: one-way ANOVA, followed by Dunnett’s *post-hoc* comparisons. 18 rpm: F[2,21] = 1.000, p >0.05; 24 rpm: F[2,21] = 1.297, p >0.05. Significance vs. vehicle-treated mice: not significant.

**Supplementary Table 10. Effect of compound 2 and rivastigmine on memory retention (probe trial - without drug treatment on day 7) for the Morris water maze. Results showed for n = 9 animals per group. Statistical analysis: one-way analysis of variance (ANOVA), followed by Dunnett’s *post-hoc* test. Significance vs. scopolamine-treated control. NS - not significant.**

| **Parameter analyzed** | **Latency time**  **(s ±SEM)^a^** | **Number of NW zone crossings**  **(n ±SEM)** | **Time in target (NW) quadrant**  **(s ±SEM)** | **Distance in goal quadrant**  **(% ±SEM)** | **Entries in zone NW**  **(n ±SEM)** | **Total distance covered**  **(m ±SEM)** |
| --- | --- | --- | --- | --- | --- | --- |
| Vehicle | 3.6 ±1.6 | 4.0 ±0.6 | 25.2 ±2.7 | 42.7 ±3.8 | 13.1 ±1.3*** | 10.9 ±0.3 |
| Vehicle + scopolamine | 1.3 ±0.4 | 2.4 ±0.5 | 26.7 ±2.5 | 45.4 ±3.8 | 6.4 ±0.7 | 11.3 ±0.3 |
| Scopolamine + compound **2**  (30 mg kg^–1^) | 1.2 ±0.4  F[2,25] =  1.792; NS | 2.6 ±0.5  F[2,25] =  2.182; NS | 29.3 ±1.5  F[2,25] =  0.8737; NS | 45.35 ±2.6  F[2,25] =  0.2035; NS | 7.3 ±0.7  F[2,25] =  14.59;  p <0.0001 | 11.5 ±0.6  F2,25] =  0.3439; NS |
| Scopolamine + rivastigmine  (2.5 mg kg^–1^) | 2.1 ±1.0 | 3.8 ±0.8 | 33.5 ±2.7 | 54.0 ±4.4 | 7.4 ±0.6 | 11.0 ±0.4 |
| Scopolamine + rivastigmine  (1 mg kg^–1^) | 2.1 ±0.9  F[3,33] =  0.7484; NS | 4.5 ±0.8  F[3,33] =  1.472; NS | 35.9 ±2.8  F[3.33] =  3.824;  p <0.05 | 58.6 ±4.4  F[3,33] =  3.307;  p<0.05 | 6.3 ±0.5  F[3,33] =  14.87;  p <0.0001 | 11.3 ±0.5  F[3,33] =  0.1719; NS |

^a^, Latency time: to the first crossing of the former platform location (target zone)

**3. SUPPLEMENTARY DISCUSSION**

**3.1. Structure-activity relationships of huBChE inhibitors**

The complete list of inhibitory potencies and structures of all of the huBChE inhibitors together with initial hit compound **1** is given in Supplementary Table S1. When comparing the inhibitory potencies of hit compound **1** (IC_50_ = 21 nM) with its sulfonamide bioisostere **3** (IC_50_ = 53 nM), replacing the carboxamide with a sulfonamide slightly reduced the inhibitory potency. In **type II** inhibitors, the naphthalene ring was replaced by benzene or by substituted benzene, where only small electron-donating or electron-withdrawing substituents were used. In this series, the IC_50_ values ranged from 197 nM to 8207 nM, which represented a significant drop in inhibitory potency when compared to hit compound **1** and to parent sulfonamide **3**.

Replacing the 2,3-dihydro-1*H*-inden moiety of **type I** inhibitor **3** with benzyl groups resulted in **type III** inhibitors. Here, compound **2** (with unsubstituted benzyl) improved the inhibitory potency by more than 10-fold (IC_50_ = 4.9 nM), resulting in the most potent huBuChE inhibitor in this study. Introduction of an electron-withdrawing group like flouro or cyano at positions 3 or 4 of the benzyl ring, or an electron-donating dimethylamino group at position 4 of the benzyl ring, substantially reduced the inhibitory potency (**37**-**41**; IC_50_ values from 49.1 to 1464 nM).

The importance of the *N*-alkyl chain [-(CH_2_)_2_-OMe] for the low nanomolar inhibitory potency is clear from the series of **type IV** inhibitors. Removal (**7**; IC_50_ = 56.9 nM), replacement with other linear alkyl groups (**8**, **46**-**48**; IC_50_ values from 25 to 42.8 nM), or elongation of the *N*-alkyl group (**5**; IC_50_ = 14.4 nM) reduced the inhibitory potency of the sulfonamides obtained compared to the best inhibitor **2**. **Type IV** inhibitors, in which the *N*-benzyl group of secondary sulfonamide **7** was replaced with substituted benzyl rings (**66**-**69**; IC_50_ values from 287 nM to 2093 nM), or by some heterocycles (compounds **64** [IC_50_ = 1673 nM], **65** [IC_50_ = 336 nM] and **71** [51% RA at 10µM]), were all weaker huBChE inhibitors than parent compound **7**. Additionally, methylation (**73**; IC_50_ = 427 nM) of the sulfonamide nitrogen of compound **69** did not have significant effects on the inhibitory potency.

**Type V** inhibitors are 1,4-disubstituted piperidines that were synthesized with the purpose to study the influence of piperidine disubstitution patterns. While compound **84** (IC_50_ = 52.5 µM) was just as potent as its 1,3-disubstituted piperidine analog **3**, **type V** inhibitors **6** (IC_50_ = 19.3 µM), **75** (IC_50_ = 39.4 nM), and **86** (IC_50_ = 195 nM) were weaker huBChE inhibitors than their 1,3-disubstituted counterparts **5**, **2**, and **7**, respectively. Elongation (**85**, IC_50_ = 71.2 nM) of the *N*-alkyl group in compound **84** did not have any effect on inhibitory potency.

The structure-activity relationships are consistent with observations in crystallography. The solved crystal structures of huBChE in complexes with **type III** inhibitors **2** and **7**, and **type IV** inhibitor **8**, showed that their naphthalene-2-yl moieties bind tightly in the acyl binding pocket of the enzyme (Fig. 4b), which is the basis of their good inhibitory potency. It is thus clear why all **type II** inhibitors with smaller benzene groups that do not fill the entire acyl binding pocket were weaker inhibitors than compound **2**. Analogously, inhibitors containing a 1-naphthyl ring (**42**; IC_50_ = 59 nM) or with substituents on the naphthalene-2-yl moiety (**43**; IC_50_ = 156 nM; and **49**; IC_50_ = 113 nM) were weaker inhibitors, as they do not fit tightly in the acyl binding pocket. Their perfect fit to the huBChE active site was obviously prevented by incorrect positioning of the 1-naphthyl ring (**42**) or by the steric bulk of the substituents (**42**, **49**).

**3.2. Other sulfonamide cholinesterase inhibitors**

The sulfonamide functional group has been underexploited in the discovery and development of cholinesterase inhibitors as only a limited number of sulfonamide cholinesterase inhibitors have been reported^1–9^. Those that are selective BChE inhibitors display micromolar inhibitory potencies against BChE with IC_50_ values ranging from 2.0 – 304.14 µM. None of them has been characterized beyond *in-vitro* inhibition assays^6–9^.

**4. SUPPLEMENTARY METHODS**

**4.1. Synthesis of type I inhibitor 3, and type II inhibitors 4, 13, 16**-**25**

The synthesis of **type I** inhibitor **3** and **type II** inhibitors **4, 13, 16**-**25** is presented in Supplementary Figure 1. Orthogonally protected piperidin-3-yl methanamine **14** was synthesized from nipecotic acid (**15**), as previously reported^10^. Compound **14** was then converted into amine di(2,2,2-trifluoroacetate) **9**^11^, which was further reacted with various sulfonyl chlorides to produce sulfonamides **3**, **13**, **16**-**21**. Sulfonamides **13**, **20**, and **21** were taken further. Nitro groups of sulfonamides **13** and **20** were reduced^11^ to primary amines **4** and **22**, respectively. Acetylation of the primary amino groups of **4** and **22** provided acetamides **22** and **24**, respectively. Amide **25** was prepared by reacting methyl ester **21** with MeNH_2_ in a sealed reaction tube.

**4.2. Synthesis of type III inhibitors 2, 37-41, and inhibitors 42 and 43**

The synthesis of **type III** inhibitors **2**, **37**-**41** and inhibitors **42** and **43** is delineated in Supplementary Figure 2. First, nipecotic acid (**15**) was converted into the secondary amine **10**^10^, which was then reacted with naphthalene-2-sulfonyl chloride, to produce sulfonamide **2**. Compound **2** was converted into its hydrochloride salt with a solution of HCl in 1,4-dioxane. To prepare derivatives of **type I** inhibitor **3** that have substituted benzyl rings instead of 2,3-dihydro-1*H*-inden, the amine **10** was first debenzylated to give amine **26**^11^. This crude amine was then reacted with various substituted benzaldehydes in the presence of sodium triacetoxyborohydride [NaBH(OAc)_3_]^13^, to produce tertiary amines **27**-**31**. After the removal of the *tert*-butyloxycarbonyl protecting groups with TFA in DCM^10^, the obtained crude amine 2,2,2-trifluoroacetates **32**-**36** were reacted with naphthalene-2-sulfonyl chloride to produce the target sulfonamides **37**-**41**. Compounds **42** and **43** were synthesized from secondary amine **10** using naphthalene-1-sulfonyl chloride and 7-methoxynaphtalene-2-sulfonyl chloride, respectively.

**4.3. Synthesis of type IV inhibitors 7, 8, 46**-**48 and inhibitor 49**

The *N*-alkyl group [-(CH_2_)_2_-OMe] of compound **2**, the most potent huBChE inhibitor in the series of benzyl derivatives of compound **3**, was removed and also replaced with methyl, ethyl, *n*-propyl, *n*-butyl, and -(CH_2_)_3_-OMe groups. The synthesis of this series of compounds is shown in Supplementary Figure 3. First, nipecotamide (**44**) was converted into the primary amine **45**^10^, which was then reacted with naphthalene-2-sulfonyl chloride to produce sulfonamide **7**. The sulfonamide nitrogen of this compound was then alkylated with various alkyl halides to produce sulfonamides **8** and **46**-**48**. The derivative of compound **2** with the -(CH_2_)_3_-OMe moiety, compound **5**, was prepared from secondary amine **11** and naphthalene-2-sulfonyl chloride. The secondary amine **11** was prepared from nipecotic acid (**15**), as previously reported^10^. Compound **11** was also reacted with 7-(cyanomethoxy)naphthalene-2-sulfonyl chloride to produce sulfonamide **49**.

**4.4. Synthesis of type IV inhibitors 64**-**69, 71 and 72**

This synthesis of a series of compound **7** derivatives is shown in Supplementary Figure 4. Nipectoamide (**44**) was first converted into orthogonally protected piperidin-3-ylmethanamine **50**^10^, which was then debenzylated to produce secondary amine **51**^14^. This compound was then either alkylated with various alkyl chlorides, to produce tertiary amines **52**-**55**, or reacted with 4-fluorobenzaldehyde and 4-formylbenzonitrile in the presence of sodium triacetoxyborohydride [NaBH(OAc)_3_]^13^, to produce tertiary amines **56** and **57**, respectively. After the removal of the *tert*-butyloxycarbonyl protecting groups from compounds **52**-**57**^10^, the obtained crude amine 2,2,2-trifluoroacetates **58**-**63** were reacted with naphthalene-2-sulfonyl chloride to produce sulfonamides **64**-**69**. Amine **51** was also converted into benzothiazole **70**^14^ which was reacted with naphthalene-2-sulfonyl chloride to yield sulfonamide **71**. Compound **72** was prepared from **68** by alkylating the sulfonamide nitrogen with methyl iodide.

**4.5. Synthesis of type V inhibitors 6, 75 and 84-86**

The synthesis of a series of 1,4-disubstituted piperidine derivatives of inhibitor **3** is presented in Supplementary Figure 5. Isonipecotic acid (**73**) was converted into secondary amines **74** and **12**^10^, which were further reacted with naphthalene-2-sulfonyl chloride to produce sulfonamides **75** and **6**, respectively. Carbamates **76** and **77** were synthesized from the corresponding amines **74** and **12**^10^, respectively, and then debenzylated with cyclohexene in the presence of Pearlman’s catalyst (palladium hydroxide on carbon)^11^, to produce crude secondary amines **78** and **79**. These crude amines were reacted with 1*H*-inden-2(3*H*)-one in the presence of sodium triacetoxyborohydride [NaBH(OAc)_3_]^11^, to provide tertiary amines **80** and **81**. After the removal of the *tert*-butyloxycarbonyl protecting groups^11^ from carbamates **80** and **81**, the obtained crude amine di(2,2,2-trifluoroacetates) **82** and **83** were reacted with naphthalene-2-sulfonyl chloride, to produce the final sulfonamides **84** and **85**. Compound **86**, which lacks the *N*-alkyl group on the sulfonamide nitrogen, was prepared from primary amine **87** and naphthalene-2-sulfonyl chloride. Primary amine **87** was prepared from isonipecotamide (**88**), as previously reported^10^.

**5. GENERAL CHEMISTRY METHODS**

^1^H-NMR and ^13^C-NMR were recorded at 400.130 MHz and 100.613 MHz, respectively, on a Bruker Avance III NMR spectrophotometer. The chemical shifts (δ) are reported in parts per million (ppm), and are referenced to the deuterated solvent used. The coupling constants (*J*) are reported in Hz, and the splitting patterns are indicated as: s (singlet), bs (broad singlet), d (doublet), dd (doublet of doublets), td (triplet of doublets), h (hextet), m (multiplet), t (triplet), bt (broad triplet), dt (doublet of triplets), tt (triplet of triplets), q (quartet), and qd (quartet of doublets). Optical rotations were measured on a Perkin-Elmer 241 MC polarimeter. The reported values for specific rotation are the mean values of 10 successive measurements using an integration time of 5 s. Infrared (IR) spectra were recorded on a Perkin-Elmer FT-IR System Spectrum BX. ATR IR spectra were recorded on a Thermo Nicolet Nexus 470 ESP FT-IR spectrometer. Mass spectra were recorded on a VG-Analytical AutoSpec Q Micromass mass spectrometer. Evaporation of the solvents was performed at reduced pressure. Reagents and solvents were purchased from Acros Organics, Alfa Aesar, Euriso-Top, Fluka, Merck, Sigma-Aldrich and TCI Europe, and were used without further purification, unless otherwise stated. Flash column chromatography was performed on silica gel 60 for column chromatography (particle size, 230-400 mesh). Analytical thin-layer chromatography was performed on Merck silica gel 60 F254 aluminum sheets (0.20 mm), with visualization using ultraviolet light and/or visualization reagents. Semi-preparative reversed-phase chiral HPLC (method A), analytical reversed-phase chiral HPLC (method B), and analytical reversed-phase HPLC (methods D, E, F, G, H) were performed on an Agilent 1100 LC modular system equipped with an autosampler, a quarternary pump system, a photodiode array detector, a thermostated column compartment, a fraction collector compartment, and a ChemStation data system. Analytical reversed-phase HPLC method C was performed on a Thermo Scientific Dionex Ultimate 3000 Binary Rapid Seperation LC System equipped with an autosampler, a binary pump system, a photodiode array detector, a thermostated column compartment, and a Chromeleon Chromatography Data System. The detector on both HPLC systems was set to 210, 254, and 280 nm. The column used for semi-preparative reversed-phase chiral HPLC method A was a Kromasil 5-CelluCoat RP column (250 × 10 mm). A guard cartridge was used with this column, as a Kromasil 5-CelluCoat RP (10-21.2 mm). The column used for analytical reversed-phase chiral HPLC method B was a Kromasil 3-CelluCoat RP column (150 × 4.6 mm). The column used for analytical reversed-phase HPLC methods C, D, E, F, G and H was a Zorbax Eclipse Plus C18 analytical column (150 × 4.6 mm, 5 µm; Agilent). An HPLC guard cartridge system was used, as a Security Guard Cartridge C18 CODS (octadecyl; 4.0 mm × 3.0 mm ID; Phenomenex). The HPLC columns were thermostated at 25 °C.

*Method A*: The sample solution of compound **2** (100 μL; 10 mg mL^–1^ in acetonitrile [MeCN]) was injected and eluted over 55 min at a flow rate of 5 mL min^–1^, using aqueous sodium borate buffer (20 mM, pH 9.00) containing 49% MeCN.

*Method B:* The sample solution (10 μL; 0.1 mg mL^–1^ in MeCN) was injected and eluted over 40 min at a flow rate of 0.8 mL min^–1^, using aqueous sodium borate buffer (20 mM, pH 9.00) containing 49% MeCN.

*Method C*: The sample solution (10 μL; 0.1 mg mL^–1^ in MeCN) was injected and eluted at a flow rate of 1 mL min^–1^, using a linear gradient of mobile phase A (70% aqueous phosphate buffer: 5 mM, pH 8.00; [v/v] in MeCN) and mobile phase B (30% aqueous phosphate buffer: 5 mM, pH 8.00; [v/v] in MeCN). The gradient for method C (for mobile phase B) was: 0-7 min, 0%-100%; 7-20 min, 100%.

*Method D*: The sample solution (10 μL; 0.1 mg mL^–1^ in MeCN) was injected and eluted at a flow rate of 1 mL min^–1^, using a linear gradient of mobile phase A (MeCN) and mobile phase B (aqueous phosphate buffer: 20 mM, pH 8.00). The gradient for method D (for mobile phase A) was: 0-15 min, 30%-70%; 15-20 min, 70%; 20-25 min, 70%-30%.

*Method E:* The sample solution (10 μL; 0.1 mg mL^–1^ in MeCN) was injected and eluted at a flow rate of 1 mL min^–1^, using a linear gradient of mobile phase A (MeCN) and mobile phase B (aqueous phosphate buffer: 20 mM, pH 8.00). The gradient for method E (for mobile phase A) was: 0-5 min, 50%-70%; 5-25 min, 70%; 25-30 min, 70%-50%.

*Method F*: The sample solution (10 μL; 0.1 mg mL^–1^ in MeCN) was injected and eluted at a flow rate of 1 mL min^–1^, using a linear gradient of mobile phase A (MeCN) and mobile phase B (aqueous phosphate buffer: 20 mM, pH 8.00). The gradient for method F (for mobile phase A) was: 0-5 min, 30%-70%; 5-20 min, 70%; 20-25 min, 70%-30%.

*Method G*: The sample solution (10 μL; 0.1 mg mL^–1^ in MeCN) was injected and eluted at a flow rate of 1 mL min^–1^, using a linear gradient of mobile phase A (0.1% trifluoroacetic acid [TFA]; [v/v] in MeCN) and mobile phase B (0.1% aqueous TFA [v/v]). The gradient for method G (for mobile phase A) was: 0-16 min, 10%-90%; 16-19 min, 90%; 19-20 min, 90%-10%.

*Method H*: The sample solution (10 μL; 0.1 mg mL^–1^ in water) was injected and eluted at a flow rate of 1 mL min^–1^, using a linear gradient of mobile phase A (MeCN) and mobile phase B (aqueous phosphate buffer: 20 mM, pH 8.00). The gradient for method H (for mobile phase A) was: 0-15 min, 30%-70%; 15-20 min, 70%; 20-25 min, 70%-30%.

**6. GENERAL SYNTHETIC PROCEDURES**

**General procedure for formation of sulfonamide bond (general procedure 1)**

The amine (1.0 equiv.) or 2,2,2-trifluoroacetate salt of amine (1.0 equiv.) was dissolved in DCM and cooled to 0 °C. The reaction mixture was stirred and trimethylamine (Et_3_N) (1.0 equiv. for amine and (1.0 + *n*) equiv. for 2,2,2-trifluoroacetate salt of amine (*n* being the molar quantity of 2,2,2-trifluoroacetate in the salt)) was added drop-wise. After 15 min, sulfonyl chloride (1.0 equiv.) was added, and the reaction mixture was allowed to warm to room temperature and then stirred for 24 h. The reaction mixture was transferred into a separating funnel, washed with water followed by saturated aqueous NaHCO_3_ solution, dried over anhydrous Na_2_SO_4_, and evaporated. The crude product was purified by flash column chromatography.

**General procedure for reduction of aromatic nitro group (general procedure 2)**

The aromatic nitro compound (1.0 equiv.) was dissolved in a mixture of THF/MeOH (1:1, v/v) and cooled to 0 °C. NiCl_2_×6H_2_O (4.0 equiv.) and NaBH_4_ (18.0 equiv.) were added, and the resulting suspension was stirred at 0 °C for 2 h, then allowed to warm up to room temperature, and stirred for an additional 16 h. The solvent was evaporated and DCM was added to the residue. The precipitated solid was filtered under suction, and washed with DCM. Combined filtrates were evaporated, and the crude product purified by flash column chromatography.

**General procedure for acetylation of aromatic amino group (general procedure 3)**

The primary aromatic amine (1.0 equiv.) was dissolved in pyridine at room temperature. Ac_2_O (10 equiv.) was added, and the resulting solution was stirred for 16 h. The reaction mixture was evaporated and the crude product purified by flash column chromatography.

**General procedure for reduction amination with aldehydes (general procedure 4)**

The secondary amine (1.0 equiv.) was dissolved in DCE at room temperature. The solution was stirred and agitated with a stream of argon for 15 min. NaBH(OAc)_3_ (1.875 equiv.), aldehyde (1.0 equiv.) and AcOH (1.0 equiv.) were added, and the resulting suspension was stirred under an atmosphere of argon for 24 h. The reaction mixture was opened to the air and quenched with saturated aqueous NaHCO_3_ solution. The mixture was transferred into a separating funnel, and DCM was added. The separating funnel was shaken vigorously, and the organic phase was separated, dried over anhydrous Na_2_SO_4_, and evaporated. The crude product was purified by flash column chromatography.

**General procedure for removal of Boc-protective group (general procedure 5)**

Carbamate (1.0 equiv.) was dissolved in DCM at room temperature. The solution was stirred and TFA (20.0 equiv.) was added drop-wise. After 24 h, the reaction mixture was evaporated. The residue was co-evaporated with Et_2_O. The crude product was used in the next step without further purification.

**General procedures for alkylation of sulfonamide nitrogen (general procedure 6) The s**ulfonamide **7** (1.0 equiv.) was dissolved in MeCN at room temperature under an atmosphere of argon. Cs_2_CO_3_ (1.5 equiv.), NaI (catalytic amount), and alkyl halide (6.0 equiv.) were added, and the resulting suspension was stirred at 60 °C from 1 h to 6 h. The reaction mixture was evaporated, and the residue suspended in DCM, transferred into a separating funnel, and washed with water followed by saturated aqueous NaHCO_3_ solution, dried over anhydrous Na_2_SO_4_, and evaporated. The crude product was purified by flash column chromatography.

**General procedure for alkylation of secondary amine (general procedure 7)**

The secondary amine (1.0 equiv.) was dissolved in anhydrous DMF at room temperature. K_2_CO_3_ (3.0 equiv.) was added, followed by alkyl chloride (1.5 equiv.), and the reaction mixture was stirred at room temperature for 20 h. The reaction mixture was evaporated. The residue was dissolved in DCM, transferred into a separating funnel, washed with saturated aqueous NaHCO_3_ solution, followed by saturated brine solution, dried over anhydrous Na_2_SO_4_, and evaporated. The crude product was purified by flash column chromatography.

**General procedure for removal of benzyl protective group (general procedure 8)**

The benzyl amine (1.0 equiv.) was dissolved in MeOH at room temperature. The solution was stirred and agitated with a stream of argon for 30 min. Pd(OH)_2_ on carbon (20 wt.%) (20% mass of benzyl amine) was added, followed by cyclohexene (10.0 equiv.). The resulting suspension was refluxed under an atmosphere of argon for 20 h, then filtered under suction through a pad of Celite, and evaporated. The crude product was used in the next step without further purification.

**General procedure for reductive amination with 1*H*-inden-2(3*H*)-one (general procedure 9)**

The secondary amine (1.0 equiv.) was dissolved in DCE at room temperature. The solution was stirred and agitated with a stream of argon for 15 min. NaBH(OAc)_3_ (1.875 equiv.), 1*H*-inden-2(3*H*)-one (1.0 equiv.), and AcOH (1.0 equiv.) were added, and the resulting suspension was stirred under an atmosphere of argon for 24 h. The reaction mixture was opened to the air and quenched with saturated aqueous NaHCO_3_ solution. The mixture was transferred into a separating funnel, and DCM was added. The separating funnel was shaken vigorously, and the organic phase was separated, dried over anhydrous Na_2_SO_4_, and evaporated. The crude product was purified by flash column chromatography.

**7. SYNTHESIS AND CHARACTERIZATION OF COMPOUNDS**

***Synthesis of (±)-N-((1-(2,3-dihydro-1H-inden-2-yl)piperidin-3-yl)methyl)-N-(2-methoxyethyl)naphthalene-2-sulfonamide (3)***

Synthesized from (±)-*N*-((1-(2,3-dihydro-1*H*-inden-2-yl)piperidin-3-yl)methyl)-2-methoxyethanamine di(2,2,2-trifluoroacetate) ^11^ (**9**) and naphthalene-2-sulfonyl chloride via general procedure 1. Purified by flash column chromatography using DCM/MeOH (20:1, v/v) as the eluent to produce 0.220 g of **3** as a slightly golden oil (93% yield). *R*_f_ = 0.51 (DCM/MeOH, 10:1, v/v). IR (ATR): 2928, 1687, 1589, 1456, 1334, 1154, 1115, 1072, 991, 858, 816, 743, 649, 614 cm^-1^. ^1^H-NMR (400.130 MHz, CDCl_3_): δ = 0.92–1.03 (1 H, m), 1.58–1.77 (4 H, m), 1.97–2.06 (2 H, m), 2.82–3.06 (6 H, m), 3.09–3.21 (3 H, m), 3.24 (3 H, s), 3.34–3.41 (2 H, m), 3.49–3.55 (2 H, m), 7.11–7.17 (4 H, m), 7.59–7.66 (2 H, m), 7.80 (1 H, dd, *J_1_* = 8.7 Hz, *J_2_* = 1.9 Hz), 7.88–7.98 (3 H, m), 8.40 (1 H, d, *J* = 1.4 Hz). ^13^C-NMR (100.613 MHz, CDCl_3_): δ = 24.70, 28.41, 34.73, 36.72, 36.95, 47.81, 52.06, 53.20, 55.92, 58.58, 67.07, 70.96, 122.42, 124.16, 124.21, 126.16, 127.34, 127.69, 128.24, 128.50, 128.99, 129.08, 131.97, 134.49, 136.39, 141.38. HRMS (ESI+): *m/z* calcd for C_28_H_35_N_2_O_3_S [M+H]^+^ 479.2368; found 479.2354. HPLC purity, 98% at 254.16 nm (method C, *t*_R_ = 13.88 min).

***Synthesis of (±)-N-((1-(2,3-dihydro-1H-inden-2-yl)piperidin-3-yl)methyl)-N-(2-methoxyethyl)-3-nitrobenzenesulfonamide*** ***(13)***

Synthesized from (±)-*N*-((1-(2,3-dihydro-1*H*-inden-2-yl)piperidin-3-yl)methyl)-2-methoxyethanamine di(2,2,2-trifluoroacetate)^11^ (**9**) and 3-nitrobenzene-1-sulfonyl chloride via general procedure 1. Purified by flash column chromatography using DCM/MeOH (20:1, v/v) as the eluent to produce 0.145 g of **13** as an orange oil (40% yield). *R*_f_ = 0.27 (DCM/MeOH, 20:1, v/v). IR (ATR): 2932, 2847, 2812, 1687, 1531, 1459, 1349, 1274, 1198, 1161, 1118, 1070, 993, 928, 878, 766, 745, 718 cm^-1^. ^1^H-NMR (400.130 MHz, CDCl_3_): δ = 0.94–1.05 (1 H, m), 1.54–1.64 (1 H, m), 1.71–1.79 (3 H, m), 1.95–2.09 (2 H, m), 2.82–2.97 (4 H, m), 3.01–3.09 (2 H, m), 3.12–3.16 (2 H, m), 3.17 (3 H, s), 3.32-3.45 (4 H, m), 3.52–3.61 (1 H, m), 7.11–7.18 (4 H, m), 7.69 (1 H, t, *J =* 8.2 Hz), 8.15 (1 H, ddd, *J_1_ =* 7.8 Hz, *J_2_* = 1.8 Hz, *J_3_* = 1.0 Hz), 8.37 (1 H, ddd, *J_1_ =* 7.8 Hz, *J_2_* = 2.2 Hz, *J_3_* = 1.2 Hz), 8.67 (1 H, t, *J =* 1.8 Hz). ^13^C-NMR (100.613 MHz, CDCl_3_): δ = 24.67, 28.36, 34.36, 36.79, 36.99, 47.36, 52.07, 52.10, 55.75, 58.60, 67.09, 69.91, 122.33, 124.24, 124.27, 126.26, 126.31, 126.64, 130.05, 132.76, 141.36, 141.39, 142.19, 148.04. HRMS (ESI+): *m/z* calcd for C_24_H_32_N_3_O_5_S [M+H]^+^ 474.2063; found 474.2055. HPLC purity, 95% at 254.16 nm (method C, *t*_R_ = 10.68 min).

***Synthesis of (±)-3-amino-N-((1-(2,3-dihydro-1H-inden-2-yl)piperidin-3-yl)methyl)-N-(2-methoxyethyl)benzenesulfonamide*** ***(4)***

Synthesized from (±)-*N*-((1-(2,3-dihydro-1*H*-inden-2-yl)piperidin-3-yl)methyl)-*N*-(2-methoxyethyl)-3-nitrobenzenesulfonamide (**13**) via general procedure 2. Purified by flash column chromatography using DCM/MeOH (20:1, v/v) as the eluent to produce 0.064 g of **4** as a pale brown solid (57% yield). *R*_f_ = 0.23 (DCM/MeOH, 20:1, v/v). IR (ATR): 3458, 3375, 2931, 2849, 2811, 1627, 1597, 1484, 1450, 1313, 1276, 1151, 1114, 1082, 990, 923, 888, 865, 734 cm^-1^. ^1^H-NMR (400.130 MHz, CDCl_3_): δ = 0.93 (1 H, qd, *J_1_ =* 11.8 Hz, *J_2_* = 3.1 Hz), 1.53–1.63 (1 H, m), 1.66–1.74 (3 H, m), 1.91–1.99 (2 H, m), 2.81–2.97 (4 H, m), 3.00–3.15 (5 H, m), 3.24–3.28 (2 H, m), 3.27 (3 H, s), 3.48–3.52 (2 H, m), 4.00 (2 H, bs), 6.77 (1 H, ddd, *J_1_ =* 8.0 Hz, *J_2_* = 2.3 Hz, *J_3_* = 1.0 Hz,), 7.08 (1 H, t, *J =* 1.8 Hz), 7.10–7.17 (5 H, m), 7.22 (1 H, t, *J =* 7.8 Hz). ^13^C-NMR (100.163 MHz, CDCl_3_): δ = 24.73, 28.42, 34.82, 36.80, 36.92, 48.06, 52.18, 53.51, 55.89, 58.63, 67.12, 71.18, 112.73, 116.38, 118.49, 124.21, 124.23, 126.18, 126.21, 129.75, 139.91, 141.45, 147.19. HRMS (ESI+): *m/z* calcd for C_24_H_34_N_3_O_3_S [M+H]^+^ 444.2321; found 444.2312. HPLC purity, 99% at 254.16 nm (method C, *t*_R_ = 8.53 min).

***Synthesis of (±)-N-((1-(2,3-dihydro-1H-inden-2-yl)piperidin-3-yl)methyl)-N-(2-methoxyethyl)-4-methylbenzenesulfonamide (17)***

Synthesized from (±)-*N*-((1-(2,3-dihydro-1*H*-inden-2-yl)piperidin-3-yl)methyl)-2-methoxyethanamine di(2,2,2-trifluoroacetate)^11^ (**9**) and 4-methylbenzene-1-sulfonyl chloride via general procedure 1. Purified by flash column chromatography using DCM/MeOH (20:1, v/v) as the eluent to produce 0.119 g of **17** as a slightly golden oil (92% yield). *R*_f_ = 0.50 (DCM/MeOH, 10:1, v/v). IR (ATR): 2928, 1688, 1598, 1451, 1337, 1156, 1114, 1089, 991, 814, 733, 652 cm^-1^. ^1^H-NMR (400.130 MHz, CDCl_3_): δ = 0.92–1.01 (1 H, m), 1.62–2.04 (7 H, m), 2.41 (3 H, s), 2.86–3.12 (8 H, m), 3.23–3.29 (5 H, m), 3.47–3.55 (2 H, m), 7.12–7.20 (4 H, m), 7.30 (2 H, d, *J* = 8.5 Hz), 7.70 (2 H, d, *J* = 8.3 Hz). ^13^C-NMR (100.613 MHz, CDCl_3_): δ = 21.34, 24.65, 28.39, 34.70, 36.75, 36.96, 47.95, 52.11, 53.33, 55.91, 58.60, 67.11, 71.07, 124.21, 126.22, 127.07, 129.46, 136.35, 141.38, 143.06. HRMS (ESI+): *m/z* calcd for C_24_H_34_N_2_O_3_S [M+H]^+^ 443.2368; found 443.2358. HPLC purity, 98% at 254.16 nm (method C, *t*_R_ = 12.01 min).

***Synthesis of (±)-N-((1-(2,3-dihydro-1H-inden-2-yl)piperidin-3-yl)methyl)-N-(2-methoxyethyl)-4-nitrobenzenesulfonamide (20)***

Synthesized from (±)-*N*-((1-(2,3-dihydro-1*H*-inden-2-yl)piperidin-3-yl)methyl)-2-methoxyethanamine di(2,2,2-trifluoroacetate)^11^ (**9**) and 4-nitrobenzene-1-sulfonyl chloride via general procedure 1. Purified by flash column chromatography using DCM/MeOH (20:1, v/v) as the eluent to produce 0.188 g of **20** as a pale orange oil (68% yield). *R*_f_ = 0.32 (DCM/MeOH, 20:1, v/v). IR (ATR): 2932, 2847, 2806, 1687, 1605, 1528, 1477, 1459, 1400, 1347, 1310, 1198, 1160, 1114, 1088, 1013, 993, 927, 889, 854, 742, 714 cm^-1^. ^1^H-NMR (400.130 MHz, CDCl_3_): δ = 0.93–1.05 (1 H, m), 1.54–1.64 (1 H, m), 1.73–1.81 (3 H, m), 1.94–2.08 (2 H, m), 2.83–2.97 (4 H, m), 3.01–3.10 (2 H, m), 3.12–3.15 (2 H, m), 3.17 (3 H, s), 3.37–3.48 (4 H, m), 3.51–3.62 (1 H, m), 7.11–7.18 (4 H, m), 8.00 (2 H, dt, *J_1_ =* 8.8 Hz, *J_2_* = 2.2 Hz), 8.33 (2 H, dt, *J_1_ =* 8.8 Hz, *J_2_* = 2.2 Hz). ^13^C-NMR (100.613 MHz, CDCl_3_): δ = 24.69, 28.38, 34.47, 36.86, 37.07, 47.52, 52.10, 52.44, 55.77, 58.59, 67.11, 70.11, 124.02, 124.27, 124.30, 126.32, 126.34, 128.36, 141.40, 141.44, 145.76, 149.71. HRMS (ESI+): *m/z* calcd for C_24_H_32_N_3_O_5_S [M+H]^+^ 474.2063; found 474.2069. HPLC purity, 95% at 254.16 nm (method C, *t*_R_ = 10.73 min).

***Synthesis of (±)-4-amino-N-((1-(2,3-dihydro-1H-inden-2-yl)piperidin-3-yl)methyl)-N-(2-methoxyethyl)benzenesulfonamide (22)***

Synthesized from (±)-*N*-((1-(2,3-dihydro-1*H*-inden-2-yl)piperidin-3-yl)methyl)-*N*-(2-methoxyethyl)-4-nitrobenzenesulfonamide (**20**) via general procedure 2. Purified by flash column chromatography using DCM/MeOH (20:1, v/v) as the eluent to produce 0.103 g of **22** as a yellow oil (81% yield). *R*_f_ = 0.12 (DCM/MeOH, 20:1, v/v). IR (ATR): 3473, 3374, 2931, 2851, 2809, 1629, 1595, 1503, 1456, 1337, 1314, 1145, 1115, 1089, 991, 830, 736, 711 cm^-1^. ^1^H-NMR (400.130 MHz, CDCl_3_): δ = 0.92 (1 H, qd, *J_1_ =* 11.6 Hz, *J*_2_ = 3.4 Hz), 1.52–1.62 (1 H, m), 1.65–1.73 (3 H, m), 1.89–1.99 (2 H, m), 2.82–3.18 (9 H, m), 3.20–3.25 (2 H, m), 3.27 (3 H, s), 3.51 (2 H, t, *J =* 6.4 Hz), 4.20 (2 H, bs), 6.62 (2 H, dt, *J_1_ =* 9.2 Hz, *J*_2_ = 2.4 Hz), 7.10–7.18 (4 H, m), 8.54 (2 H, dt, *J_1_ =* 9.2 Hz, *J_2_* = 2.3 Hz). ^13^C-NMR (100.613 MHz, CDCl_3_): δ = 24.78, 28.51, 34.88, 36.82, 36.96, 48.03, 52.21, 53.47, 55.98, 58.66, 67.15, 71.26, 113.80, 124.23, 124.27, 126.21, 127.03, 129.14, 141.49, 150.51. HRMS (ESI+): *m/z* calcd for C_24_H_34_N_3_O_3_S [M+H]^+^ 444.2321; found 444.2329. HPLC purity, 96% at 254.16 nm (method C, *t*_R_ = 8.16 min).

***Synthesis of (±)-N-(4-(N-((1-(2,3-dihydro-1H-inden-2-yl)piperidin-3-yl)methyl)-N-(2-methoxyethyl)sulfamoyl)phenyl)acetamide*** *(****23)***

Synthesized from (±)-4-amino-*N*-((1-(2,3-dihydro-1*H*-inden-2-yl)piperidin-3-yl)methyl)-*N*-(2-methoxyethyl)benzenesulfonamide (**22**) via general procedure 3. Purified by flash column chromatography using DCM/MeOH (9:1, v/v) as the eluent to produce 0.078 g of **23** as a yellow oil (98% yield). *R*_f_ = 0.33 (DCM/MeOH, 9:1, v/v). IR (ATR): 3256, 3177, 2934, 1694, 1590, 1534, 1496, 1448, 1401, 1369, 1313, 1261, 1152, 1114, 1090, 1046, 1008, 842, 743 cm^-1^. ^1^H-NMR (400.130 MHz, CDCl_3_): δ = 0.93 (1 H, qd, *J_1_ =* 11.6 Hz, *J*_2_ = 3.4 Hz), 1.54–1.63 (1 H, m), 1.67–1.74 (3 H, m), 1.91–2.01 (2 H, m), 2.16 (3 H, s), 2.82–3.17 (9 H, m), 3.24–3.27 (2 H, m), 3.25 (3 H, s), 3.48 (2 H, t, *J =* 6.4 Hz), 7.10–7.16 (4 H, m), 7.64–7.71 (4 H, m), 8.20 (1 H, bs). ^13^C-NMR (100.613 MHz, CDCl_3_): δ = 24.53, 24.67, 28.43, 34.75, 36.78, 36.95, 48.07, 52.18, 53.33, 55.89, 58.70, 67.15, 71.01, 119.24, 124.28, 124.30, 126.32, 128.25, 133.74, 141.39, 142.03, 169.02. HRMS (ESI+): *m/z* calcd for C_26_H_36_N_3_O_4_S [M+H]^+^ 486.2427; found 486.2431. HPLC purity, 97% at 254.16 nm (method C, *t*_R_ = 7.71 min).

***Synthesis of (±)-N-((1-(2,3-dihydro-1H-inden-2-yl)piperidin-3-yl)methyl)-N-(2-methoxyethyl)benzenesulfonamide (16)***

Synthesized from (±)-*N*-((1-(2,3-dihydro-1*H*-inden-2-yl)piperidin-3-yl)methyl)-2-methoxyethanamine di(2,2,2-trifluoroacetate)^11^ (**9**) and benzenesulfonyl chloride via general procedure 1. Purified by flash column chromatography using DCM/MeOH (20:1, v/v) as the eluent to produce 0.117 g of **16** as a slightly golden oil (92% yield). *R*_f_ = 0.50 (DCM/MeOH, 10:1, v/v). IR (ATR) = 2929, 1687, 1446, 1335, 1156, 1115, 1090, 991, 925, 740, 690 cm^-1^. ^1^H-NMR (400.130 MHz, CDCl_3_): δ = 0.91–1.01 (1 H, m), 1.63–1.76 (4 H, m), 1.99–2.04 (2 H, m), 2.91–3.18 (9 H, m), 3.25–3.34 (5 H, m), 3.45–3.54 (2 H, m), 7.12–7.19 (4 H, m), 7.48–7.58 (3 H, m), 7.81–7.84 (2 H, m). ^13^C-NMR (100.613 MHz, CDCl_3_): δ = 24.65, 28.36, 34.65, 36.76, 36.94, 47.82, 52.10, 53.19, 55.85, 58.56, 67.07, 70.91, 124.18, 124.20, 126.19, 126.20, 126.98, 128.83, 132.29, 139.41, 141.28. HRMS (ESI+): *m/z* calcd for C_24_H_33_N_2_O_3_S [M+H]^+^ 429.2212; found 429.2209. HPLC purity, 96% at 254.16 nm (method C, *t*_R_ = 10.54 min).

***Synthesis of (±)-methyl 2-(N-((1-(2,3-dihydro-1H-inden-2-yl)piperidin-3-yl)methyl)-N-(2-methoxyethyl)sulfamoyl)benzoate (21)***

Synthesized from (±)-*N*-((1-(2,3-dihydro-1*H*-inden-2-yl)piperidin-3-yl)methyl)-2-methoxyethanamine di(2,2,2-trifluoroacetate)^11^ (**9**) and methyl 2-(chlorosulfonyl)benzoate via general procedure 1. Purified by flash column chromatography using DCM then DCM/MeOH (20:1, v/v) as the eluent to produce 0.154 g of **21** as a yellow oil (82% yield). *R*_f_ = 0.17 (DCM/MeOH, 20:1, v/v). IR (KBr): 2930, 1735, 1637, 1438, 1341, 1296, 1260, 1162, 1116, 1061, 889, 828, 746 cm^-1^. ^1^H-NMR (400.130 MHz, CDCl_3_): δ = 0.92 (1 H, qd, *J_1_ =* 12.0 Hz, *J_2_* = 3.2 Hz), 1.54–1.63 (2 H, m), 1.69–1.74 (2 H, m), 1.92–2.00 (2 H, m), 2.78–2.86 (2 H, m), 2.91–2.95 (2 H, m), 2.99–3.05 (2 H, m), 3.08–3.14 (1 H, m), 3.23–3.25 (2 H, m), 3.25 (3 H, s), 3.42–3.52 (4 H, m), 3.95 (3 H, s), 7.12–7.18 (4 H, m), 7.44–7.49 (1 H, m), 7.52–7.57 (2 H, m), 7.85–7.90 (1 H, m). ^13^C-NMR (100.613 MHz, CDCl_3_): δ = 24.78, 28.33, 34.35, 36.78, 36.81, 46.66, 52.15, 52.26, 53.03, 55.62, 58.60, 67.06, 70.76, 124.20, 124.24, 126.19, 128.29, 128.46, 130.08, 131.98, 132.90, 137.86, 141.45, 168.27. HRMS (ESI+): *m/z* calcd for C_26_H_35_N_2_O_5_S [M+H]^+^ 487.2267; found 487.2269. HPLC purity, 99% at 254.16 nm (method C, *t*_R_ = 9.64 min).

***Synthesis of (±)-N-((1-(2,3-dihydro-1H-inden-2-yl)piperidin-3-yl)methyl)-4-methoxy-N-(2-methoxyethyl)benzenesulfonamide*** ***(19)***

Synthesized from (±)-*N*-((1-(2,3-dihydro-1*H*-inden-2-yl)piperidin-3-yl)methyl)-2-methoxyethanamine di(2,2,2-trifluoroacetate)^11^ (**9**) and 4-methoxybenzene-1-sulfonyl chloride via general procedure 1. Purified by flash column chromatography using DCM/MeOH (50:1, v/v) then DCM/MeOH (20:1, v/v) as the eluent to produce 0.074 g of **19** as a yellow transparent oil (72% yield). *R*_f_ = 0.30 (DCM/MeOH, 20:1, v/v). IR (KBr): 2932, 2845, 1638, 1618, 1498, 1459, 1384, 1340, 1304, 1260, 1158, 1115, 1093, 1026, 926, 835, 806, 746 cm^-1^. ^1^H-NMR (400.130 MHz, MeOD): δ = 0.94 (1 H, qd, *J =* 12.4, 3.2 Hz), 1.57–1.59 (1 H, m), 1.69–1.76 (3 H, m), 1.92–2.01 (2 H, m), 2.83–3.17 (9 H, m), 3.25–3.26 (2 H, m), 3.27 (3 H, s), 3.49–3.52 (2 H, m), 3.85 (3 H, s), 6.97 (2 H, dt, *J_1_ =* 9.2 Hz, *J_2_* = 2.4 Hz), 7.12–7.19 (4 H, m), 7.75 (2 H, dt, *J_1_ =* 9.0 Hz, *J_2_* = 2.6 Hz). ^13^C-NMR (100.613 MHz, CDCl_3_): δ = 24.80, 28.52, 34.86, 36.89, 37.13, 48.00, 52.20, 53.39, 55.51, 56.07, 58.72, 67.21, 71.15, 114.04, 124.27, 124.30, 126.27, 129.22, 131.12, 141.51, 162.64. HRMS (ESI+): *m/z* calcd for C_25_H_35_N_2_O_4_S [M+H]^+^ 459.2318; found 459.2307. HPLC purity, 99% at 254.16 nm (method C, *t*_R_ = 10.53 min).

***Synthesis of (±)-N-((1-(2,3-dihydro-1H-inden-2-yl)piperidin-3-yl)methyl)-4-fluoro-N-(2-methoxyethyl)benzenesulfonamide (18)***

Synthesized from (±)-*N*-((1-(2,3-dihydro-1*H*-inden-2-yl)piperidin-3-yl)methyl)-2-methoxyethanamine di(2,2,2-trifluoroacetate)^11^ (**9**) and 4-fluorobenzene-1-sulfonyl chloride via general procedure 1. Purified by flash column chromatography using DCM/MeOH (20:1, v/v) as the eluent to produce 0.044 g of **18** as a pale brown solid (59% yield). *R*_f_ = 0.21 (DCM/MeOH, 9:1, v/v). IR (KBr): 2932, 2852, 2912, 1688, 1591, 1492, 1459, 1341, 1292, 1233, 1200, 1152, 1116, 1088, 1026, 1011, 993, 838, 820, 742 cm^-1^. ^1^H-NMR (400.130 MHz, CDCl_3_): δ = 0.95 (1 H, qd, *J_1_ =* 12.0 Hz, *J*_2_ = 3.0 Hz), 1.56–1.65 (1 H, m), 1.71–1.75 (3 H, m), 1.92–2.02 (2 H, m), 2.83–3.00 (4 H, m), 3.02–3.10 (4 H, m), 3.12–3.19 (1 H, m), 3.24 (3 H, s), 3.30 (2 H, t, *J =* 6.0 Hz), 3.48 (2 H, t, *J =* 6.4 Hz), 7.11–7.19 (6 H, m), 7.81–7.86 (2 H, m). ^13^C-NMR (100.613 MHz, CDCl_3_): δ = 24.79, 28.50, 34.76, 36.92, 37.14, 47.83, 52.19, 53.10, 55.97, 58.69, 67.18, 70.80, 116.08 (d, *^2^J =* 22.2 Hz), 124.28, 124.31, 126.30, 129.79 (d, *^3^J =* 9.1 Hz), 135.75 (d, *^4^J =* 2.9 Hz), 141.51, 164.85 (d, *^1^J =* 252.9 Hz). HRMS (ESI+): *m/z* calcd for C_24_H_32_N_2_O_3_SF [M+H]^+^ 447.2118; found 447.2110. HPLC purity, 98% at 254.16 nm (method C, *t*_R_ = 10.90 min).

***Synthesis of (±)-2-(N-((1-(2,3-dihydro-1H-inden-2-yl)piperidin-3-yl)methyl)-N-(2-methoxyethyl)sulfamoyl)-N-methylbenzamide*** ***(25)***

To a 10-mL glass sealed tube equipped with a stirring bar, **21** (0.047 g, 0.097 mmol, 1.0 equiv.) and CH_3_NH_2_ (2.5 mL, 8.03 M in EtOH) were added at room temperature. The reaction tube was sealed with a cap and the reaction mixture was stirred at 80 °C. After 10 days, the reaction mixture was transferred to a 50 mL round-bottomed flask and evaporated. The residue was purified by flash column chromatography using DCM/MeOH (20:1, v/v) then DCM/MeOH (9:1, v/v) as the eluent, to produce 0.012 g of amide **25** as an orange-brown solid (25% yield). *R*_f_ = 0.39 (DCM/MeOH, 9:1, v/v). IR (KBr): 3309, 2927, 2850, 1656, 1593, 1545, 1459, 1410, 1333, 1260, 1157, 1115, 1069, 992, 925, 745, 705 cm^-1^. ^1^H-NMR (400.130 MHz, CDCl_3_): δ = 0.95 (1 H, qd, *J_1_ =* 11.6 Hz, *J_2_* = 3.6 Hz), 1.56–1.65 (1 H, m), 1.70–1.76 (3 H, m), 1.95–2.02 (2 H, m), 2.83–2.96 (4 H, m), 3.01 (3 H, d, *J =* 5.2 Hz), 3.00–3.06 (2 H, m), 3.12–3.17 (1 H, m), 3.19 (3 H, s), 3.22 (2 H, t, *J =* 7.6 Hz), 3.39–3.46 (4 H, m), 6.40 (1 H, q, *J =* 4.6 Hz), 7.11–7.18 (4 H, m), 7.48–7.57 (3 H, m), 7.90–7.92 (1 H, m). ^13^C-NMR (100.613 MHz, CDCl_3_): δ = 24.53, 27.13, 28.22, 34.04, 36.60, 36.81, 46.53, 51.85, 52.02, 55.68, 58.62, 67.18, 70.29, 124.33, 124.39, 126.42, 128.95, 129.52, 129.57, 132.49, 136.49, 137.19, 141.27, 141.35, 168.62. HRMS (ESI+): *m/z* calcd for C_26_H_36_N_3_O_4_S [M+H]^+^ 486.2427; found 486.2438. HPLC purity, 95% at 254.16 nm (method C, *t*_R_ = 7.21 min).

***Synthesis of (±)-N-(3-(N-((1-(2,3-dihydro-1H-inden-2-yl)piperidin-3-yl)methyl)-N-(2-methoxyethyl)sulfamoyl)phenyl)acetamide*** ***(24)***

Synthesized from (±)-3-amino-*N*-((1-(2,3-dihydro-1*H*-inden-2-yl)piperidin-3-yl)methyl)-*N*-(2-methoxyethyl)benzenesulfonamide (**4**) via general procedure 3. Purified by flash column chromatography using DCM/MeOH (20:1, v/v) as the eluent to produce 0.056 g of **24** as a yellow oil (96% yield). *R*_f_ = 0.35 (DCM/MeOH, 9:1, v/v). IR (ATR): 3307, 3067, 2931, 2852, 2810, 1674, 1592, 1541, 1477, 1419, 1369, 1316, 1257, 1151, 1115, 992, 921, 889, 794, 741 cm^-1^. ^1^H-NMR (400.130 MHz, CDCl_3_): δ = 0.89–0.99 (1 H, m), 1.57–1.66 (1 H, m), 1.71–1.79 (3 H, m), 2.01–2.08 (2 H, m), 2.15 (3 H, s), 2.86–3.11 (9 H, m), 3.23 (3 H, s), 3.27 (2 H, t, *J =* 5.8 Hz), 3.43–3.50 (2 H, m), 7.10–7.15 (4 H, m), 7.42 (1 H, t, *J =* 7.8 Hz), 6.77 (1 H, dt, *J_1_ =* 8.0 Hz, *J_2_* = 1.6 Hz), 7.87 (1 H, s), 8.01 (1 H, d, *J =* 8.0 Hz), 8.54 (1 H, bs). ^13^C-NMR (100.613 MHz, CDCl_3_): δ = 24.35, 24.46, 28.23, 34.84, 36.65, 36.77, 48.33, 52.13, 53.30, 55.75, 58.69, 67.11, 71.04, 117.78, 122.07, 123.67, 124.27, 124.30, 126.42, 126.44, 129.70, 139.23, 139.69, 141.10, 141.13, 169.10. HRMS (ESI+): *m/z* calcd for C_26_H_36_N_3_O_4_S [M+H]^+^ 486.2427; found 486.2432. HPLC purity, 99% at 254.16 nm (method C, *t*_R_ = 7.95 min).

***Synthesis of (±)-N-((1-benzylpiperidin-3-yl)methyl)-N-(2-methoxyethyl)naphthalene-1-sulfonamide*** ***(42)***

Synthesized from (±)-*N*-((1-benzylpiperidin-3-yl)methyl)-2-methoxyethanamine^10^ (**10**) and naphthalene-1-sulfonyl chloride via general procedure 1. Purified by flash column chromatography using DCM/MeOH (20:1, v/v) as the eluent to produce 0.132 g of **42** as an orange oil (79% yield). *R*_f_ = 0.32 (DCM/MeOH, 20:1, v/v). IR (ATR): 3058, 3021, 2931, 2806, 2761, 1507, 1453, 1322, 1264, 1200, 1156, 1126, 1071, 1027, 980, 921, 883, 829, 803, 771, 739 cm^-1^. ^1^H-NMR (400.130 MHz, CDCl_3_): δ = 0.84 (1 H, qd, *J_1_ =* 11.8 Hz, *J_2_* = 3.8 Hz), 1.38–1.63 (4 H, m), 1.85 (1 H, t, *J =* 10.2 Hz), 1.90–1.97 (1 H, m), 2.59–2.66 (2 H, m), 3.15–3.20 (1 H, m), 3.17 (3 H, s), 3.25–3.36 (3 H, m), 3.39–3.42 (2 H, m), 3.46–3.50 (2 H, m), 7.21–7.31 (5 H, m), 7.50 (1 H, dd, *J_1_* = 8.2 Hz, *J_2_* = 7.4 Hz), 7.56 (1 H, ddd, *J_1_* = 8.0 Hz, *J_2_* = 6.8 Hz, *J_3_* = 1.2 Hz), 7.62 (1 H, ddd, *J_1_* = 8.6 Hz, *J_2_* = 6.4 Hz, *J_3_* = 1.6 Hz), 7.90 (1 H, dd, *J_1_* = 8.0 Hz, *J_2_* = 1.2 Hz), 8.03 (1 H, d, *J* = 8.4 Hz), 8.21 (1 H, dd, *J_1_* = 7.2 Hz, *J_2_* = 1.2 Hz), 8.65 (1 H, dd, *J_1_* = 8.2 Hz, *J_2_* = 0.6 Hz). ^13^C-NMR (100.613 MHz, CDCl_3_): δ = 24.39, 28.04, 34.25, 46.36, 52.00, 53.51, 57.56, 58.52, 63.13, 70.66, 123.91, 124.94, 126.65, 126.72, 127.83, 127.95, 128.61, 128.68, 128.92, 129.32, 133.91, 134.17, 134.93, 138.18. HRMS (ESI+): *m/z* calcd for C_26_H_33_N_2_O_3_S [M+H]^+^ 453.2212; found 453.2214. HPLC purity, 98% at 254.16 nm (method C, *t*_R_ = 13.58 min).

***Synthesis of (±)-N-((1-benzylpiperidin-3-yl)methyl)-7-methoxy-N-(2-methoxyethyl)naphthalene-2-sulfonamide*** ***(43)***

Synthesized from (±)-*N*-((1-benzylpiperidin-3-yl)methyl)-2-methoxyethanamine^10^ (**10**) and 7-methoxynaphthalene-2-sulfonyl chloride via general procedure 1. Purified by flash column chromatography using DCM/MeOH (20:1, v/v) as the eluent to produce 0.124 g of **43** as an orange oil (92% yield). *R*_f_ = 0.29 (DCM/MeOH, 20:1, v/v). IR (ATR): 2932, 2803, 1626, 1596, 1508, 1454, 1439, 1392, 1336, 1254, 1216, 1155, 1123, 1074, 1027, 987, 954, 916, 885, 842, 744, 717 cm^-1^. ^1^H-NMR (400.130 MHz, CDCl_3_): δ = 0.97–1.06 (1 H, m), 1.50–1.60 (1 H, m), 1.65–1.80 (3 H, m), 1.94–2.04 (2 H, m), 2.71–2.77 (1 H, m), 2.81–2.87 (1 H, m), 3.06–3.18 (2 H, m), 3.24 (3 H, s), 3.32 (2 H, t, *J =* 6.0 Hz), 3.46–3.52 (4 H, m), 3.95 (3 H, s), 7.24–7.32 (7 H, m), 7.64 (1 H, dd, *J_1_* = 8.6 Hz, *J_2_* = 2.2 Hz), 7.79 (1 H, d, *J* = 8.8 Hz), 7.86 (1 H, d, *J* = 8.8 Hz), 8.29 (1 H, d, *J* = 1.6 Hz). ^13^C-NMR (100.613 MHz, CDCl_3_): δ = 24.49, 28.16, 34.79, 48.07, 53.27, 53.83, 55.29, 57.55, 58.58, 63.28, 71.11, 106.56, 120.23, 121.50, 126.77, 126.91, 127.98, 128.76, 129.01, 129.14, 130.06, 133.45, 136.69, 138.18, 158.54. HRMS (ESI+): *m/z* calcd for C_27_H_35_N_2_O_4_S [M+H]^+^ 483.2318; found 483.2323. HPLC purity, 98% at 254.16 nm (method C, *t*_R_ = 14.45 min).

***Synthesis of (±)-N-((1-benzylpiperidin-3-yl)methyl)-7-(cyanomethoxy)-N-(3-methoxypropyl)naphthalene-2-sulfonamide*** ***(49)***

Synthesized from (±)-*N*-((1-benzylpiperidin-3-yl)methyl)-3-methoxypropan-1-amine^10^ (**11**) and 7-(cyanomethoxy)naphthalene-2-sulfonyl chloride via general procedure 1. Purified by flash column chromatography using DCM/MeOH (30:1, v/v) as the eluent to produce 0.125 g of **49** as a yellow oil (75% yield). *R*_f_ = 0.33 (DCM/MeOH, 20:1, v/v). IR (ATR): 3025, 2931, 2802, 1627, 1508, 1454, 1393, 1333, 1271, 1248, 1205, 1156, 1126, 1113, 1076, 1040, 968, 843, 738, 700 cm^-1^. ^1^H-NMR (400.130 MHz, MeOD): δ = 0.94 (1 H, qd, *J_1_ =* 12.4 Hz, *J_2_* = 3.4 Hz), 1.48–1.59 (1 H, m), 1.64–1.66 (1 H, m), 1.68–1.75 (4 H, m), 1.89–2.00 (2 H, m), 2.75–2.80 (1 H, m), 2.86–2.91 (1 H, m), 3.00–3.11 (2 H, m), 3.21 (2 H, dd, *J_1_ =* 9.4 Hz, *J_2_* = 6.6 Hz), 3.23 (3 H, s), 3.29 (2 H, t, *J =* 6.2 Hz), 3.41–3.48 (2 H, m), 5.15 (2 H, s), 7.23–7.32 (5 H, m), 7.38 (1 H, dd, *J_1_* = 9.0 Hz, *J_2_* = 2.6 Hz), 7.60 (1 H, d, *J* = 3.2 Hz), 7.68 (1 H, dd, *J_1_* = 8.6 Hz, *J_2_* = 1.8 Hz), 7.93 (1 H, d, *J* = 9.2 Hz), 7.98 (1 H, d, *J* = 8.8 Hz), 8.36 (1 H, d, *J* = 2.0 Hz). ^13^C-NMR (100.613 MHz, MeOD): δ = 25.56, 29.53, 30.19, 36.13, 47.68, 54.00, 54.56, 55.09, 58.80, 58.97, 64.47, 70.90, 109.89, 116.84, 122.34, 122.55, 128.54, 128.60, 129.41, 130.49, 130.93, 131.27, 132.55, 134.82, 138.35, 138.79, 157.32. HRMS (ESI+): *m/z* calcd for C_29_H_36_N_3_O_4_S [M+H]^+^ 522.2427; found 522.2422. HPLC purity, 96% at 254.16 nm (method C, *t*_R_ = 11.93 min).

***Synthesis of (±)-N-((1-benzylpiperidin-3-yl)methyl)-N-(2-methoxyethyl)naphthalene-2-sulfonamide (2)***

Synthesized from (±)-*N*-((1-benzylpiperidin-3-yl)methyl)-2-methoxyethanamine^10^ (**10**) and naphthalene-2-sulfonyl chloride via general procedure 1. Purified by flash column chromatography using DCM/MeOH (30:1, v/v) as the eluent to produce 1.654 g of **2** as a colorless oil (90% yield). *R*_f_ = 0.57 (DCM/MeOH, 10:1, v/v). IR (ATR): 2928, 2803, 1452, 1333, 1154, 1115, 1072, 983, 883, 859, 817, 732, 699, 650, 615 cm^-1^. ^1^H-NMR (400.130 MHz, CDCl_3_): δ = 0.95–1.04 (1 H, m), 1.54 (1 H, bs), 1.66–1.74 (3 H, m), 1.98 (2 H, bs), 2.76 (2 H, d, *J* = 30.4 Hz), 3.06–3.16 (2 H, m), 3.21 (3 H, s), 3.30 (2 H, t, *J* = 6.3 Hz), 3.45–3.48 (4 H, m), 7.23–7.30 (5 H, m), 7.59–7.66 (2 H, m), 7.76 (1 H, dd, *J_1_* = 8.6 Hz, *J_2_* = 1.8 Hz), 7.89–7.97 (3 H, m), 8.38 (1 H, d, *J* = 1.4 Hz). ^13^C-NMR (100.613 MHz, CDCl_3_): δ = 24.54, 28.21, 34.82, 48.04, 53.21, 53.89, 57.60, 58.66, 63.44, 71.10, 122.52, 126.85, 127.39, 127.78, 128.05, 128.36, 128.56, 129.07, 129.11, 129.14, 132.07, 134.59, 136.40, 138.25. HRMS (ESI+): *m/z* calcd for C_26_H_33_N_2_O_3_S [M+H]^+^ 453.2212; found 453.2209. HPLC purity, 99% at 254.16 nm (method C, *t*_R_ = 14.10 min).

***Synthesis of (±)-N-((1-benzylpiperidin-3-yl)methyl)-N-(3-methoxypropyl)naphthalene-2-sulfonamide (5)***

Synthesized from (±)-*N*-((1-benzylpiperidin-3-yl)methyl)-3-methoxypropan-1-amine^10^ (**11**) and naphthalene-2-sulfonyl chloride via general procedure 1. Purified by flash column chromatography using DCM/MeOH (25:1, v/v) as the eluent to produce 1.565 g of **5** as a slightly golden oil (87% yield). *R*_f_ = 0.65 (DCM/MeOH, 10:1, v/v). IR (ATR): 2927, 2804, 1453, 1334, 1196, 1154, 1113, 1072, 967, 860, 817, 739, 699, 650, 614 cm^-1^. ^1^H-NMR (400.130 MHz, CDCl_3_): δ = 0.95–1.05 (1 H, m), 1.53 (1 H, bs), 1.65–1.83 (5 H, m), 1.97 (2 H, bs), 2.75 (2 H, d, *J* = 32.9), 3.01–3.14 (2 H, m), 3.16–3.27 (5 H, m), 3.31 (2 H, t, *J* = 6.0 Hz), 3.44 (2 H, bs), 7.24–7.28 (5 H, m), 7.59–7.66 (2 H, m), 7.75 (1 H, dd, *J_1_* = 8.7 Hz, *J_2_* = 1.8 Hz), 7.89–7.98 (3 H, m), 8.37 (1 H, d, *J* = 1.3 Hz). ^13^C-NMR (100.613 MHz, CDCl_3_): δ = 24.46, 28.26, 28.99, 34.74, 46.28, 52.57, 53.83, 57.66, 58.46, 63.30, 69.68, 122.46, 126.83, 127.36, 127.75, 128.02, 128.30, 128.51, 129.04, 129.07, 129.18, 132.06, 134.53, 136.36, 138.17. HRMS (ESI+): *m/z* calcd for C_27_H_35_N_2_O_3_S [M+H]^+^ 467.2368; found 467.2365. HPLC purity, 98% at 254.16 nm (method C, *t*_R_ = 14.84 min).

***Synthesis of (±)-tert-butyl (1-(3-fluorobenzyl)piperidin-3-yl)methyl(2-methoxyethyl)carbamate (28)***

Synthesized from (±)-*tert*-butyl 2-methoxyethyl(piperidin-3-ylmethyl)carbamate^11^ (**26**) and 3-fluorobenzaldehide via general procedure 4. Purified by flash column chromatography using DCM/MeOH (28:1, v/v) as the eluent to produce 0.184 g of **28** as a slightly golden oil (63% yield). *R*_f_ = 0.46 (DCM/MeOH, 10:1, v/v). IR (ATR): 2974, 2931, 1689, 1590, 1484, 1451, 1412, 1391, 1365, 1250, 1161, 1117, 777 cm^-1^. ^1^H-NMR (400.130 MHz, CDCl_3_): δ = 0.89–1.00 (1 H, m), 1.42–1.46 (10 H, m), 1.63–1.77 (3 H, m), 1.87–1.98 (2 H, m), 2.72–2.75 (2 H, m), 3.11–3.16 (2 H, m), 3.24–3.37 (5 H, m), 3.39–3.49 (4 H, m), 6.89–6.94 (1 H, m), 7.03–7.06 (2 H, m), 7.21–7.25 (1 H, m). ^13^C-NMR (100.163 MHz, CDCl_3_): δ = 24.88, 28.37, 28.49, 35.63, 36.11, 47.16, 51.37, 51.93, 54.07, 54.23, 57.76, 58.12, 58.81, 62.96, 70.95, 71.13, 79.33, 79.43, 113.77 (d, ^2^*J* = 21.3 Hz), 115.64 (d, ^2^*J* = 21.3 Hz), 124.47 (d, ^4^*J* = 2.9 Hz), 129.49 (d, ^3^*J* = 8.1 Hz), 141.26 (d, ^3^*J* = 8.1 Hz), 155.66, 162.86 (d, ^1^*J* = 245.76 Hz). HRMS (ESI+): *m/z* calcd for C_21_H_34_N_2_O_3_F [M+H]^+^ 381.2553; found 381.2558. HPLC purity, 96% at 254.16 nm (method D, *t*_R_ = 20.46 min).

***Synthesis of (±)-tert-butyl (1-(4-fluorobenzyl)piperidin-3-yl)methyl(2-methoxyethyl)carbamate (27)***

Synthesized from (±)-*tert*-butyl 2-methoxyethyl(piperidin-3-ylmethyl)carbamate^11^ (**26**) and 4-fluorobenzaldehide via general procedure 4. Purified by flash column chromatography using DCM/MeOH (25:1, v/v) as the eluent to produce 0.229 g of **27** as a slightly golden oil (79% yield). *R*_f_ = 0.36 (DCM/MeOH, 10:1, v/v). IR (ATR): 2930, 1689, 1509, 1464, 1413, 1365, 1345, 1220, 1155, 1116, 1090, 1073, 824 cm^-1^. ^1^H-NMR (400.130 MHz, CDCl_3_): δ = 0.87–1.00 (1 H, m), 1.37–1.46 (10 H, m), 1.62–1.74 (3 H, m), 1.86–1.94 (2 H, m), 2.71–2.74 (2 H, m), 3.12 (2 H, bs), 3.24–3.49 (9 H, m), 6.97 (2 H, t, *J* = 8.60 Hz), 7.23–7.27 (2 H, m). ^13^C-NMR (100.613 MHz, CDCl_3_): δ = 24.87, 28.36, 28.54, 35.59, 36.08, 47.11, 51.37, 51.94, 53.92, 54.15, 57.64, 58.06, 58.81, 62.74, 70.92, 71.11, 79.32, 79.40, 114.87 (d, ^2^*J* = 21.3 Hz), 130.48 (d, ^3^*J* = 8.1 Hz), 134.01 (d, ^4^*J* = 2.9 Hz), 155.64, 161.88 (d, ^1^*J* = 244.3 Hz). HRMS (ESI+): *m/z* calcd for C_21_H_34_N_2_O_3_F [M+H]^+^ 381.2553; found 381.2557. HPLC purity, 95% at 254.16 nm (method D, *t*_R_ = 19.66 min).

***Synthesis of (±)-tert-butyl (1-(4-(dimethylamino)benzyl)piperidin-3-yl)methyl(2-methoxyethyl)carbamate (31)***

Synthesized from (±)-*tert*-butyl 2-methoxyethyl(piperidin-3-ylmethyl)carbamate^11^ (**26**) and 4-(dimethylamino)benzaldehyde via general procedure 4. Purified by flash column chromatography using DCM/MeOH (15:1, v/v) as the eluent to produce 0.192 g of **31** as a slightly golden oil (66% yield). *R*_f_ = 0.39 (DCM/MeOH, 10:1, v/v). IR (ATR): 2974, 2929, 2797, 1689, 1614, 1522, 1463, 1412, 1364, 1344, 1239, 1160, 1116, 805 cm^-1^. ^1^H-NMR (400.130 MHz, CDCl_3_): δ = 0.87–0.99 (1 H, m), 1.38–1.48 (10 H, m), 1.63–1.68 (3 H, m), 1.92 (2 H, bs), 2.76–2.88 (2 H, m), 2.93 (6 H, s), 3.10–3.16 (2 H, m), 3.28–3.49 (9 H, m), 6.66–6.70 (2 H, m), 7.14–7.21 (2 H, m). ^13^C-NMR (100.613 MHz, CDCl_3_): δ = 24.36, 24.73, 28.14, 28.38, 28.53, 35.12, 35.88, 47.00, 51.20, 51.92, 53.55, 57.17, 57.72, 58.79, 62.66, 62.91, 70.86, 71.05, 79.35, 79.40, 112.30, 130.30, 130.65, 149.82, 149.98, 155.67. HRMS (ESI+): *m/z* calcd for C_23_H_40_N_3_O_3_ [M+H]^+^ 406.3070; found 406.3081. HPLC purity, 99% at 254.16 nm (method D, *t*_R_ = 19.61 min).

***Synthesis of (±)-tert-butyl (1-(3-cyanobenzyl)piperidin-3-yl)methyl(2-methoxyethyl)carbamate (30)***

Synthesized from (±)-*tert*-butyl 2-methoxyethyl(piperidin-3-ylmethyl)carbamate^11^ (**26**) and 3-formylbenzonitrile via general procedure 4. Purified by flash column chromatography using DCM/MeOH (26:1, v/v) as the eluent to produce 0.247 g of **30** as a slightly golden oil (77% yield). *R*_f_ = 0.57 (DCM/MeOH, 10:1, v/v). IR (ATR): 2973, 2931, 2229, 1687, 1465, 1413, 1391, 1365, 1284, 1244, 1160, 1116, 690 cm^-1^. ^1^H-NMR (400.130 MHz, CDCl_3_): δ = 0.90–1.01 (1 H, m), 1.41–1.46 (10 H, m), 1.64–1.71 (2 H, m), 1.72–1.81 (1 H, m), 1.86–2.00 (2 H, m), 2.66–2.72 (2 H, m), 3.09–3.17 (2 H, m), 3.25–3.37 (5 H, m), 3.40–3.48 (4 H, m), 7.39 (1 H, t, J = 7.8 Hz), 7.53 (2 H, t, J = 6.8 Hz), 7.63 (1 H, s). ^13^C-NMR (100.163 MHz, CDCl_3_): δ = 24.80, 28.23, 28.35, 35.62, 36.08, 47.20, 51.32, 51.86, 54.05, 54.19, 57.80, 58.18, 58.82, 62.58, 70.96, 71.15, 79.36, 79.44, 112.22, 118.94, 128.94, 130.68, 132.31, 132.37, 133.24, 133.39, 140.28, 155.62. HRMS (ESI+): *m/z* calcd for C_22_H_34_N_3_O_3_ [M+H]^+^ 388.2600; found 388.2610. HPLC purity, 96% at 254.16 nm (method D, *t*_R_ = 17.81 min).

***Synthesis of (±)-tert-butyl (1-(4-cyanobenzyl)piperidin-3-yl)methyl(2-methoxyethyl)carbamate (29)***

Synthesized from (±)-*tert*-butyl 2-methoxyethyl(piperidin-3-ylmethyl)carbamate^11^ (**26**) and 4-formylbenzonitrile via general procedure 4. Purified by flash column chromatography using DCM/MeOH (29:1, v/v) as the eluent to produce 0.247 g of **29** as a slightly golden oil (79% yield). *R*_f_ = 0.55 (DCM/MeOH, 10:1, v/v). IR (ATR): 2974, 2931, 2227, 1687, 1465, 1413, 1391, 1365, 1298, 1244, 1159, 1116, 818 cm^-1^. ^1^H-NMR (400.130 MHz, CDCl_3_): δ = 0.90–1.02 (1 H, m), 1.41–1.46 (10 H, m), 1.63–1.80 (3 H, m), 1.85–1.99 (2 H, m), 2.69 (2 H, bs), 3.10–3.16 (2 H, m), 3.27–3.37 (5 H, m), 3.40–3.43 (1 H, m), 3.47–3.51 (3 H, m), 7.43 (2 H, d, *J* = 8.2 Hz), 7.58 (2 H, d, *J* = 8.0 Hz). ^13^C-NMR (100.613 MHz, CDCl_3_): δ = 24.79, 28.16, 28.34, 35.59, 36.08, 47.19, 51.32, 51.83, 54.10, 54.29, 57.82, 58.28, 58.82, 62.92, 62.96, 70.96, 71.14, 79.41, 110.68, 118.97, 129.37, 129.53, 131.99, 144.42, 155.62. HRMS (ESI+): *m/z* calcd for C_22_H_34_N_3_O_3_ [M+H]^+^ 388.2600; found 388.2608. HPLC purity, 97% at 254.16 nm (method D, *t*_R_ = 17.81 min).

***Synthesis of (±)-N-((1-(3-fluorobenzyl)piperidin-3-yl)methyl)-2-methoxyethanamine di(2,2,2-trifluoroacetate) (33)***

Synthesized from (±)-*tert*-butyl (1-(3-fluorobenzyl)piperidin-3-yl)methyl(2-methoxyethyl)carbamate (**28**) via general procedure 5 to produce 0.163 g of **33** as a slightly golden oil (92% yield). HRMS (ESI+): *m/z* calcd for C_16_H_26_N_2_OF [M+H]^+^ 281.2029; found 281.2034.

***Synthesis of (±)-N-((1-(4-fluorobenzyl)piperidin-3-yl)methyl)-2-methoxyethanamine di(2,2,2-trifluoroacetate) (32)***

Synthesized from (±)-*tert*-butyl (1-(4-fluorobenzyl)piperidin-3-yl)methyl(2-methoxyethyl)carbamate (**27**) via general procedure 5 to produce 0.230 g of **32** as a slightly golden oil (94% yield). HRMS (ESI+): *m/z* calcd for C_16_H_26_N_2_OF [M+H]^+^ 281.2029; found 281.2046.

***Synthesis of (±)-4-((3-((2-methoxyethylamino)methyl)piperidin-1-yl)methyl)-N,N-dimethylaniline tri(2,2,2-trifluoroacetate) (36)***

Synthesized from (±)-*tert*-butyl (1-(4-(dimethylamino)benzyl)piperidin-3-yl)methyl(2-methoxyethyl)carbamate (**31**) via general procedure 5 to produce 0.220 g of **36** as a slightly golden oil (91% yield). HRMS (ESI+): *m/z* calcd for C_18_H_32_N_3_O [M+H]^+^ 306.2545; found 306.2541.

***Synthesis of (±)-3-((3-((2-methoxyethylamino)methyl)piperidin-1-yl)methyl)benzonitrile di(2,2,2-trifluoroacetate) (35)***

Synthesized from (±)-*tert*-butyl (1-(3-cyanobenzyl)piperidin-3-yl)methyl(2-methoxyethyl)carbamate (**30**) via general procedure 5 to produce 0.229 g of **35** as a slightly golden oil (90% yield). HRMS (ESI+): *m/z* calcd for C_17_H_26_N_3_O [M+H]^+^ 288.2076; found 288.2069.

***Synthesis of (±)-4-((3-((2-methoxyethylamino)methyl)piperidin-1-yl)methyl)benzonitrile di(2,2,2-trifluoroacetate) (34)***

Synthesized from (±)-*tert*-butyl (1-(4-cyanobenzyl)piperidin-3-yl)methyl(2-methoxyethyl)carbamate (**29**) via general procedure 7 to produce 0.232 g of **34** as a slightly golden oil (93% yield). HRMS (ESI+): *m/z* calcd for C_17_H_26_N_3_O [M+H]^+^ 288.2076; found 288.2081.

***Synthesis of (±)-N-((1-(3-fluorobenzyl)piperidin-3-yl)methyl)-N-(2-methoxyethyl)naphthalene-2-sulfonamide (38)***

Synthesized from (±)-*N*-((1-(3-fluorobenzyl)piperidin-3-yl)methyl)-2-methoxyethanamine di(2,2,2-trifluoroacetate) (**33**) and naphthalene-2-sulfonyl chloride via general procedure 1. Purified by flash column chromatography using DCM/MeOH (28:1, v/v) as the eluent to produce 0.121 g of **38** as a slightly golden oil (87% yield). *R*_f_ = 0.46 (DCM/MeOH, 15:1, v/v). IR (ATR): 2932, 1589, 1449, 1335, 1252, 1155, 1129, 1116, 1072, 748, 728, 651, 615 cm^-1^. ^1^H-NMR (400.130 MHz, CDCl_3_): δ = 0.96–1.06 (1 H, m), 1.49–1.55 (1 H, m), 1.66–1.78 (3 H, m), 1.95–2.01 (2 H, m), 2.66–2.79 (2 H, m), 3.11–3.13 (2 H, m), 3.22 (3 H, s), 3.28–3.33 (2 H, m), 3.42–3.51 (4 H, m), 6.90–6.95 (1 H, m), 7.02–7.06 (2 H, m), 7.21–7.25 (1 H, m), 7.59–7.66 (2 H, m), 7.76 (1 H, dd, *J_1_* = 8.7 Hz, *J_2_* = 1.9 Hz), 7.89–7.97 (3 H, m), 8.37–8.38 (1 H, m). ^13^C-NMR (100.613 MHz, CDCl_3_): δ = 24.54, 28.17, 34.85, 48.18, 53.25, 53.99, 57.65, 58.71, 62.73, 71.23, 113.74 (d, ^2^*J* = 21.3 Hz), 115.57 (d, ^2^*J*  = 21.3 Hz), 122.54, 124.44 (d, ^4^*J* = 2.9 Hz), 127.45, 127.83, 128.42, 128.62, 129.16, 129.21, 129.50 (d, ^3^*J* = 8.1 Hz), 132.12, 134.65, 136.39, 141.36 (d, ^3^*J* = 8.1 Hz), 162.86 (d, ^1^*J* = 245.0 Hz). HRMS (ESI+): *m/z* calcd for C_26_H_32_N_2_O_3_SF [M+H]^+^ 471.2118; found 471.2110. HPLC purity, 99% at 254.16 nm (method D, *t*_R_ = 23.04 min).

***Synthesis of (±)-N-((1-(4-fluorobenzyl)piperidin-3-yl)methyl)-N-(2-methoxyethyl)naphthalene-2-sulfonamide (37)***

Synthesized from (±)-*N*-((1-(4-fluorobenzyl)piperidin-3-yl)methyl)-2-methoxyethanamine di(2,2,2-trifluoroacetate) (**32**) and naphthalene-2-sulfonyl chloride via general procedure 1. Purified by flash column chromatography using DCM/MeOH (28:1, v/v) as the eluent to produce 0.146 g of **37** as a slightly golden oil (72% yield). *R*_f_ = 0.37 (DCM/MeOH, 15:1, v/v). IR (ATR): 2930, 1508, 1335, 1219, 1154, 1129, 1116, 1095, 1073, 819, 751, 729, 651 cm^-1^. ^1^H-NMR (400.130 MHz, CDCl_3_): δ = 0.95–1.04 (1 H, m), 1.50–1.53 (1 H, m), 1.64–1.76 (3 H, m), 1.94–1.99 (2 H, m), 2.66–2.80 (2 H, m), 3.11 (2 H, d, *J* = 7.5 Hz), 3.21 (3 H, s), 3.28–3.33 (2 H, m), 3.41–3.47 (4 H, m), 6.95–7.00 (2 H, m), 7.22–7.25 (2 H, m), 7.59–7.67 (2 H, m), 7.76 (1 H, dd, *J_1_* = 8.7 Hz, *J_2_* = 1.9 Hz), 7.89–7.97 (3 H, m), 8.37–8.38 (1 H, m). ^13^C-NMR (100.613 MHz, CDCl_3_): δ = 24.58, 28.26, 34.87, 48.14, 53.27, 53.86, 57.55, 58.71, 62.53, 71.18, 114.88 (d, ^2^*J* = 21.3 Hz), 122.56, 127.47, 127.84, 128.42, 128.63, 129.17, 129.20, 130.52 (d, ^3^*J* = 8.1 Hz), 132.13, 134.03 (d, ^4^*J* = 2.9 Hz), 134.66, 136.44, 161.89 (d, ^1^*J* = 245.0 Hz). HRMS (ESI+): *m/z* calcd for C_26_H_32_N_2_O_3_SF [M+H]^+^ 471.2118; found 471.2129. HPLC purity, 99% at 254.16 nm (method D, *t*_R_ = 22.09 min).

***Synthesis of (±)-N-((1-(4-(dimethylamino)benzyl)piperidin-3-yl)methyl)-N-(2-methoxyethyl)naphthalene-2-sulfonamide (41)***

Synthesized from (±)-4-((3-((2-methoxyethylamino)methyl)piperidin-1-yl)methyl)-*N*,*N*-dimethylaniline tri(2,2,2-trifluoroacetate) (**36**) and naphthalene-2-sulfonyl chloride via general procedure 1. Purified by flash column chromatography using DCM/MeOH (15:1, v/v) as the eluent to produce 0.160 g of **41** as a slightly golden oil (95% yield). *R*_f_ = 0.61 (DCM/MeOH, 7:1, v/v). IR (ATR): 2929, 1613, 1521, 1335, 1197, 1155, 1129, 1072, 808, 750, 727, 651, 615 cm^-1^. ^1^H-NMR (400.130 MHz, CDCl_3_): δ = 0.94–1.03 (1 H, m), 1.66–1.76 (4 H, m), 1.92–2.07 (2 H, m), 2.76–2.93 (9 H, m), 3.10–3.20 (5 H, m), 3.30–3.33 (2 H, m), 3.42–3.51 (3 H, m), 6.65–6.70 (2 H, m), 7.12–7.18 (2 H, m), 7.59–7.66 (2 H, m), 7.76 (1 H, dd, *J_1_* = 8.7 Hz, *J_2_* = 1.8 Hz), 7.89–7.98 (3 H, m), 8.37–8.38 (1 H, m). ^13^C-NMR (100.613 MHz, CDCl_3_): δ = 24.12, 28.00, 34.38, 40.59, 48.08, 53.08, 53.27, 56.99, 58.68, 62.52, 71.01, 112.25, 122.55, 127.43, 127.82, 128.42, 128.60, 129.16, 129.20, 130.60, 132.11, 134.65, 136.45, 149.98. HRMS (ESI+): *m/z* calcd for C_28_H_38_N_3_O_3_S [M+H]^+^ 496.2634; found 496.2638. HPLC purity, 95% at 254.16 nm (method D, *t*_R_ = 22.42 min).

***Synthesis of (±)-N-((1-(3-cyanobenzyl)piperidin-3-yl)methyl)-N-(2-methoxyethyl)naphthalene-2-sulfonamide (40)***

Synthesized from (±)-3-((3-((2-methoxyethylamino)methyl)piperidin-1-yl)methyl)benzonitrile di(2,2,2-trifluoroacetate) (**35**) and naphthalene-2-sulfonyl chloride via general procedure 1. Purified by flash column chromatography using DCM/MeOH (28:1, v/v) as the eluent to produce 0.201 g of **40** as a slightly golden oil (97% yield). *R*_f_ = 0.63 (DCM/MeOH, 15:1, v/v). IR (ATR): 2931, 2228, 1334, 1155, 1129, 1115, 1073, 987, 751, 727, 689, 651, 615 cm^-1^. ^1^H-NMR (400.130 MHz, CDCl_3_): δ = 0.97–1.06 (1 H, m), 1.48–1.55 (1 H, m), 1.65–1.75 (2 H, m), 1.78–1.84 (1 H, m), 1.96–2.03 (2 H, m), 2.62–2.79 (2 H, m), 3.07–3.19 (2 H, m), 3.22 (3 H, s), 3.29–3.34 (2 H, m), 3.44–3.48 (4 H, m), 7.37–7.42 (1 H, m), 7.52–7.54 (2 H, m), 7.59–7.67 (3 H, m), 7.76 (1 H, dd, *J_1_* = 8.6 Hz, *J_2_* = 1.8 Hz), 7.90–7.98 (3 H, m), 8.37–8.38 (1 H, m). ^13^C-NMR (100.613 MHz, CDCl_3_): δ = 24.54, 28.13, 48.24, 53.22, 53.93, 57.74, 58.73, 62.40, 71.24, 112.19, 119.02, 122.49, 127.49, 127.84, 128.39, 128.65, 128.93, 129.15, 129.23, 130.62, 132.12, 132.26, 133.26, 134.64, 136.41, 140.47. HRMS (ESI+): *m/z* calcd for C_27_H_32_N_3_O_3_S [M+H]^+^ 478.2164; found 478.2156. HPLC purity, 98% at 254.16 nm (method D, *t*_R_ = 20.13 min).

***Synthesis of (±)-N-((1-(4-cyanobenzyl)piperidin-3-yl)methyl)-N-(2-methoxyethyl)naphthalene-2-sulfonamide (39)***

Synthesized from (±)-4-((3-((2-methoxyethylamino)methyl)piperidin-1-yl)methyl)benzonitrile di(2,2,2-trifluoroacetate) (**34**) and naphthalene-2-sulfonyl chloride via general procedure 1. Purified by flash column chromatography using DCM/MeOH (28:1, v/v) as the eluent to produce 0.196 g of **39** as a slightly golden oil (92% yield). *R*_f_ = 0.56 (DCM/MeOH, 15:1, v/v). IR (ATR): 2932, 2226, 1334, 1155, 1129, 1114, 1073, 857, 816, 751, 728, 651, 615 cm^-1^. ^1^H-NMR (400.130 MHz, CDCl_3_): δ = 0.97–1.06 (1 H, m), 1.47–1.55 (1 H, m), 1.65–1.74 (2 H, m), 1.78–1.84 (1 H, m), 1.95–2.03 (2 H, m), 2.62–2.79 (2 H, m), 3.07–3.16 (2 H, m), 3.21 (3 H, s), 3.28–3.34 (2 H, m), 3.44–3.49 (4 H, m), 7.40 (2 H, d, *J* = 7.9 Hz), 7.56–7.67 (4 H, m), 7.75 (1 H, dd, *J_1_* = 8.6 Hz, *J_2_* = 1.8 Hz), 7.90–7.97 (3 H, m), 8.36–8.37 (1 H, m). ^13^C-NMR (100.613 MHz, CDCl_3_): δ = 24.55, 28.09, 34.88, 48.21, 53.20, 54.03, 57.76, 58.71, 62.78, 71.24, 110.59, 119.01, 122.49, 127.51, 127.83, 128.39, 128.67, 129.13, 129.20, 129.35, 131.97, 132.11, 134.64, 136.37, 144.62. HRMS (ESI+): *m/z* calcd for C_27_H_32_N_3_O_3_S [M+H]^+^ 478.2164; found 478.2150. HPLC purity, 96% at 254.16 nm (method D, *t*_R_ = 20.08 min).

***Synthesis of (±)-N-((1-benzylpiperidin-3-yl)methyl)naphthalene-2-sulfonamide (7)***

Synthesized from (±)-(1-benzylpiperidin-3-yl)methanamine^10^ (**45**) and naphthalene-2-sulfonyl chloride via general procedure 1. Purified by flash column chromatography using DCM/MeOH (20:1, v/v) as the eluent to produce 1.288 g of **7** as a colorless oil that solidified into a white solid after cooling (43% yield). *R*_f_ = 0.43 (DCM/MeOH, 10:1, v/v). IR (ATR): 3062, 2938, 2816, 2769, 1467, 1452, 1328, 1159, 1068, 972, 817, 759, 701, 658, 640, 614 cm^-1^. ^1^H-NMR (400.130 MHz, CDCl_3_): δ = 1.02–1.11 (1 H, m), 1.45–1.55 (1 H, m), 1.60–1.71 (2 H, m), 1.76–1.90 (2 H, m), 2.09–2.15 (1 H, m), 2.52–2.67 (2 H, m), 2.85–2.97 (2 H, m), 3.38–3.46 (2 H, m), 5.20 (1 H, bs), 7.22–7.31 (5 H, m), 7.59–7.67 (2 H, m), 7.78 (1 H, dd, *J_1_* = 8.7 Hz, *J_2_* = 1.9 Hz), 7.90–7.97 (3 H, m), 8.41 (1 H, t, *J* = 0.9 Hz). ^13^C-NMR (100.613 MHz, CDCl_3_): δ = 24.07, 28.06, 35.36, 47.02, 53.78, 57.14, 63.23, 122.23, 126.95, 127.41, 127.80, 128.11, 128.26, 128.60, 129.05, 129.12, 129.38, 132.03, 134.62, 136.64, 137.77. HRMS (ESI+): *m/z* calcd for C_23_H_27_N_2_O_2_S [M+H]^+^ 395.1793; found 395.1784. HPLC purity, 96% at 254.16 nm (method C, *t*_R_ = 10.35 min).

***Synthesis of (±)-N-((1-benzylpiperidin-3-yl)methyl)-N-methylnaphthalene-2-sulfonamide (8)***

Synthesized from (±)-*N*-((1-benzylpiperidin-3-yl)methyl)naphthalene-2-sulfonamide (**7**) and methyl iodide via general procedure 6. Purified by flash column chromatography using DCM/MeOH (24:1, v/v) as the eluent to produce 0.076 g of **8** as a colorless oil that solidified into a white solid after cooling (70% yield). *R*_f_ = 0.29 (DCM/MeOH, 20:1, v/v). IR (ATR): 2937, 2809, 1453, 1334, 1155, 1132, 1070, 960, 889, 740, 653 cm^-1^. ^1^H-NMR (400.130 MHz, CDCl_3_): δ = 1.00–1.10 (1 H, m), 1.66–1.77 (3 H, m), 1.82–2.04 (3 H, m), 2.74 (4 H, s), 2.84–2.97 (3 H, m), 3.43–3.60 (2 H, m), 7.21–7.25 (1 H, m), 7.30 (4 H, d, *J* = 4.4 Hz), 7.60–7.67 (2 H, m), 7.74 (1 H, dd, *J_1_* = 8.6, *J_2_* = 1.8 Hz), 7.90–7.99 (3 H, m), 8.34–8.35 (1 H, s). ^13^C-NMR (100.613 MHz, CDCl_3_): δ = 24.48, 28.12, 34.20, 35.23, 53.78, 57.68, 63.38, 122.63, 126.88, 127.43, 127.81, 128.08, 128.53, 128.60, 120.09, 129.17, 132.11, 134.33, 134.63, 138.13. HRMS (ESI+): *m/z* calcd for C_24_H_29_N_2_O_2_S [M+H]^+^ 409.1950; found 409.1939. HPLC purity, 99% at 254.16 nm (method D, *t*_R_ = 21.57 min).

***Synthesis of (±)-N-((1-benzylpiperidin-3-yl)methyl)-N-butylnaphthalene-2-sulfonamide (48)***

Synthesized from (±)-*N*-((1-benzylpiperidin-3-yl)methyl)naphthalene-2-sulfonamide (**7**) and *n*-butyl bromide via general procedure 2. Purified by flash column chromatography using DCM/MeOH (25:1, v/v) as the eluent to produce 0.095 g of **48** as a slightly golden oil (83% yield). *R*_f_ = 0.33 (DCM/MeOH, 20:1, v/v). IR (ATR): 2931, 2871, 1454, 1335, 1154, 1130, 1072, 746, 699, 652, 615 cm^-1^. ^1^H-NMR (400.130 MHz, CDCl_3_): δ = 0.85 (3 H, t, *J* = 7.3 Hz), 0.94–1.03 (1 H, m), 1.22 (2 H, h, *J* = 7.4 Hz), 1.42–1.50 (2 H, m), 1.62–1.75 (4 H, m), 1.90–1.99 (2 H, m), 2.76 (2 H, d, *J* = 31.6 Hz), 3.00–3.13 (4 H, m), 3.40–3.49 (2 H, m), 7.21–7.25 (1 H, m), 7.28–7.32 (4 H, m), 7.59–7.66 (2 H, m), 7.74 (1 H, dd, *J_1_* = 8.6 Hz, *J_2_* = 1.8 Hz), 7.89–7.98 (3 H, m), 8.35–8.36 (1 H, m). ^13^C-NMR (100.613 MHz, CDCl_3_): δ = 13.63, 19.95, 24.53, 28.31, 30.62, 34.86, 48.59, 52.10, 53.83, 57.70, 63.35, 122.48, 126.87, 127.35, 127.77, 128.05, 128.22, 128.47, 129.07, 129.09, 129.14, 132.08, 134.51, 136.65, 138.05. HRMS (ESI+): *m/z* calcd for C_27_H_35_N_2_O_2_S [M+H]^+^ 451.2419; found 451.2413. HPLC purity, 99% at 254.16 nm (method E, *t*_R_ = 23.99 min).

***Synthesis of (±)-N-((1-benzylpiperidin-3-yl)methyl)-N-ethylnaphthalene-2-sulfonamide (46)***

Synthesized from (±)-*N*-((1-benzylpiperidin-3-yl)methyl)naphthalene-2-sulfonamide (**7**) and ethyl bromide via general procedure 6. Purified by flash column chromatography using DCM/MeOH (25:1, v/v) as the eluent to produce 0.078 g of **46** as a colorless oil that solidified into a white solid after cooling (73% yield). *R*_f_ = 0.30 (DCM/MeOH, 20:1, v/v). IR (ATR): 2937, 2808, 1329, 1292, 1151, 1109, 995, 887, 749, 724, 652 cm^-1^. ^1^H-NMR (400.130 MHz, CDCl_3_): δ = 0.97–1.06 (4 H, m), 1.65–1.81 (4 H, m) 1.92–2.01 (2 H, m), 2.78 (2 H, d, *J* = 39.7 Hz), 3.02–3.14 (2 H, m), 3.24 (2 H, q, *J* = 7.2 Hz), 3.42–3.53 (2 H, m), 7.21–7.25 (1 H, m), 7.27–7.32 (4 H, m), 7.59–7.66 (2 H, m), 7.74 (1 H, dd, *J_1_* = 8.7 Hz, *J_2_* = 1.8 Hz), 7.89–7.97 (3 H, m), 8.36–8.37 (1 H, m). ^13^C-NMR (100.613 MHz, CDCl_3_): δ = 13.74, 24.53, 28.27, 34.78, 43.02, 51.26, 53.82, 57.70, 63.35, 122.43, 126.86, 127.36, 127.77, 128.05, 128.19, 128.49, 129.07, 129.18, 132.09, 134.52, 136.84, 138.13. HRMS (ESI+): *m/z* calcd for C_25_H_31_N_2_O_2_S [M+H]^+^ 423.2106; found 423.2103. HPLC purity, 99% at 254.16 nm (method D, *t*_R_ = 23.63 min).

***Synthesis of (±)-N-((1-benzylpiperidin-3-yl)methyl)-N-propylnaphthalene-2-sulfonamide (47)***

Synthesized from (±)-*N*-((1-benzylpiperidin-3-yl)methyl)naphthalene-2-sulfonamide (**7**) and *n*-propyl bromide via general procedure 6. Purified by flash column chromatography using DCM/MeOH (25:1, v/v) as the eluent to produce 0.091 g of **47** as a slightly golden oil (82% yield). *R*_f_ = 0.32 (DCM/MeOH, 20:1, v/v). IR (ATR): 2934, 2805, 1453, 1330, 1152, 1130, 1071, 966, 866, 747, 652, 614 cm^-1^. ^1^H-NMR (400.130 MHz, CDCl_3_): δ = 0.81 (3 H, t, J = 7.4 Hz), 0.94–1.04 (1 H, m), 1.46–1.55 (3 H, m), 1.63–1.75 (3 H, m), 1.94–1.99 (2 H, m), 2.71–2.83 (2 H, m), 3.01–3.11 (4 H, m), 3.41–3.50 (2 H, m), 7.21–7.25 (1 H, m), 7.29–7.32 (4 H, m), 7.59–7.66 (2 H, m), 7.74 (1 H, dd, *J_1_* = 8.7 Hz, *J_2_* = 1.9 Hz), 7.89–7.98 (3 H, m), 8.36–8.37 (1 H, m). ^13^C-NMR (100.613 MHz, CDCl_3_): δ = 11.16, 21.85, 24.51, 28.30, 34.87, 50.50, 52.14, 53.81, 57.67, 63.33, 122.45, 126.85, 127.34, 127.75, 128.03, 128.18, 128.46, 129.06, 129.08, 129.14, 132.06, 134.49, 136.67, 138.05. HRMS (ESI+): *m/z* calcd for C_26_H_33_N_2_O_2_S [M+H]^+^ 437.2263; found 437.2255. HPLC purity, 99% at 254.16 nm (method F, *t*_R_ = 20.13 min).

***Synthesis of (±)-tert-butyl (1-(3-methylbenzyl)piperidin-3-yl)methylcarbamate (54)***

Synthesized from (±)-*tert*-butyl piperidin-3-ylmethylcarbamate^14^ (**51**) and 1-(chloromethyl)-3-methylbenzene via general procedure 7. Purified by flash column chromatography using DCM/MeOH (20:1, v/v) then DCM/MeOH (9:1, v/v) as the eluent to produce 0.246 g of **54** as a pale brown solid (55% yield). *R*_f_ = 0.32 (DCM/MeOH, 9:1, v/v). IR (ATR): 3347, 2975, 2930, 2796, 1693, 1514, 1453, 1390, 1365, 1250, 1167, 776 cm^-1^. ^1^H-NMR (400.130 MHz, CDCl_3_): δ = 0.93–1.03 (1 H, m), 1.42 (9 H, s), 1.50–1.61 (1 H, m), 1.63–1.80 (4 H, m), 1.97 (1 H, t, *J =* 10.2 Hz), 2.33 (3 H, s), 2.70–2.82 (2 H, m), 2.95–3.03 (2 H, m), 3.45 (2 H, dd, *J_1_ =* 20.8 Hz, *J_2_* = 13.0 Hz), 4.69 (1 H, t, *J =* 6.0 Hz), 7.04–7.11 (3 H, m), 7.19 (1 H, t, *J =* 7.6 Hz). ^13^C-NMR (100.613 MHz, CDCl_3_): δ = 21.33, 24.56, 28.27, 28.32, 36.66, 44.32, 53.99, 57.65, 63.45, 78.92, 126.15, 127.66, 127.97, 129.79, 137.65, 137.98, 155.97. HRMS (ESI+): *m/z* calcd for C_19_H_31_N_2_O_2_ [M+H]^+^ 319.2386; found 319.2379. HPLC purity, 97% at 254.16 nm (method C, *t*_R_ = 10.67 min).

***Synthesis of (±)-tert-butyl (1-(2-chloro-4-fluorobenzyl)piperidin-3-yl)methylcarbamate*** ***(55)***

Synthesized from (±)-*tert*-butyl piperidin-3-ylmethylcarbamate^14^ (**51**) and 2-chloro-1-(chloromethyl)-4-fluorobenzene via general procedure 7. Purified by flash column chromatography using DCM/MeOH (99:1, v/v) then DCM/MeOH (20:1, v/v) as the eluent to produce 0.229 g of **55** as a colorless oil (69% yield). *R*_f_ = 0.39 (DCM/MeOH, 50:1, v/v). IR (ATR): 3341, 2976, 2932, 2855, 2794, 1694, 1604, 1514, 1489, 1466, 1452, 1390, 1365, 1250, 1230, 1168, 1040, 897, 856 cm^-1^. ^1^H-NMR (400.130 MHz, CDCl_3_): δ = 0.92–1.02 (1 H, m), 1.38 (9 H, s), 1.48–1.54 (1 H, m), 1.60–1.75 (3 H, m), 1.84 (1 H, t, *J =* 9.8 Hz), 2.04 (1 H, t, *J =* 10.0 Hz), 2.63–2.68 (1 H, m), 2.70–2.75 (1 H, m), 2.98 (2 H, t, *J =* 6.2 Hz), 3.47 (2 H, s), 4.85 (1 H, t, *J =* 5.2 Hz), 6.89 (1 H, td, *J_1_* = 8.4 Hz, *J*_2_ = 2.8 Hz), 7.03 (1 H, dd, *J_1_* = 8.6 Hz, *J*_2_ = 2.6 Hz), 7.39 (1 H, dd, *J_1_* = 8.4 Hz, *J*_2_ = 2.4 Hz). ^13^C-NMR (100.613 MHz, CDCl_3_): δ = 24.46, 28.01, 28.19, 36.59, 44.09, 53.89, 57.57, 58.85, 78.71, 113.51 (d, *^2^J =* 20.4 Hz), 116.28 (d, *^2^J =* 24.0 Hz), 131.29 (d, *^3^J =* 8.8 Hz), 132.00 (d, *^4^J =* 3.6 Hz), 134.32 (d, *^3^J =* 9.5 Hz), 155.89, 161.06 (d, *^3^J =* 246.4 Hz). HRMS (ESI+): *m/z* calcd for C_18_H_27_N_2_O_2_FCl [M+H]^+^ 357.1745; found 357.1735. HPLC purity, 95% at 254.16 nm (method C, *t*_R_ = 12.61 min).

***Synthesis of (±)-tert-butyl (1-(4-fluorobenzyl)piperidin-3-yl)methylcarbamate*** ***(56)***

Synthesized from (±)-*tert*-butyl piperidin-3-ylmethylcarbamate^14^ (**51**) and 4-fluorobenzaldehyde via general procedure 4. Purified by flash column chromatography using DCM/MeOH (20:1, v/v) as the eluent to produce 0.118 g of **56** as a white solid (39% yield). *R*_f_ = 0.22 (DCM/MeOH, 20:1, v/v). IR (ATR): 3204, 3006, 2963, 2928, 2852, 2788, 1702, 1602, 1547, 1510, 1470, 1445, 1391, 1365, 1280, 1266, 1254, 1225, 1173, 1146, 1132, 1094, 1063, 1035, 1002, 944, 932, 863, 841, 817, 777, 752 cm^-1^. ^1^H-NMR (400.130 MHz, CDCl_3_): δ = 0.92–1.00 (1 H, m), 1.39 (9 H, s), 1.48–1.55 (1 H, m), 1.60–1.72 (4 H, m), 1.93 (1 H, t, *J =* 10.2 Hz), 2.64–2.73 (2 H, m), 2.98 (2 H, t, *J =* 5.8 Hz), 3.39 (2 H, d, *J =* 3.2 Hz), 4.75 (1 H, bs), 6.94 (2 H, tt, *J_1_* = 8.8 Hz, *J_2_* = 2.6 Hz), 7.20–7.25 (2 H, m). ^13^C-NMR (100.613 MHz, CDCl_3_): δ = 24.54, 28.15, 28.22, 36.71, 44.24, 53.87, 57.50, 62.56, 78.87, 114.79 (d, *^2^J =* 21.1 Hz), 130.39 (d, *^3^J =* 7.3 Hz), 134.00 (d, *^4^J =* 3.7 Hz), 155.95, 161.77 (d, *^3^J =* 243.5 Hz). HRMS (ESI+): *m/z* calcd for C_18_H_28_N_2_O_2_F 323.2135; found 323.2140. HPLC purity, 95% at 254.16 nm (method C, *t*_R_ = 9.28 min).

***Synthesis of (±)-tert-butyl (1-(4-cyanobenzyl)piperidin-3-yl)methylcarbamate*** ***(57)***

Synthesized from (±)-*tert*-butyl piperidin-3-ylmethylcarbamate^14^ (**51**) and 4-formylbenzonitrile via general procedure 4. Purified by flash column chromatography using EtOAc/n-hexane (1:1, v/v) as the eluent to produce 0.190 g of **57** as a colorless oil (62% yield). *R*_f_ = 0.13 (EtOAc/*n-*hex, 1:1, v/v). IR (ATR): 3359, 2975, 2931, 2855, 2799, 2228, 1695, 1608, 1508, 1453, 1414, 1391, 1365, 1250, 1166, 1075, 1039, 1020, 1005, 851, 818 cm^-1^. ^1^H-NMR (400.130 MHz, CDCl_3_): δ = 0.93 (1 H, q, *J =* 10.8 Hz), 1.33 (9 H, s), 1.41–1.51 (1 H, m), 1.56–1.75 (4 H, m), 1.93 (1 H, t, *J =* 10.8 Hz), 2.58 (1 H, d, *J =* 11.2 Hz), 2.65 (1 H, d, *J =* 10.0 Hz), 2.93 (2 H, t, *J =* 5.6 Hz), 3.43 (2 H, s), 4.77 (1 H, t, *J =* 5.8 Hz), 7.36 (2 H, d, *J* = 8.0 Hz), 7.50 (2 H, d, *J* = 8.4 Hz). ^13^C-NMR (100.613 MHz, CDCl_3_): δ = 24.39, 27.88, 28.13, 36.66, 43.95, 53.93, 57.54, 62.63, 78.68, 110.30, 118.73, 129.14, 131.73, 144.47, 155.82. HRMS (ESI+): *m/z* calcd for C_19_H_28_N_3_O_2_ [M+H]^+^ 330.2182; found 330.2181. HPLC purity, 96% at 254.16 nm (method C, *t*_R_ = 8.25 min).

***Synthesis of (±)-tert-butyl (1-(pyridin-4-ylmethyl)piperidin-3-yl)methylcarbamate*** ***(52)***

Synthesized from (±)-*tert*-butyl piperidin-3-ylmethylcarbamate^14^ (**51**) and 4-(chloromethyl)pyridine via general procedure 7. Purified by flash column chromatography using DCM/MeOH (20:1, v/v) as the eluent to produce 0.189 g of **52** as a colorless oil (66% yield). *R*_f_ = 0.11 (DCM/MeOH, 20:1, v/v). IR (ATR): 3341, 2975, 2931, 2800, 1695, 1602, 1524, 1452, 1415, 1390, 1364, 1269, 1250, 1167, 1001, 802 cm^-1^. ^1^H-NMR (400.130 MHz, CDCl_3_): *δ* = 0.80–0.90 (1 H, m), 1.26 (9 H, s), 1.35–1.46 (1 H, m), 1.49–1.68 (4 H, m), 1.86 (1 H, t, *J =* 10.0 Hz), 2.53 (1 H, d, *J =* 10.0 Hz), 2.61 (1 H, d, *J =* 6.8 Hz), 2.87 (2 H, t, *J =* 5.6 Hz), 3.31 (2 H, s), 5.14 (1 H, t, *J =* 4.1 Hz), 7.10 (2 H, dd, *J_1_* = 4.4 Hz, *J_2_* = 1.4 Hz), 8.35 (2 H, dd, *J_1_* = 4.4 Hz, *J_2_* = 1.2 Hz). ^13^C NMR (100.613 MHz, CDCl_3_): δ 24.33, 27.82, 28.06, 36.58, 43.91, 53.89, 57.56, 61.84, 78.47, 123.41, 147.79, 149.20, 155.85. HRMS (ESI+): *m/z* calcd for C_17_H_28_N_3_O_2_ [M+H]^+^ 306.2182; found 306.2188. HPLC purity, 96% at 254.16 nm (method C, *t*_R_ = 5.68 min).

***Synthesis of (±)-tert-butyl (1-((2-methylthiazol-4-yl)methyl)piperidin-3-yl)methylcarbamate*** ***(53)***

Synthesized from (±)-*tert*-butyl piperidin-3-ylmethylcarbamate^14^ (**51**) and 4-(chloromethyl)-2-methylthiazole via general procedure 7. Purified by flash column chromatography using DCM/MeOH (20:1, v/v) as the eluent to produce 0.093 g of **53** as a yellow oil (31% yield). *R*_f_ = 0.32 (DCM/MeOH, 20:1, v/v). IR (ATR): 3342, 2974, 2929, 2803, 1694, 1522, 1451, 1390, 1364, 1268, 1249, 1168, 1074, 1038, 1006 cm^-1^. ^1^H-NMR (400.130 MHz, CDCl_3_): δ = 0.98 (1 H, q, *J =* 6.6 Hz), 1.42 (9 H, s), 1.55–1.61 (1 H, m), 1.64–1.73 (3 H, m), 1.80–1.84 (1 H, m), 2.04 (1 H, t, *J =* 9.8 Hz), 2.70 (3 H, s), 2.80–2.84 (2 H, m), 3.02 (2 H, t, *J =* 6.0 Hz), 3.59 (2 H, d, *J =* 1.6 Hz), 4.61 (1 H, bs), 6.93 (1 H, s). ^13^C-NMR (100.613 MHz, CDCl_3_): δ = 19.05, 24.45, 28.10, 28.27, 36.59, 44.12, 54.06, 57.52, 58.56, 78.86, 115.44, 153.09, 155.93, 165.58. HRMS (ESI+): *m/z* calcd for C_16_H_28_N_3_O_2_S [M+H]^+^ 326.1902; found 326.1911. HPLC purity, 95% at 254.16 nm (method C, *t*_R_ = 6.20 min).

***Synthesis of (±)-(1-(3-methylbenzyl)piperidin-3-yl)methanamine di(2,2,2-trifluoroacetate)***  ***(60)***

Synthesized from (±)-*tert*-butyl (1-(3-methylbenzyl)piperidin-3-yl)methylcarbamate (**54**) via general procedure 5 to produce 0.305 g of **60** as a yellow oil (91% yield). HRMS (ESI+): *m/z* calcd for C_14_H_23_N_2_ [M+H]^+^ 219.1861; found 219.1863.

***Synthesis of (±)-(1-(2-chloro-4-fluorobenzyl)piperidin-3-yl)methanamine*** ***di(2,2,2-trifluoroacetate)*** ***(61)***

Synthesized from (±)-*tert*-butyl (1-(2-chloro-4-fluorobenzyl)piperidin-3-yl)methylcarbamate (**55**) via general procedure 5 to produce 0.243 g of **61** as a pale brown solid (94% yield). HRMS (ESI+): *m/z* calcd for C_13_H_19_ClFN_2_ [M+H]^+^ 257.1221; found 257.1224.

***Synthesis of (±)-(1-(4-fluorobenzyl)piperidin-3-yl)methanamine*** ***di(2,2,2-trifluoroacetate)*** ***(62)***

Synthesized from (±)-*tert*-butyl (1-(4-fluorobenzyl)piperidin-3-yl)methylcarbamate (**56**) via general procedure 7 to produce 0.158 g of **62** as a white solid (93% yield). HRMS (ESI+): *m/z* calcd for C_13_H_20_FN_2_ [M+H]^+^ 223.1611; found 223.1616.

***Synthesis of (±)-4-((3-(aminomethyl)piperidin-1-yl)methyl)benzonitrile*** ***di(2,2,2-trifluoroacetate)*** ***(63)***

Synthesized from (±)-*tert*-butyl (1-(4-cyanobenzyl)piperidin-3-yl)methylcarbamate (**57**) via general procedure 7 to produce 0.246 g of **63** as a yellow oil (92% yield). HRMS (ESI+): *m/z* calcd for C_14_H_20_N_3_ [M+H]^+^ 230.1657; found 230.1660.

***Synthesis of (±)-(1-(pyridin-4-ylmethyl)piperidin-3-yl)methanamine*** ***tri(2,2,2-trifluoroacetate)*** ***(58)***

Synthesized from (±)-*tert*-butyl (1-(pyridin-4-ylmethyl)piperidin-3-yl)methylcarbamate (**52**) via general procedure 5 to produce 0.397 g of **58** as a yellow oil (93% yield). HRMS (ESI+): *m/z* calcd for C_12_H_20_N_3_ [M+H]^+^ 206.1657; found 206.1655.

***Synthesis of (±)-(1-((2-methylthiazol-4-yl)methyl)piperidin-3-yl)methanamine*** ***di(2,2,2-trifluoroacetate)*** ***(59)***

Synthesized from (±)-*tert*-butyl (1-((2-methylthiazol-4-yl)methyl)piperidin-3-yl)methylcarbamate (**53**) via general procedure 5 to produce 0.180 g of **59** as a colorless oil (92% yield). HRMS (ESI+): *m/z* calcd for C_11_H_20_N_3_S [M+H]^+^ 226.1378; found 226.1383.

***Synthesis of (±)-N-((1-(3-methylbenzyl)piperidin-3-yl)methyl)naphthalene-2-sulfonamide (66)***

Synthesized from (±)-(1-(3-methylbenzyl)piperidin-3-yl)methanamine di(2,2,2-trifluoroacetate) (**60**) and naphthalene-2-sulfonyl chloride via general procedure 1. Purified by flash column chromatography using DCM/MeOH (50:1, v/v) then DCM/MeOH (20:1, v/v) as the eluent to produce 0.199 g of **66** as a white solid (98% yield). *R*_f_ = 0.43 (DCM/MeOH, 9:1, v/v). IR (ATR): 3055, 2935, 2855, 2820, 2768, 2684, 1716, 1660, 1481, 1465, 1452, 1347, 1326, 1280, 1157, 1133, 1122, 1070, 1017, 974, 866, 825, 807, 774, 751, 704 cm^-1^. ^1^H-NMR (400.130 MHz, CDCl_3_): δ = 0.96–1.05 (1 H, m), 1.45–1.54 (1 H, m), 1.58–1.66 (2 H, m), 1.68–1.75 (1 H, m), 1.79 (1 H, t, *J =* 10.2 Hz), 2.03 (1 H, t, *J =* 9.6 Hz), 2.30 (3 H, s), 2.58–2.64 (1 H, m), 2.69–2.74 (1 H, m), 2.82–2.92 (2 H, m), 3.35 (2 H, d, *J =* 3.2 Hz), 5.77 (1 H, bs,), 7.00 (1 H, d, *J* = 7.6 Hz), 7.03–7.06 (2 H, m,), 7.16 (1 H, d, *J* = 7.8 Hz), 7.58–7.66 (2 H, m), 7.82 (1 H, dd, *J_1_* = 8.6 Hz, *J_2_* = 1.8 Hz), 7.89–7.96 (3 H, m), 8.44 (1 H, d, *J* = 1.2 Hz). ^13^C-NMR (100.613 MHz, CDCl_3_): δ = 21.27, 24.07, 28.09, 35.31, 47.05, 53.85, 57.21, 63.28, 122.22, 126.16, 127.39, 127.70, 127.77, 127.96, 128.23, 128.58, 129.09, 129.35, 129.83, 132.02, 134.59, 136.65, 137.65, 137.67. HRMS (ESI+): *m/z* calcd for C_24_H_29_N_2_O_2_S [M+H]^+^ 409.1950; found 409.1959. HPLC purity, 96% at 254.16 nm (method C, *t*_R_ = 12.03 min).

***Synthesis of (±)-N-((1-(2-chloro-4-fluorobenzyl)piperidin-3-yl)methyl)naphthalene-2-sulfonamide*** ***(67)***

Synthesized from (±)-(1-(2-chloro-4-fluorobenzyl)piperidin-3-yl)methanamine di(2,2,2-trifluoroacetate) (**61**) and naphthalene-2-sulfonyl chloride via general procedure 1. Purified by flash column chromatography using DCM/MeOH (30:1, v/v) then DCM/MeOH (20:1, v/v) as the eluent to produce 0.109 g of **67** as a white solid (61% yield). *R*_f_ = 0.37 (DCM/MeOH, 20:1, v/v). IR (ATR): 3285, 2935, 2852, 2790, 2765, 1709, 1599, 1579, 1491, 1449, 1417, 1352, 1335, 1318, 1310, 1298, 1281, 1256, 1229, 1202, 1154, 1132, 1127, 1074, 1054, 1040, 1020, 901, 850, 811, 776, 748 cm^-1^. ^1^H-NMR (400.130 MHz, CDCl_3_): δ = 1.05–1.12 (1 H, m), 1.45–1.54 (1 H, m), 1.62–1.71 (2 H, m), 1.75–1.81 (1 H, m), 1.95 (1 H, t, *J =* 9.8 Hz), 2.18 (1 H, t, *J =* 9.4 Hz), 2.52–2.58 (1 H, m), 2.63–2.69 (1 H, m), 2.87–2.99 (2 H, m), 3.45 (2 H, s), 5.02 (1 H, bs), 6.91 (1 H, td, *J_1_* = 8.4 Hz, *J_2_* = 2.8 Hz), 7.06 (1 H, dd, *J_1_* = 8.6 Hz, *J_2_* = 2.6 Hz), 7.34 (1 H, dd, *J_1_* = 8.4 Hz, *J_2_* = 6.4 Hz), 7.59–7.67 (2 H, m), 7.79 (1 H, dd, *J_1_* = 8.4 Hz, *J_2_* = 2.0 Hz), 7.89–7.96 (3 H, m), 8.41 (1 H, d, *J* = 1.2 Hz). ^13^C-NMR (100.613 MHz, CDCl_3_): δ = 24.08, 27.89, 35.58, 46.88, 53.73, 57.23, 58.83, 113.67 (d, *^2^J =* 20.4 Hz), 116.41 (d, *^2^J =* 24.8 Hz), 122.14, 127.43, 127.78, 128.26, 128.63, 129.07, 129.38, 131.41 (d, *^3^J =* 8.0 Hz), 131.78 (d, *^4^J =* 2.9 Hz), 131.99, 134.39 (d, *^3^J =* 10.2 Hz), 134.60, 136.49, 161.16 (d, *^3^J =* 246.0 Hz). HRMS (ESI+): *m/z* calcd for C_23_H_25_N_2_O_2_SFCl [M+H]^+^ 447.1309; found 447.1299. HPLC purity, 98% at 254.16 nm (method C, *t*_R_ = 14.34 min).

***Synthesis of (±)-N-((1-(4-fluorobenzyl)piperidin-3-yl)methyl)naphthalene-2-sulfonamide*** ***(68)***

Synthesized from (±)-(1-(4-fluorobenzyl)piperidin-3-yl)methanamine di(2,2,2-trifluoroacetate) (**62**) and naphthalene-2-sulfonyl chloride via general procedure 1. Purified by flash column chromatography using DCM/MeOH (20:1, v/v) as the eluent to produce 0.120 g of **68** as a white solid (76% yield). *R*_f_ = 0.17 (DCM/MeOH, 20:1, v/v). IR (ATR): 3276, 3057, 2932, 2855, 2798, 1602, 1507, 1466, 1439, 1347, 1322, 1219, 1153, 1130, 1074, 1016, 861, 816, 748 cm^-1^. ^1^H-NMR (400.130 MHz, CDCl_3_): δ = 0.98 (1 H, q, *J =* 9.3 Hz), 1.40–1.50 (1 H, m), 1.56–1.65 (2 H, m), 1.69–1.79 (2 H, m), 1.97 (1 H, t, *J =* 9.6 Hz), 2.52–2.59 (1 H, m), 2.65–2.71 (1 H, m), 2.84–2.90 (2 H, m), 3.32 (2 H, d, *J =* 2.8 Hz), 5.67 (1 H, bs), 6.92 (2 H, tt, *J_1_* = 8.6 Hz, *J_2_* = 2.5 Hz), 7.12–7.17 (2 H, m), 7.57–7.65 (2 H, m), 7.82 (1 H, dd, *J_1_* = 8.6 Hz, *J_2_* = 1.8 Hz), 7.88–7.95 (3 H, m), 8.43 (1 H, d, *J* = 1.2 Hz). ^13^C-NMR (100.613 MHz, CDCl_3_): δ = 24.11, 28.05, 35.52, 46.96, 53.60, 57.10, 62.36, 114.84 (d, *^2^J =* 21.1 Hz), 122.19, 127.45, 127.80, 128.25, 128.65, 129.10, 129.39, 130.46 (d, *^3^J =* 7.3 Hz), 132.02, 133.58 (d, *^4^J =* 2.9 Hz), 134.62, 136.58, 161.78 (d, *^1^J =* 243.5 Hz). HRMS (ESI+): *m/z* calcd for C_23_H_26_N_2_O_2_SF [M+H]^+^ 413.1699; found 413.1688. HPLC purity, 97% at 254.16 nm (method C, *t*_R_ = 10.45 min).

***Synthesis of (±)-N-((1-(4-cyanobenzyl)piperidin-3-yl)methyl)naphthalene-2-sulfonamide*** ***(69)***

Synthesized from (±)-4-((3-(aminomethyl)piperidin-1-yl)methyl)benzonitrile di(2,2,2-trifluoroacetate) (**63**) and naphthalene-2-sulfonyl chloride via general procedure 1. Purified by flash column chromatography using DCM/MeOH (20:1, v/v) as the eluent to produce 0.126 g of **69** as a pale red solid (66% yield). *R*_f_ = 0.18 (DCM/MeOH, 20:1, v/v). IR (ATR): 3279, 3054, 2933, 2855, 2800, 2226, 1504, 1436, 1347, 1323, 1154, 1130, 1074, 1019, 906, 861, 815, 784, 748, 730 cm^-1^. ^1^H-NMR (400.130 MHz, CDCl_3_): δ = 0.98 (1 H, q, *J =* 10.0 Hz), 1.37–1.48 (1 H, m), 1.53–1.66 (2 H, m), 1.72–1.82 (2 H, m), 1.96 (1 H, t, *J =* 9.4 Hz), 2.47–2.52 (1 H, m), 2.62–2.68 (1 H, m), 2.87 (2 H, t, *J =* 5.4 Hz), 3.37 (2 H, s), 5.56 (1 H, t, *J =* 6.2 Hz), 7.29 (2 H, d, *J* = 8.8 Hz), 7.48 (2 H, dt, *J_1_* = 8.1 Hz, *J_2_* = 1.8 Hz), 7.56–7.64 (2 H, m), 7.82–7.93 (4 H, m), 8.42 (1 H, d, *J* = 1.2 Hz). ^13^C-NMR (100.613 MHz, CDCl_3_): δ = 24.05, 27.75, 35.73, 46.65, 53.69, 57.19, 62.48, 110.31, 118.89, 122.06, 127.44, 127.74, 128.17, 128.65, 129.01, 129.13, 129.34, 131.83, 131.91, 134.53, 136.46, 144.22. HRMS (ESI+): *m/z* calcd for C_24_H_26_N_3_O_2_S [M+H]^+^ 420.1746; found 420.1735. HPLC purity, 97% at 254.16 nm (method C, *t*_R_ = 9.35 min).

***Synthesis of (±)-N-((1-(pyridin-4-ylmethyl)piperidin-3-yl)methyl)naphthalene-2-sulfonamide*** ***(64)***

Synthesized from (±)-1-(pyridin-4-ylmethyl)piperidin-3-yl)methanamine tri(2,2,2-trifluoroacetate) (**58**) and naphthalene-2-sulfonyl chloride via general procedure 1. Purified by flash column chromatography using DCM/MeOH (20:1, v/v) as the eluent to produce 0.045 g of **64** as a pale brown solid (37% yield). *R*_f_ = 0.52 (DCM/MeOH, 9:1, v/v). IR (ATR): 3215, 3060, 2932, 2856, 2802, 1715, 1604, 1561, 1466, 1438, 1417, 1349, 1324, 1207, 1183, 1152, 1131, 1075, 861, 817, 801, 749, 723 cm^-1^. ^1^H-NMR (400.130 MHz, CDCl_3_): δ = 0.98–1.07 (1 H, m), 1.45–1.56 (1 H, m), 1.61–1.70 (2 H, m), 1.76–1.88 (2 H, m), 2.06 (1 H, t, *J =* 10.4 Hz), 2.56–2.61 (1 H, m), 2.68–2.73 (1 H, m), 2.91 (2 H, t, *J =* 6.2 Hz), 3.41 (2 H, s), 5.37 (1 H, bs), 7.17 (2 H, d, *J* = 6.0 Hz), 7.59–7.66 (2 H, m), 7.79 (1 H, dd, *J_1_* = 8.2 Hz, *J_2_* = 1.8 Hz), 7.89–7.96 (3 H, m), 8.41 (1 H, d, *J* = 1.2 Hz), 8.48 (2 H, d, *J* = 6.0 Hz). ^13^C-NMR (100.613 MHz, CDCl_3_): δ = 24.08, 27.83, 35.75, 46.79, 53.91, 57.30, 61.86, 122.18, 123.81, 127.54, 127.87, 128.31, 128.74, 129.15, 129.46, 132.07, 134.69, 136.64, 147.33, 149.56. HRMS (ESI+): *m/z* calcd for C_22_H_26_N_3_O_2_S [M+H]^+^ 396.1746; found 396.1742. HPLC purity, 95% at 254.16 nm (method C, *t*_R_ = 7.09 min).

***Synthesis of (±)-N-((1-((2-methylthiazol-4-yl)methyl)piperidin-3-yl)methyl)naphthalene-2-sulfonamide*** ***(65)***

Synthesized from (±)-(1-((2-methylthiazol-4-yl)methyl)piperidin-3-yl)methanamine di(2,2,2-trifluoroacetate) (**59**) and naphthalene-2-sulfonyl chloride via general procedure 1. Purified by flash column chromatography using DCM/MeOH (20:1, v/v) as the eluent to produce 0.071 g of **65** as an orange solid (68% yield). *R*_f_ = 0.22 (DCM/MeOH, 20:1, v/v). IR (ATR): 3277, 3055, 2928, 2853, 2803, 1466, 1437, 1323, 1154, 1130, 1074, 1027, 951, 860, 816, 747 cm^-1^. ^1^H-NMR (400.130 MHz, CDCl_3_): δ = 0.95–1.05 (1 H, m), 1.43–1.53 (1 H, m), 1.58–1.66 (2 H, m), 1.73–1.80 (1 H, m), 1.88 (1 H, t, *J =* 8.4 Hz), 2.12 (1 H, t, *J =* 9.6 Hz), 2.59–2.69 (2 H, m), 2.67 (3 H, s), 2.84–2.95 (2 H, m), 3.49 (2 H, dd, *J_1_ =* 17.2 Hz, *J_2_* = 13.6 Hz), 5.59 (1 H, bs), 6.84 (1 H, s), 7.56–7.64 (2 H, m), 7.80 (1 H, dd, *J_1_* = 8.6 Hz, *J_2_* = 1.8 Hz), 7.87–7.89 (1 H, m), 7.91–7.94 (2 H, m), 8.40 (1 H, d, *J* = 1.2 Hz). ^13^C-NMR (100.613 MHz, CDCl_3_): δ = 19.12, 23.85, 27.85, 35.25, 46.73, 54.09, 56.91, 58.39, 115.53, 122.26, 127.41, 127.80, 128.20, 128.59, 129.12, 129.35, 132.05, 134.61, 136.80, 152.95, 165.99. HRMS (ESI+): *m/z* calcd for C_21_H_26_N_3_O_2_S_2_ [M+H]^+^ 416.1466; found 416.1457. HPLC purity, 98% at 254.16 nm (method C, *t*_R_ = 7.52 min).

***Synthesis of (±)-N-((1-(benzo[d]thiazol-2-yl)piperidin-3-yl)methyl)naphthalene-2-sulfonamide (71)***

Synthesized from (±)-(1-(benzo[*d*]thiazol-2-yl)piperidin-3-yl)methanamine 2,2,2-trifluoroacetate^14^ (**70**) and naphthalene-2-sulfonyl chloride via general procedure 1. Purified by flash column chromatography using DCM/MeOH (15:1, v/v) as the eluent to produce 0.062 g of **71** as a white solid (51% yield). *R*_f_ = 0.66 (DCM/MeOH, 15:1, v/v). IR (ATR): 2852, 1591, 1528, 1442, 1329, 1262, 1158, 1129, 1072, 825, 755, 655 cm^-1^. ^1^H-NMR (400.130 MHz, DMSO-*d6*): δ = 1.16–1.23 (1 H, m), 1.40–1.47 (1 H, m), 1.68–1.76 (3 H, m), 2.73–2.76 (2 H, m), 2.80–2.86 (1 H, m), 3.06–3.12 (1 H, m), 3.85–3.88 (1 H, m), 4.02–4.06 (1 H, m), 7.03–7.07 (1 H, m), 7.24–7.28 (1 H, m), 7.41–7.43 (1 H, m), 7.64–7.73 (3 H, m), 7.84 (1 H, dd, *J_1_*=8.8 Hz, *J_2_*=2.0 Hz), 7.91 (1 H, t, *J*=6.0 Hz), 8.03–8.05 (1 H, m), 8.13–8.17 (2 H, m), 8.45 (1 H, bs). ^13^C-NMR (100.613 MHz, DMSO-*d6*): δ = 23.70, 27.48, 35.56, 45.52, 48.70, 51.77, 118.29, 120.94, 121.00, 122.16, 125.84, 127.26, 127.50, 127.76, 128.62, 129.14, 129.38, 130.25, 131.69, 134.07, 137.32, 152.49, 167.88. HRMS (ESI+): *m/z* calcd for C_23_H_24_N_3_O_2_S_2_ [M+H]^+^ 438.1310; found 438.1317. HPLC purity, 95% at 254.16 nm (method C, *t*_R_ = 10.16 min).

***Synthesis of (±)-N-((1-(4-fluorobenzyl)piperidin-3-yl)methyl)-N-methylnaphthalene-2-sulfonamide*** ***(72)***

To a 25-mL round-bottomed flask equipped with a stirring bar, **68** (0.025 g, 0.061 mmol, 1.0 equiv.) and CH_3_CN (5 mL) were added, followed by Cs_2_CO_3_ (0.060 g, 0.183 mmol, 3.0 equiv.) and MeI (0.006 mL, 0.092 mmol, 1.5 equiv.). After stirring at 60 °C for 1 h, the solvent was evaporated and saturated aqueous NaHCO_3_ solution (10 mL) was added. The precipitated solid was filtered off, washed with H_2_O (20 mL) and air dried. The residue was purified by flash column chromatography using DCM/MeOH (20:1, v/v) as the eluent to obtain 0.014 g of **72** as a orange oil (54% yield). *R*_f_ = 0.43 (DCM/MeOH, 9:1, v/v). IR (ATR): 2934, 2854, 2796, 1602, 1507, 1455, 1336, 1271, 1218, 1155, 1130, 1096, 1072, 958, 889, 856, 818, 749 cm^-1^. ^1^H-NMR (400.130 MHz, CDCl_3_): *δ* = 1.00–1.09 (1 H, m), 1.51–1.59 (1 H, m), 1.67–1.76 (2 H, m), 1.85 (1 H, t, *J =* 10.0 Hz), 1.90–1.95 (1 H, m), 2.00 (1 H, t, *J =* 10.0 Hz), 2.70–2.76 (1 H, m), 2.74 (3 H, s), 2.83–2.87 (1 H, m), 2.91 (2 H, d, *J =* 8.0 Hz), 3.42 (1 H, d, *J =* 13.2 Hz), 3.53 (1 H, d, *J =* 12.4 Hz), 6.98 (2 H, tt, *J_1_* = 8.6 Hz, *J_2_* = 2.3 Hz), 7.27 (2 H, d, *J* = 7.0 Hz), 7.61–7.67 (2 H, m), 7.16 (1 H, dd, *J_1_* = 8.4 Hz, *J_2_* = 2.0 Hz), 7.91–7.93 (1 H, m), 7.95–7.99 (2 H, m), 8.34 (1 H, d, *J* = 1.6 Hz). ^13^C-NMR (100.613 MHz, MeOD): *δ* = 25.53, 29.32, 35.50, 36.06, 54.90, 55.26, 58.76, 63.60, 116.00 (d, *^2^J =* 21.1 Hz), 123.91, 128.88, 129.14, 129.82, 130.10, 130.44, 130.65, 132.67 (d, *^3^J =* 8.1 Hz), 133.81, 134.52 (d, *^4^J =* 3.7 Hz), 135.92, 136.40, 163.69 (d, *^1^J =* 242.0 Hz). HRMS (ESI+): *m/z* calcd for C_24_H_28_N_2_O_2_SF [M+H]^+^ 427.1856; found 427.1852. HPLC purity, 97% at 254.16 nm (method C, *t*_R_ = 13.96 min).

***Synthesis of tert-butyl 2-methoxyethyl(piperidin-4-ylmethyl)carbamate (78)***

Synthesized from *tert*-butyl (1-benzylpiperidin-4-yl)methyl(2-methoxyethyl)carbamate^10^ (**76**) via general procedure 8 to produce 1.696 g of **78** as a slightly golden oil (96% yield). *R*_f_ = 0.57 (DCM/MeOH/Et_3_N, 20:2:1, v/v). HRMS (ESI+): *m/z* calcd for C_14_H_29_N_2_O_3_ [M+H]^+^ 273.2178; found 273.2172.

***Synthesis of tert-butyl 3-methoxypropyl(piperidin-4-ylmethyl)carbamate (79)***

Synthesized from *tert*-butyl (1-benzylpiperidin-4-yl)methyl(3-methoxypropyl)carbamate^10^ (**77**) via general procedure 8 to produce 2.169 g of **79** as a slightly golden oil (94% yield). *R*_f_ = 0.56 (DCM/MeOH/Et_3_N, 20:2:1, v/v). HRMS (ESI+): *m/z* calcd for C_15_H_31_N_2_O_3_ [M+H]^+^ 287.2335; found 287.2331.

***Synthesis of tert-butyl (1-(2,3-dihydro-1H-inden-2-yl)piperidin-4-yl)methyl(2-methoxyethyl)carbamate (80)***

Synthesized from *tert*-butyl 2-methoxyethyl(piperidin-4-ylmethyl)carbamate (**78**) and 1*H*-inden-2(3*H*)-one via general procedure 9. Purified by flash column chromatography using DCM/MeOH (20:1, v/v) as the eluent, to produce 0.198 g of **80** as a slightly yellow solid (59% yield). *R*_f_ = 0.47 (DCM/MeOH, 10:1, v/v). IR (KBr): 2930, 2808, 1688, 1475, 1407, 1282, 1149, 1113, 1012, 979, 871, 745, 664cm^-1^. ^1^H-NMR (400.130 MHz, CDCl_3_): δ = 1.25–1.45 (11 H, m), 1.68 (3 H, d, *J* = 12.3 Hz), 1.95–2.06 (2 H, m), 2.88–2.93 (2 H, m), 3.05–3.17 (7 H, m), 3.34–3.39 (5 H, m), 3.44–3.53 (2 H, m), 7.11–7.18 (4 H, m). ^13^C-NMR (100.613 MHz, CDCl_3_): δ = 28.35, 29.82, 35.08, 35.39, 37.12, 46.92, 47.93, 51.48, 51.73, 53.54, 58.73, 67.11, 70.91, 71.08, 79.27, 79.37, 124.24, 126.24, 141.49, 155.53, 155.69. HRMS (ESI+): *m/z* calcd for C_23_H_37_N_2_O_3_ [M+H]^+^ 389.2804; found 389.2810. HPLC purity, 98% at 254.16 nm (method C, *t*_R_ = 11.87 min).

***Synthesis of tert-butyl (1-(2,3-dihydro-1H-inden-2-yl)piperidin-4-yl)methyl(3-methoxypropyl)carbamate* *(81)***

Synthesized from *tert*-butyl 3-methoxypropyl(piperidin-4-ylmethyl)carbamate (**79**) and 1*H*-inden-2(3*H*)-one via general procedure 9. Purified by flash column chromatography using DCM/MeOH (20:1, v/v) as the eluent, to produce 0.188 g of **81** as an off-white solid (67% yield). *R*_f_ = 0.36 (DCM/MeOH, 9:1, v/v). IR (KBr): 2954, 2930, 2791, 2748, 1694, 1478, 1412, 1387, 1364, 1348, 1318, 1284, 1272, 1256, 1232, 1135, 1118, 1027, 974, 909, 888, 867, 838, 757 cm^-1^. ^1^H-NMR (400.130 MHz, CDCl_3_): δ = 1.30–1.37 (2 H, m), 1.44 (9 H, s), 1.56–1.71 (3 H, m), 1.77–1.83 (2 H, m), 1.96–2.10 (2 H, m), 2.90–2.95 (2 H, m), 3.03–3.10 (6 H, m), 3.15–3.19 (1 H, m), 3.22–3.28 (2 H, m), 3.32 (3 H, s), 3.35–3.39 (2 H, m), 7.11–7.14 (2 H, m), 7.15–7.19 (2 H, m). ^13^C-NMR (100.613 MHz, CDCl_3_): δ = 28.19, 28.45, 28.87, 29.87, 35.02, 35.46, 37.13, 44.68, 45.80, 51.48, 51.73, 52.78, 58.59, 67.14, 70.12, 70.36, 79.27, 124.32, 126.36, 141.51, 155.73. HRMS (ESI+): *m/z* calcd for C_24_H_39_N_2_O_3_ [M+H]^+^ 403.2961; found 403.2964. HPLC purity, 95% at 254.16 nm (method C, *t*_R_ = 12.89 min).

***Synthesis of N-((1-(2,3-dihydro-1H-inden-2-yl)piperidin-4-yl)methyl)-2-methoxyethanamine di(2,2,2-trifluoroacetate) (82)***

Synthesized from *tert*-butyl (1-(2,3-dihydro-1*H*-inden-2-yl)piperidin-4-yl)methyl(2-methoxyethyl)carbamate (**80**) via general procedure 5 to produce 0.141 g of **82** as a white solid (85% yield). HRMS (ESI+): *m/z* calcd for C_18_H_29_N_2_O [M+H]^+^ 289.2280; found 289.2286.

***Synthesis of N-((1-(2,3-dihydro-1H-inden-2-yl)piperidin-4-yl)methyl)-3-methoxypropan-1-amine di(2,2,2-trifluoroacetate) (83)***

Synthesized from *tert*-butyl (1-(2,3-dihydro-1*H*-inden-2-yl)piperidin-4-yl)methyl(3-methoxypropyl)carbamate (**81**) to produce 0.198 g of **83** as a white solid (88% yield). HRMS (ESI+): *m/z* calcd for C_19_H_31_N_2_O [M+H]^+^ 303.2436; found 303.2433.

***Synthesis of N-((1-(2,3-dihydro-1H-inden-2-yl)piperidin-4-yl)methyl)-N-(2-methoxyethyl)naphthalene-2-sulfonamide (84)***

Synthesized from *N*-((1-(2,3-dihydro-1*H*-inden-2-yl)piperidin-4-yl)methyl)-2-methoxyethanamine di(2,2,2-trifluoroacetate) (**82**) and naphthalene-2-sulfonyl chloride via general procedure 1. Purified by precipitation from EtOAc by adding *n*-hexane to produce 0.052 g of **84** as a white solid (75% yield). *R*_f_ = 0.48 (DCM/MeOH, 10:1, v/v). IR (ATR) = 2937, 1672, 1329, 1153, 1129, 1073, 999, 820, 743, 652, 617 cm^-1^. ^1^H-NMR (400.130 MHz, CDCl_3_): δ = 1.57 (2 H, bs), 1.89–1.92 (3 H, m), 2.36 (2 H, bs), 3.09–3.34 (13 H, m), 3.48 (3 H, t, *J* = 5.5 Hz), 7.14–7.20 (4 H, m), 7.60–7.67 (2 H, m), 7.75–7.79 (1 H, m), 7.89–7.98 (3 H, m), 8.37 (1 H, s). ^13^C-NMR (400.130 MHz, CDCl_3_): δ = 28.35, 34.30, 36.06, 49.00, 50.98, 54.79, 58.68, 66.63, 71.31, 122.48, 124.31, 126.78, 127.53, 127.82, 128.49, 128.71, 129.14, 129.29, 132.08, 134.67, 135.97, 140.33. HRMS (ESI+): *m/z* calcd for C_28_H_35_N_2_O_3_S [M+H]^+^ 479.2368; found 479.2358. HPLC purity, 96% at 254.16 nm (method C, *t*_R_ = 13.42 min).

***Synthesis of N-((1-benzylpiperidin-4-yl)methyl)-N-(3-methoxypropyl)naphthalene-2-sulfonamide (6)***

Synthesized from *N*-((1-benzylpiperidin-4-yl)methyl)-3-methoxypropan-1-amine^10^ (**12**) and naphthalene-2-sulfonyl chloride via general procedure 1. Purified by flash column chromatography using DCM/MeOH (32:1, v/v) as the eluent to produce 0.254 g of **6** as a colorless oil that solidified into a white solid after cooling (86% yield). *R*_f_ = 0.37 (DCM/MeOH, 15:1, v/v). IR (ATR): 2921, 2804, 2757, 1452, 1334, 1267, 1196, 1152, 1114, 1073, 990, 880, 858, 735, 699, 651, 615 cm^-1^. ^1^H-NMR (400.130 MHz, CDCl_3_): δ = 1.24–1.33 (2 H, m), 1.64–1.71 (3 H, m), 1.76–1.83 (2 H, m), 1.93–1.99 (2 H, m), 2.89 (2 H, d, *J* = 10.2 Hz), 3.04 (2 H, d, *J* = 6.9 Hz), 3.23–3.26 (5 H, m), 3.34 (2 H, t, *J* = 6.0 Hz), 3.51 (2 H, s), 7.23–7.31 (5 H, m), 7.59–7.66 (2 H, m), 7.77 (1 H, dd, *J_1_* = 8.6 Hz, *J_2_* = 1.8 Hz), 7.89–7.98 (3 H, m), 8.37 (1 H, d, *J* = 1.5 Hz). ^13^C-NMR (100.613 MHz, CDCl_3_): δ = 29.04, 29.92, 34.81, 46.60, 53.11, 54.63, 58.51, 63.12, 69.73, 122.50, 126.84, 127.38, 127.76, 128.05, 128.34, 128.52, 129.04, 129.08, 129.19, 132.06, 134.55, 136.34, 138.23. HRMS (ESI+): *m/z* calcd for C_27_H_35_N_2_O_3_S [M+H]^+^ 467.2368; found 467.2359. HPLC purity, 99% at 254.16 nm (method C, *t*_R_ = 13.71 min).

***Synthesis of N-((1-benzylpiperidin-4-yl)methyl)-N-(2-methoxyethyl)naphthalene-2-sulfonamide (75)***

Synthesized from *N*-((1-benzylpiperidin-4-yl)methyl)-2-methoxyethanamine^10^ (**74**) and naphthalene-2-sulfonyl chloride via general procedure 1. Purified by flash column chromatography using DCM/MeOH (32:1, v/v) as the eluent to produce 0.257 g of **75** as a colorless oil that solidified into a white solid after cooling (88% yield). *R*_f_ = 0.35 (DCM/MeOH, 15:1, v/v). IR (ATR): 2921, 1451, 1333, 1265, 1151, 1116, 1073, 986, 816, 732, 699, 615 cm^-1^. ^1^H-NMR (400.130 MHz, CDCl_3_): δ = 1.22–1.32 (2 H, m), 1.68–1.71 (3 H, m), 1.95 (2 H, t, *J* = 11.4 Hz), 2.89 (2 H, d, *J* = 11.2 Hz), 3.09 (2 H, d, *J* = 6.9 Hz), 3.23 (3 H, s), 3.33 (2 H, t, *J* = 6.0 Hz), 3.48–3.51 (4 H, m), 7.22–7.31 (5 H, m), 7.59–7.66 (2 H, m), 7.78 (1 H, dd, *J_1_* = 8.7 Hz, *J_2_* = 1.82 Hz), 7.89–7.97 (3 H, m), 8.38 (1 H, d, *J* = 1.6 Hz). ^13^C-NMR (100.613 MHz, CDCl_3_): δ = 29.82, 34.80, 48.39, 53.19, 55.23, 58.65, 63.15, 71.26, 122.52, 126.85, 127.40, 127.40, 127.77, 128.06, 128.37, 128.55, 129.05, 129.15, 132.05, 134.58, 136.39, 138.29. HRMS (ESI+): *m/z* calcd for C_26_H_33_N_2_O_3_S [M+H]^+^ 453.2212; found 453.2219. HPLC purity, 99% at 254.16 nm (method C, *t*_R_ = 13.13 min).

***Synthesis of N-((1-benzylpiperidin-4-yl)methyl)naphthalene-2-sulfonamide (86)***

Synthesized from (1-benzylpiperidin-4-yl)methanamine^10^ (**87**) and naphthalene-2-sulfonyl chloride via general procedure 1. Purified by flash column chromatography using DCM/MeOH (20:1, v/v) as the eluent to produce 0.239 g of **86** as a colorless oil that solidified into a white solid after cooling (73% yield). *R*_f_ = 0.31 (DCM/MeOH, 15:1, v/v). IR (ATR): 3246, 2930, 1435, 1314, 1151, 1127, 1076, 1049, 1002, 868, 827, 808, 743, 698, 641, 606 cm^-1^. ^1^H-NMR (400.130 MHz, CDCl_3_): δ = 1.15–1.25 (2 H, m), 1.41–1.52 (1 H, m), 1.64 (2 H, d, *J* = 12.5 Hz), 1.89 (2 H, t, *J* = 11.1 Hz), 2.82–2.86 (4 H, m), 3.46 (2 H, s), 4.72–4.75 (1 H, m), 7.21–7.31 (5 H, m), 7.59–7.67 (2 H, m), 7.83 (1 H, dd, *J_1_* = 8.7 Hz, *J_2_* = 1.9 Hz), 7.90–7.92 (1 H, m), 7.95–7.97 (2 H, m), 8.43 (1 H, d, *J* = 1.4 Hz). ^13^C-NMR (100.613 MHz, CDCl_3_): δ = 29.58, 35.95, 48.65, 52.97, 63.10, 122.19, 126.85, 127.44, 127.88, 128.05, 128.29, 128.65, 129.04, 129.12, 129.43, 132.02, 134.65, 136.59, 138.17. HRMS (ESI+): *m/z* calcd for C_23_H_27_N_2_O_2_S [M+H]^+^ 395.1793; found 395.1796. HPLC purity, 99% at 254.16 nm (method C, *t*_R_ = 9.78 min).

***Synthesis of N-((1-(2,3-dihydro-1H-inden-2-yl)piperidin-4-yl)methyl)-N-(3-methoxypropyl)naphthalene-2-sulfonamide* *(85)***

Synthesized from *N*-((1-(2,3-dihydro-1*H*-inden-2-yl)piperidin-4-yl)methyl)-3-methoxypropan-1-amine di(2,2,2-trifluoroacetate) (**83**) and naphthalene-2-sulfonyl chloride via general procedure 1. Purified by flash column chromatography using DCM/MeOH (20:1, v/v) as the eluent, to produce 0.053 g of **85** as a white solid (59% yield). *R*_f_ = 0.29 (DCM/MeOH, 20:1, v/v). IR (KBr): 2936, 2853, 2804, 2759, 1639, 1458, 1349, 1330, 1255, 1200, 1154, 1129, 1071, 1002, 881, 827, 776, 738 cm^-1^. ^1^H-NMR (400.130 MHz, CDCl_3_): δ = 1.28 (2 H, qd, *J_1_ =* 13.0 Hz, *J_2_* = 3.2 Hz), 1.74–1.79 (3 H, m), 1.80–1.85 (2 H, m), 2.02 (2 H, t, *J =* 11.2 Hz), 2.85–2.91 (2 H, m), 2.99–3.09 (6 H, m), 3.11–3.20 (1 H, m), 3.23–3.27 (2 H, m), 3.27 (3 H, s), 3.36 (2 H, t, *J =* 6.0 Hz), 7.11–7.18 (4 H, m), 7.59–7.66 (2 H, m), 7.77 (1 H, dd, *J_1_ =* 8.8 Hz, *J_2_* = 1.8 Hz) 7.90–7.92 (1 H, m), 7.94–7.98 (2 H, m), 8.38 (1 H, d, *J =* 1.2 Hz). ^13^C-NMR (100.613 MHz, CDCl_3_): δ = 29.13, 29.78, 34.83, 37.06, 46.69, 51.38, 54.56, 58.61, 67.01, 69.80, 122.58, 124.32, 126.37, 127.46, 127.83, 128.48, 128.62, 129.18, 129.27, 132.14, 134.65, 136.33, 141.46. HRMS (ESI+): *m/z* calcd for C_29_H_37_N_2_O_3_S [M+H]^+^ 493.2525; found 493.2534. HPLC purity, 99% at 254.16 nm (method G, *t*_R_ = 13.01 min).

***Synthesis of (±)-N-((1-benzylpiperidin-3-yl)methyl)-N-(2-methoxyethyl)naphthalene-2-sulfonamide hydrochloride (2 hydrochloride)***

To a 100-mL round-bottomed flask equipped with a stirring bar, **2** (0.993 g, 2.194 mmol, 1.0 equiv.) and Et_2_O (50 mL) were added at room temperature. The resulting suspension was stirred and enough 1,4-dioxane (10 mL) was added to produce a clear solution from the suspension. The solution was agitated with a stream of argon for 15 min, and then cooled to 0 °C. Then a 4 M solution of HCl in 1,4-dioxane (1.100 mL, 4.388 mmol, 2.0 equiv.) was added drop-wise with a glass syringe. After 15 min, the reaction mixture was allowed to warm to room temperature and stirred for an additional 30 min. After evaporation of the solvent, Et_2_O (50 mL) was added to the oily residue, and the flask was placed in an ultrasonic bath until the oily residue transformed into a white solid. The flask was removed from the ultrasonic bath and the precipitate was allowed to settle to the bottom of the flask. The supernatant was removed, Et_2_O (50 mL) was added, and the flask was placed back in the ultrasonic bath for 1 min. This procedure was repeated three times and the solid residue was dried at reduced pressure, to produce 1.036 g of **2** hydrochloride as a white solid (97% yield). HPLC purity, 99% at 254.16 nm (method H, *t*_R_ = 18.85 min).

**8. SEPARATION OF COMPOUND 2 BY SEMI-PREPARATIVE REVERSE-PHASE CHIRLA HPLC**

The resolution of **2** was accomplished by 63 runs of method A. Eluates corresponding to the two chromatographic peaks were pooled and evaporated to about 5 mL, and then transferred to a 50-mL separating funnel. Water (6 mL) and saturated aqueous NaHCO_3_ solution (6 mL) were added and extracted with DCM (20 mL). The organic phase was dried over anhydrous Na_2_SO_4_, and evaporated, to produce 28.3 mg of the first-eluted enantiomer as a colorless oil, and 27.6 mg of the second-eluted enantiomer as a colorless oil. Analytical reversed-phase chiral HPLC analysis (method B, 254 nm) of both enantiomers gave an enantiomeric excess (e.e.) of 97%. The specific rotation of the first-eluted enantiomer was [α]_D_^23^ **+**8.68 (*c* 0.250, CHCl_3_), while the specific rotation of the second-eluted enantiomer was [α]_D_^23^ –7.97 (*c* 0.250, CHCl_3_). The analytical reversed-phase chiral HPLC retention times were (method F, 254 nm): **(+)-2**, 30.57 min; **(–)-2**, 34.17 min.

**9. *IN-VITRO* INHIBITORY ACTIVITY AND IC_50_ CALCULATIONS**

5,5′-Dithiobis (2-nitrobenzoic acid) (Ellman’s reagent; DTNB), and the butyrylthiocholine and acetylthiocholine iodides were purchased from Sigma-Aldrich (Steinheim, Germany). mAChE and recombinant huBChE at the stock concentration of 4.6 mg mL^–1^ in 10 mM MES buffer (pH 6.5) were kindly donated by Florian Nachon (IBS, Grenoble). The enzyme solutions were prepared by dilution of the concentrated stocks in phosphate-buffered solution (0.1 M, pH 8.0). The reactions were carried out in a final volume of 300 μL of 0.1 M phosphate-buffered solution, pH 8.0, containing 333 μM DTNB, 5 ×10^–4^ M butyrylthiocholine/ acetylthiocholine and 1 ×10^–9^ M or 5 ×10^–11^ M huBChE or mAChE, respectively. The reactions were started by addition of the substrate, at room temperature. The final content of the organic solvent (DMSO) was always 1%. The formation of the yellow 5-thio-2-nitrobenzoate anion as a result of the reaction of DTNB with the thiocholines was monitored for 1 min as the change in absorbance at 412 nm, using a 96-well microplate reader (Synergy™ H4; BioTek Instruments, Inc., USA). To determine the blank value (b), phosphate-buffered solution replaced the enzyme solution. The initial velocity (v_0_) was calculated from the slope of the linear trend obtained, with each measurement carried out in triplicate. For the first inhibitory screening, stock solutions of the test compounds (1 mM) were prepared in DMSO. The compounds were added to each well at a final concentration of 10 μM. The reactions were started by addition of the substrate to the enzyme and inhibitor that had been preincubated for 300 s, to allow complete equilibration of the enzyme–inhibitor complexes. The initial velocities in the presence of the test compounds (v_i_) were calculated. The inhibitory potencies are expressed as the residual activities (RA = (v_i_ – b) / (v_o_ – b)). For the IC_50_ measurements, eight different concentrations of each compound were used to obtain enzyme activities of between 5% and 90%. The IC_50_ values were obtained by plotting the residual enzyme activities against the applied inhibitor concentrations, with the experimental data fitted to Equation (1):

Y = Bottom + (Top – Bottom) / (1+10ˆ((LogIC_50_ – X) × HillSlope)) (1),

where X is the logarithm of the inhibitor concentration, and Y is the residual activity. For the fitting procedure, the Gnuplot software and an in-house python script were used.

**10. CRYSTALLIZATION, DATA COLLECTION, AND PROCESSING**

HuBChE from insect cells was concentrated to 6 mg mL**^–^**^1^ in 10 mM Tris, pH 7.4, and crystallized as previously described^15^. Briefly, crystals were grown at room temperature using the hanging-drop vapor diffusion method. The mother liquor solution was 0.2 M ammonium acetate, 12% polyethylene glycol 4000. Crystals were soaked for 1h in a solution of the mother liquor complemented with the investigated racemic compound (**2**, **7**, or **8**) at 100 µM. Prior to data collection, crystals were cryoprotected by a short soak in a mother liquor solution complemented with 18% glycerol, before being flash-cooled directly in a N_2_ gas stream at 100 K. Data were collected at the beamlines ID14-EH4 and ID23-2 of the European Synchrotron Radiation Facility (Grenoble, France) using a wavelength of 0.873 Å. Data were indexed and integrated using XDS^16^, and were scaled and merged with XSCALE and XDSCONV (Supplementary Table 3). The structure was solved using the molecular replacement method, using PHASER^17^. The search model was the huBChE model (PDB code 1POM), from which all ligands and sugars were removed. Reciprocal-space refinement was performed using Phenix^18^; briefly, initial rigid-body refinement and simulated annealing were followed by cycles of energy minimization, and grouped isotropic temperature factor refinement. Local noncrystallographic symmetry restraints between the two huBChE monomers in the asymmetric unit were applied during the refinement procedure, as well as the use of a reference model as prior structural knowledge^19^. The model refinement was interspersed with sessions of model rebuilding using the program Coot^20^. The ligand topology was generated with the PRODRG server^21^. The coordinates and structure factors have been deposited in the Protein Data Bank under accession codes 5DYW (compound **2**), 5DYY (compound **7**), and 5DYT (compound **8**).

**11. EXPERIMENTS IN RAT BRAIN SLICES**

***11.1. Cholinesterase histochemistry***

Compound **2** was used in the form of its hydrochloride salt. The modified method of Koelle and Friedenwald was used to obtain histochemical reaction products at sites of ChE enzyme activity^22^. Briefly, the sections described above were covered with 30 µL of the ‘Koelle’ solution (pH 5.0) containing 3.1 mM copper (II) sulfate, 10 mM glycine, 50 mM sodium acetate, with 4 mM of the ATCI/BTCI as the substrate for ChE. Then 10 mM BW-284C51 and 300 µM ethopropazine were added to the corresponding solutions to completely block AChE and BChE, respectively. The rat brain slices covered with the Koelle solution were incubated for 2 h at 37 °C in a humidity chamber, to prevent evaporation and desiccation of sections. Following the enzymatic incubation, the sections were rinsed (3× 3 min) in phosphate-buffered saline with potassium (pH 7.4), and placed for 1 min in a developer of 1% sodium sulfide (Na_2_S.9H_2_O) in distilled water (pH adjusted to 7.5 with glacial acetic acid). Next, the sections were rinsed (3× 3 min) in distilled water, and the reaction product was intensified for 30 s in 1% silver nitrate solution in distilled water. The staining reaction was terminated by rinses in distilled water (2× 3 min) and a final rinse in phosphate-buffered saline with potassium (3 min). Finally, the sections were dehydrated through a graded ethanol series (50%, 70%, 95%, 100%; 3 min in each), cleared with xylene (3 min), and covered with a cover-slip with DPX Mountant for histology.

For histochemical determination of BChE inhibition with rat brain slices by sulfonamide **2**, the section with the highest BChE activity was used. The section was prepared and fixed as described above, with increasing concentrations of compound **2** added to the Koelle solution, to the desired final concentrations (1-300 µM).

***11.2. Analysis of butyrylcholinesterase stained structures***

Stained sections were subjected to extensive microscopic examination and cytoarchitectonic location. Images were captured using a CoolSNAP camera (Photometrics, Tuscon, Arizona, USA) and a Northern Light box (Model B90; Imaging Research, St. Cathrines, Ontario, Canada). Each brain section was digitalized to produce a high resolution, 256 level, grayscale image. A semi-quantitative measure of ChE enzyme activity, the relative optical density score of staining intensity, was determined using the MCID Elite 6.0 software (Imaging Research, St. Catherines, Ontario, Canada).

**12. CELL BASED ASSAYS**

***12.1. Metabolic activity assay***

HepG2 cells (1 ×10^4^ cells/well) were treated for 24 h with the appropriate concentrations of the compounds of interest or the corresponding vehicle (control cells), in triplicates in 96-well plates. The metabolic activities were determined using the CellTiter 96 Aqueous One Solution Cell Proliferation Assay (Promega, WI/USA), according to the manufacturer instructions.

***12.2. Cell viability assay***

SH-SY5Y cells were seeded in 96-well plates (2 ×10^4^/well) and assessed in the **MTS (**[3-(4,5-dimethylthiazol-2-yl)-5-(3-carboxymethoxyphenyl)-2-(4-sulfophenyl)-2H-tetrazolium, inner salt) **assay for their response to treatments with compound **2**.** The cells were treated as described above, and cell viability was assessed after 48 h using the **CellTiter 96 Aqueous One Solution Cell Proliferation Assay (Promega, Madison, WI, USA), according to the manufacturer instructions. Absorbance was measured with an automatic microplate reader (Tecan Safire^2^, Switzerland) at a wavelength of 492 nm. The data are presented as percentages of the control (DMSO).**

***12.3. Assessment of cytotoxicity***

The neuroprotective effect of compound **2** on cytotoxic effect of Aβ_(1–42)_ was assessed by flow cytometry analysis using propidium iodide (PI; Sigma, St. Louis, MO, USA). PI does not cross cell membrane but stains DNA in cells when cell membrane is disintegrated. SH-SY5Y cells were seeded into a 24-well culture plate (1.2 ×10^5^/well) and next day, treated as described above. After 48 h treatment, cells were washed with pre-warmed PBS and further stained with PI solution (30 µM) for 15 min at 37 ºC. Cells were then analyzed for cytotoxicity by flow cytometry on FACS Calibur (BD Bioscience, San Jose, CA, USA). The percentage of PI positive cells (PI^pos^) was evaluated using FlowJo software (Ashland, OR, USA) and results are presented as a relative fold increase of PI^pos^ corresponding vehicle treatment (DMSO control).

**13. ASSESSMENT OF Aβ**_1–42_ **AGGREGATION INHIBITORY ACTIVITY**

***13.1. Thioflavin-T (ThT) fluorometric assay***^23^

Recombinant human HFIP-pretreated Aβ_1–42_ peptide (Merck Millipore, Darmstadt, Germany) was dissolved in DMSO to give 75 μM stock solution. The stock solution was further diluted in HEPES buffered solution (150 mM HEPES, pH 7.4, 150 mM NaCl), to 7.5 μM. Aβ_1–42_ solution was then added to the compound **2** in black-walled 96-well plate, and diluted with ThT solution (final concentration of 10 μM). Final mixture contained 1.5 μM Aβ_1–42_, 10 μM of compound **2**, and 3% DMSO. ThT fluorescence was measured every 300 s (excitation wavelength of 440 nm, emission wavelength of 490 nm), with the medium continuously shaking between measurements using a 96-well microplate reader (Synergy™ H4, BioTek Instruments, Inc., USA). The ThT emission of the Aβ_1–42_ began to rise after 4 h, reached a plateau after 20 h, and remained almost unchanged for an additional 28 h of incubation. The fluorescence intensities at the plateau in the absence and presence of the test compound were averaged, and the average fluorescence of the corresponding wells at t= 0 h was subtracted. The Aβ_1–42_ aggregation inhibitory potency is expressed as the percentage inhibition (% inh= (1 – F*_i_* / F*_0_*) ×100%), where F*_i_* is the increase in fluorescence of Aβ_1–42_ treated with the test compound, and F*_0_* is the increase in fluorescence of Aβ_1–42_ alone.

***13.2. Dot-blot assay***

Recombinant human HFIP-pretreated Aβ_1–42_ peptide (Merck Millipore, Darmstadt, Germany) was dissolved in DMSO to give 100 μM stock solution. Prior to the incubations, the Aβ_1–42_ peptide stock solution was diluted in HEPES buffered solution (150 mM HEPES, pH 7.4, 150 mM NaCl) to give 10 μM Aβ_1–42_. Then 10 μL of this Aβ_1–42_ solution was mixed with 10 µL of the compound **2** (100 μM stock in HEPES buffered solution; 10 μM final concentration), and added to the black-walled 96-well plates in quadruplicate, and diluted with HEPES buffered solution to a final volume of 100 μL. To determine the blank value (*b*), 1% DMSO in HEPES buffered solution replaced the Aβ_1–42_ peptide solution. The sealed 96-well plate was placed in an incubator at 37 °C with continuous shaking, for 24 h. Then 10 μL from each well were spotted on nitrocellulose membrane. The membrane was dried and blocked with 5% non-fat milk in TBST (50 mM Tris, pH 7.4, 150 mM NaCl, 0.1% Tween 20) at room temperature for 30 min, washed with TBST, and probed with the LOC anti-amyloid fibrils antibody (Millipore, 1:10,000, in 2% non-fat milk-TBST) or the OC anti-amyloid fibrils antibody (Millipore, 1:10,000, in 2% non-fat milk-TBST), for 1 h. After washing with TBST, the nitrocellulose membrane was probed with horse-radish-peroxidase–conjugated anti-rabbit IgG (Millipore, 1:10,000), for 1 h. The blots were developed with Pierce ECL Western Blotting Substrate (Thermo Scientific) for 2 min, detected with a Bio-Rad ChemiDoc MP Imaging System, and further analyzed with Image Lab software (Bio-Rad). Inhibition of the Aβ_1–42_ aggregation was quantified using equation:

% inh= 1 – (R*_i_* – *b*) / (R*_0_* – *b*)) ×100% (2), where R*_i_* represents the response of Aβ_1–42_ treated with the compound **2**, R*_0_* the response of Aβ_1–42_ alone, and *b* the blank value.

**14. *IN-VITRO* PHARMACOKINETICS**

***14.1. In-vitro permeability assay with Caco2 cells***

Caco-2 cells were obtained from American Tissue Culture Collection (ATCC) HTB.37, lot 61777387 and were used in reported experiments within one year. They were grown on Transwell® culture inserts with a polycarbonate membrane (diameter, 12 mm; pore size, 0.4 μm). 50,000 cells/filter membranes were used for seeding and the medium was changed every two days. At day 18, transepithelial electrical resistance (TEER) was measured for each filter with Caco-2 cell monolayers. If the TEER values were in the range of 450–750 ×cm^2^, the Caco-2 cell monolayers were used for the subsequent testing of permeability at day 21. *ABCB1* activity was confirmed with Rhodamine123 (Sigma Aldrich, Germany) a marker substrate of *ABCB1*, which had an efflux ratio of 7.3 (basolateral-to-apical apparent permeability coefficient was 7.7×10^-6^ cm s^-1^ and apical-to-basolateral apparent permeability coefficient was 1.1×10^-6^ cm s^-1^). After inhibition with a selective *ABCB1* inhibitor PSC833 (Tocris bioscience, UK) the efflux ratio was reduced to 2.5 (basolateral-to-apical apparent permeability coefficient was 2.5×10^-6^ cm s^-1^ and apical-to-basolateral apparent permeability coefficient was 1.0×10^-6^ cm s^-1^). Fluorescence measurements with excitation at 485 nm and emission at 520 nm were used to quantify Rhodamine123 in samples taken as described later for compound **2**. The samples were acidified by dilution with the same volume of 0.01M HCl before fluorescence measurements.

Ringer buffer with 10 mM d-glucose or 10 mM mannitol on apical and basolateral side of the tissue, respectively, was used as an incubation saline. The tissue was kept at 37°C in a carbogen (95% O_2_ and 5% CO_2_) atmosphere during the experiment. The experiment started by the addition of stock solutions of compound **2** and fluorescein in the donor compartment to provide final 100 μM donor concentration of compound **2** and 20 µM fluorescein. The stock solutions were added either to the apical or to the basolateral compartment to obtain bidirectional permeability measurements. Six samples were withdrawn in 20 min intervals. Each time 300 μl was taken from the 1500 µL basolateral acceptor compartment and 100 µL from the 500 µL apical acceptor compartment. These volumes were immediately replaced by the appropriate fresh incubation saline. The concentrations of compound 2 in the samples were determined by HPLC-UV immediately after the experiment. The analysis was performed on an Agilent 1100 system (degasser, binary pump, well-plate sampler, column thermostate and a diode-array detector) using a Zorbax Eclipse XDB-C18 column (4.6 x 75mm, 3.5µm) at 55°C and a mobile phase consisting of 63% diluted phosphoric acid at pH 2.5 and 37 % acetonitrile with a flow of 2.0 mL/min. The detection wavelength was 231 nm and the retention time was 1.38 min. All apparent permeability coefficients were calculated as described previously^24^.

***14.2. Plasma protein binding***

The equilibrium dialysis technique was used to separate the fraction of the bound to human plasma protein. The assay was performed in a 96-well format in a dialysis block constructed from Teflon to minimize the unspecific binding^25^. Plasma from human was used as the default protein containing matrix. Shortly, the protein matrix was spiked with the compound **2** at 10 µM (n = 2), with a final DMSO concentration of 1%. Acebutolol, quinidine and warfarin were tested in each assay as reference compounds, which yield protein binding that represent low, medium and high bindings to human plasma proteins, respectively. The dialysate compartment was loaded with the phosphate buffered saline (PBS, pH 7.4), and the sample side was loaded with equal volume of the spiked protein matrix. The dialysis plate was then sealed and incubated at 37 °C for 4 h. A control sample (n = 2) was prepared from the spiked protein matrix in the same manner as the assay sample (without dialysis). The control sample served as the basis for the recovery determination. After the incubation, samples were taken from each compartment, diluted with phosphate buffer followed by addition of acetonitrile and centrifugation. The supernatants were then used for HPLC-MS/MS analysis using selected reaction monitoring. The HPLC conditions consisted of a binary LC pump with autosampler, a C18 column (2 × 20 mm), and gradient elution. The peak areas of the compound **2** in the buffer and test samples were used to calculate percent binding and recovery according to the following equations:

Protein binding (%) = (Area_p_-Area_b_) / Area_p_ ×100 (3),

Recovery (%) = (Area_p_-Area_b_) / Area_c_ ×100 (4),

Area_p_ = peak area of the analyte in the protein matrix

Area_b_ = peak area of the analyte in the assay buffer

Area_c_ = peak area of the analyte in the control sample

The recovery determination serves as an indicator of reliability of the calculated protein binding value. Low recovery would indicate that the test compound is lost during the course of the assay, most likely due to non-specific binding or degradation.

***14.3. Plasma stability***

The stability of the compound **2** in human plasma was determined in a 96-well plate format^26^. Human plasma was pre-warmed at 37 °C water bath for 5 min, followed by addition of the compound **2** at 1 µM with a final DMSO concentration of 0.5%. Propoxycaine and propantheline were tested as reference compounds simultaneously in each assay. The incubation was performed in a 37 °C water bath for 2 h. An aliquot of the incubation mixture was transferred to acetonitrile at 0, 0.5, 1, 1.5 and 2 h, respectively. Samples were then mixed and centrifuged. Supernatants were used for HPLC-MS/MS analysis using selected reaction monitoring, and peak areas were recorded for each analyte. The HPLC conditions consisted of a binary LC pump with autosampler, a C18 column (2 × 20 mm), and gradient elution. The area of precursor compound remaining after each time points relative to the amount remaining at time zero, expressed as percent, is calculated. Subsequently, the half-life (t_1/2_) was estimated from the slope of the initial linear range of the logarithmic curve of the compound remaining (%) *versus* time, assuming first order kinetics.

***14.4. Metabolic stability***

The metabolic stability, expressed as the intrinsic clearance (Cl_int_) of the compound **2** using human cryopreserved hepatocytes, was determined in a 96-well plate format^27^. Cryopreserved hepatocytes (mixed gender and pool of 10 or more) were thawed, washed, and resuspended in Krebs-Heinslet buffer (pH 7.3). The reaction was initiated by adding test compound **2** at 1 µM (with a final DMSO concentration of 0.01%) into cell suspension (final cell density of 0.7 million viable cells per mL), and incubated for 0, 0.5, 1, 1.5 and 2 h, respectively, at 37 °C/ 5% CO_2_. The reaction was stopped by adding acetonitrile into the incubation mixture. Samples were then mixed, transferred completely to another 96-well plate, and centrifuged. Supernatants were used for HPLC-MS/MS analysis. As reference substances, four compounds were tested in each assay. Propranolol is relatively stable, whereas flurazepam, naloxone and 7-hydroxy-4-trifluoromethylcoumarin (HFC) are readily metabolized in human hepatocytes. In the HPLC-MS/MS analysis peak areas corresponding to the test compounds were recorded. Metabolic stability, expressed as percentage of the parent compound remaining, was calculated by comparing the peak area of the compound at the time point relative to that at time 0. The half-life (t_1/2_) was estimated from the slope of the initial linear range of the logarithmic curve of the compound remaining (%) *versus* time, assuming first order kinetics. The apparent intrinsic clearance (Cl_int_ in µL/min/million cells) was calculated from the half-life according to the following formula:

Cl_int_ = 0.693/ (t_1/2_ × million cells/µL) (5).

***14.5. Metabolite identification***

Principal Phase I (oxidative) and Phase II (conjugative) metabolites have been detected and characterized following previously described preocedure^27^. Briefly, the test compound **2** (10 µL) was incubated at 37 °C in cryopreserved human hepatocytes (0.7 million cells/mL) for 120 min. Samples were taken at time 0 and the end of the incubation (2 h), respectively. A control sample (no test compound added) was also prepared and incubated for the same period of time. After the reaction was stopped by addition of acetonitrile, the supernatants were subjected to HPLC-MS analysis. Separation of analytes was achieved using Acquity HSS T3 column (100 × 2.1 mm, 1.8 µ, Waters). The sample solutions-supernatants (5 µL) were injected and eluted at a flow rate of 0.5 mL min^–1^, using a linear gradient of mobile phase A (0.1% formic acid in water) and mobile phase B (0.1% formic acid in MeCN). The gradient for mobile phase B was: 0-1.0 min, 5%; 1.0-7.0 min, 5%-80%; 7.0-7.5 min, 80%; 7.5-8.0 min, 80%-5%; 8.0-10.0 min, 5%. The HPLC was interfaced to a Waters Xevo G2 QTof system using electrospray ionization (source temperature: 150 °C, desolvation temperature: 400 °C, desolvation gas 400 L/h; capillary: 0.75 kV; sampling cone voltage 50V; extraction cone voltage: 2.7 V). A full-scan analysis in positive mode was performed over a mass-to-charge range suitable to cover the expected Phase I and Phase II metabolic product of the test compound (200–800 Da). An MS^E^ acquisition method was used, which utilizes a high and low collision energy function such that precursor (MS) and product ion (MS/MS) information on all analytes in the sample is collected in a single analysis. Full-scan Total Ion Chromatograms (TIC) obtained from the time 0 and time 2 h sample, and also a blank matrix sample, was compared using the MetaboLynx software package of the MS system. With the application the sample data files were processed in an automated fashion to look for expected and unexpected metabolic products, and unique peaks in the incubation samples were directly compared to the control sample. Retention times and the respective MS and MS/MS spectra of the species detected in the incubated sample and not in time-zero or blank sample were identified and recorded.

**15. *IN-VIVO* BLOOD PLASMA–BRAIN DISTRIBUTION**

***15.1. LC-MS/MS analysis***

For quantification, the reconstituted samples were analyzed by UHPLC-MS/MS, which included a liquid cromatograph (Agilent 1290) and a triple quadrupole mass spectrometer (Agilent 6460) connected through the JetStream ESI ion source interface (Agilent Techologies, Santa Clara, USA). Chromatographic separation was performed on a C18 column (50 × 2.1 mm; 2.6 μm particles; Kinetex), with a C18 cartridge guard column (4 × 2.0 mm; Phenomenex, Torrance, USA) and kept at 50 °C. The injection volume was 0.50 μL. After each injection, the injection needle was washed for 6 s with 80% MeOH. Mobile phase A was 0.05% formic acid in water, and mobile phase B was MeCN. The flow rate was 0.65 mL/min with the following gradient (as %B): 0-0.25 min, 10%-15%; 0.25-0.5 min, 15%-28%; 0.5-1.0 min, 28%-45%; 1.0-1.25 min, 45%-55%; 1.25-2.0 min, 55%-65%; 2.0-2.1 min, 65%-10%. The total run time including re-equlibration was 2.8 min. The ion source parameters were set as follows: drying gas temperature, 275 °C; drying gas flow rate, 5 L min^–1^; nebulizer, 45 PSI (3.1 ×10^5^ Pa); sheath gas temperature, 320 °C; sheath gas flow, 11 L min^–1^; capillary entrance voltage, 4000 V; nozzle voltage, 1000V; and delta EMV, 200 V. MRM transitions and fragmentation parameters for analytes quantified by UHPLC-MS/MS are presented in Supplemental Table 1.

Supplemental Table 11. MRM transitions and fragmentation parameters for analytes quantified by UHPLC-MS/MS.

| **Analyte** | ***m/z* precursor ion** | ***m/z* product ion** | **Collision energy**  **(eV)** | **Fragmentor**  **Voltage**  **(V)** | **Ion polarity** |
| --- | --- | --- | --- | --- | --- |
| Donepezil | 380.2 |  |  |  |  |
| quantifier |  | 91 | 37 | 86 | + |
| qualifier |  | 65 | 97 |  |  |
| Compound **2** | 453.2 |  |  |  |  |
| quantifier |  | 91.1 | 61 | 210 | + |
| qualifier |  | 127 | 49 |  |  |
| Haloperidol | 376 | 165 | 24 | 130 | + |

The instrument control, data acquisition, and quantification were performed using the MassHunter Workstation software B.06.00 (Agilent Technologies, Torrance, USA). MRM chromatograms showing the signal-to-noise ratios are presented in Supplemental Figure 1.


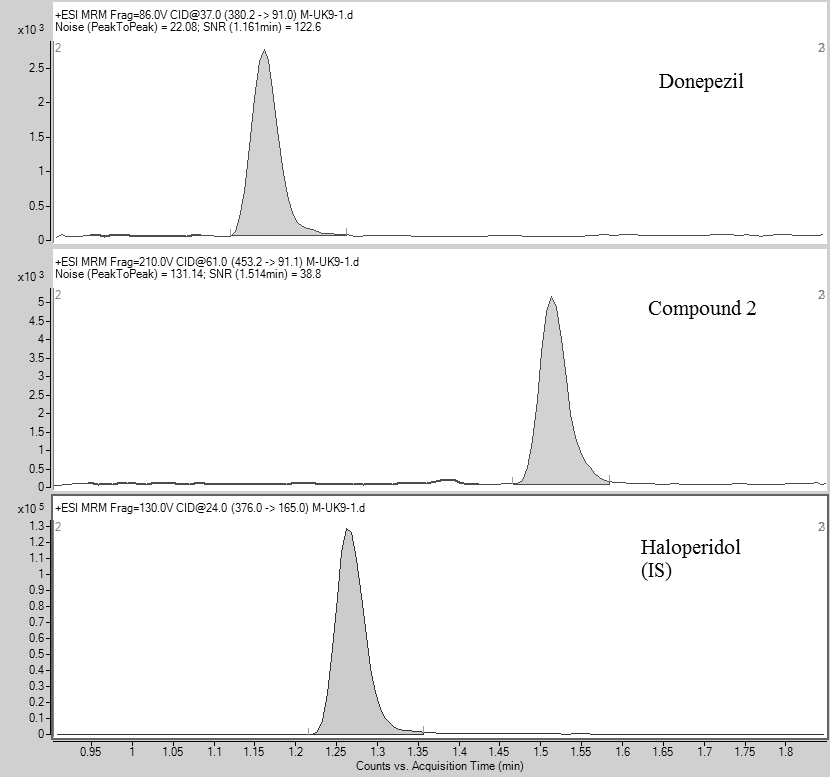


Supplemental Figure 11. MRM chromatograms of donepezil (top), compound 2 (middle) and haloperidol [internal standard (IS), (bottom trace)] in brain samples spiked at limits of quantification at 2.5 and 1.5 µg kg^–1^ for donepezil and compound 2, respectively. The signal to noise ratios (SNR) were above 30:1 as indicated on top pf each trace. The responses for brain study samples were at least 30 times higher.

***15.2. LC-MS/MS method validation***

The LC-MS/MS method was tested for accuracy, precision, linear range, limit of quantification, recovery, matrix effect, and stability, according to the FDA guidance for bioanalytical method validation^28^.

The method linear range was determined as the range of the calibration samples with acceptable accuracy (±15% bias) and precision (15% RSD). The calibration samples were prepared by spiking known amounts of standards dissolved in 80% methanol into blank blood plasma and blank brain tissue prior to the homogenization and extraction procedures. The added volume of the stock solution to 150 µL plasma or 150 mg brain tissue was from 10 μL to 150 μL at each of 10 levels, and the organic solvent addition was kept constant for all of the samples. The concentration ranges for donepezil and compound **2** in plasma were 2 μg L^–1^ to 300 μg L^–1^, and 4 μg L^–1^ to 600 μg L^–1^, respectively. The concentration range of donepezil and compound **2** in brain tissue was 1 μg kg^–1^ to 150 μg kg^–1^.The measured responses across the whole range were linear, and showed good correlation, with determination coefficients of 0.99 or greater.

The limit of quantification was set at the lowest calibration level that showed acceptable accuracy (bias < ±20%) and precision (<20% RSD) with the peak area at least five-fold greater than the response of the processed blank sample at the same retention time, and with a signal-to-noise ratio of at least 30:1 (root mean square). The determined limits of quantification for donepezil were 2.5 μg L^–1^ and 2.0 μg kg^–1^ in plasma and brain tissue, respectively. The limits of quantification for compound **2** were 1.5 μg L^–1^ and 10 μg kg^–1^ in plasma and brain tissue, respectively.

Quality control samples were prepared the same way as for the calibration, except with different weightings and at only three different levels: Low, medium and high (Supplemental Table 2).

Supplemental Table 12. Quality control levels for brain and plasma samples.

| **Quality control** | **Brain** | **Plasma** | |
| --- | --- | --- | --- |
| **level** | **donepezil and**  **compound 2 levels**  **(μg kg^–1^)** | **Donepezil levels**  **(μg L^–1^)** | **Compound 2 levels**  **(μg L^–1^)** |
| Low | 15 | 30 | 60 |
| Medium | 75 | 150 | 300 |
| High | 125 | 250 | 500 |

Accuracy was determined by comparing the measured concentrations of the quality control samples to their true values, and expressed as bias [%]. Method repeatability was determined by calculating the RSD from the separately prepared and analyzed quality control samples. These data are presented in Supplemental Tables 3 and 4.

Supplemental Table 13. Accuracy and precision data for donepezil in brain and plasma.

| **Quality control** | **Donepezil bias (%)** | | **Donepezil RSD (%)** | |
| --- | --- | --- | --- | --- |
| **level** | **Brain** | **Plasma** | **Brain** | **Plasma** |
| Low | -17.4 | 14.1 | 7.5 | 12.3 |
| Medium | 7.3 | -14.6 | 9.3 | 2.1 |
| High | -6.0 | -7.1 | 14.8 | 1.1 |

Supplemental Table 14. Accuracy and precision data for compound 2 in brain and plasma.

| **Quality control** | **Compound 2 bias (%)** | | **Compound 2 RSD (%)** | |
| --- | --- | --- | --- | --- |
| **level** | **Brain** | **Plasma** | **Brain** | **Plasma** |
| Low | -0.2 | -14.9 | 9.6 | 7.8 |
| Medium | 8.5 | -0.6 | 9.8 | 15.4 |
| High | -9.0 | 14.1 | 1.7 | 2.2 |

The extraction recovery, absolute matrix effect, and relative matrix effect were determined as reported29 at two quality control levels: low and high. Briefly, the recovery was calculated as the ratio between peak area response from the quality control samples spiked before extraction (A) and peak areas from samples spiked at the same levels after the extraction of blank samples (B), as in Equation (6):

Extraction recovery (%) = A/B ×100 (6).

The absolute matrix effect was calculated as a ratio between the peak area response from the quality control samples spiked before extraction (A) and the peak area response from the samples spiked with pure reconstitution solvent at the same levels (C), as in Equation (7):

Absolute matrix effect (%) = A/C ×100 (7).

The relative matrix effect was calculated as the RSD from the quality control samples spiked in blank matrices obtained from four different untreated donors. The recovery and matrix effect data are presented in Supplemental Table 5.

Supplemental Table 15. Data from the recovery and matrix effects for donepezil and compound 2.

| **Compound** | **Quality control** | **Extraction recovery**  **(%)** | | **Absolute matrix effect (%)** | | **Relative matrix effect (%)** | |
| --- | --- | --- | --- | --- | --- | --- | --- |
|  | **level** | **Brain** | **Plasma** | **Brain** | **Plasma** | **Brain** | **Plasma** |
| Donepezil | Low | 55.6 | 52.3 | 86.3 | 92.8 | 23.1 | 11.7 |
|  | High | 67.9 | 49.9 | 91.1 | 93.2 | 19.3 | 4.4 |
| Compound **2** | Low | 45.2 | 73.5 | 96.5 | 97.1 | 16.1 | 16.0 |
|  | High | 54.4 | 66.1 | 92.9 | 119.2 | 16.9 | 5.4 |

The stability experiments covered the following conditions: stock solution stability (refrigerator at 4 °C, 7 days), freeze/ thaw stability (4 cycles), short-term stability (4 h at 25 °C), long term stability (14 days at –20 °C), and autosampler stability (reconstituted samples, 48 h at 4 °C). The data showed no detectable mean analyte peak area deterioration compared to the time zero measurements (deviation <15% RSD). Indeed, in some samples, the signal responses were even higher at later time points, most probably due to solvent evaporation from closed vessels.

**16. BEHAVIORAL TESTING PARADIGMS**

***16.1. Passive avoidance task***

The effect of the test compounds on acquisition and retention in the passive avoidance task was conducted accordingly to a previously described method30. For this purpose, the passive avoidance apparatus was used (Panlab Harvard Apparatus, Spain). This consisted of a large white-painted illuminated compartment (26 × 26 × 34 cm) and a small black-painted dark compartment (13 × 7.5 × 7.5 cm) separated from each other by a guillotine door (5 × 5 cm).

To determine the effects of compound **2** and rivastigmine on scopolamine-induced memory impairment, the mice underwent two separate trials: an acquisition trial (the ‘conditioning phase’) and a retention trial (the ‘testing phase’). The testing phase was conducted 24 h after the acquisition phase. For the acquisition, each mouse was initially placed for 30 s in the light compartment (exploration period; guillotine gate closed). At the end of the exploration period the guillotine door between the light and the dark compartments was opened and the time elapsed before entering the black chamber was recorded. As soon as the mouse entered the dark compartment, the door automatically closed and an electrical shock (current intensity: 0.2 mA, duration: 2 s) was delivered through the grid floor.

For the retention trial (drug off), the mice were placed in the illuminated white compartment again, and the latency time between door opening and entry into the dark compartment was recorded for each mouse. If the mouse did not enter the dark compartment within 180 s (cut off latency), it was concluded that it remembered the foot shock from the acquisition trial. Better memory performance was indicated by longer latency before entry into the black chamber in the test (retention) phase than in the conditioning (acquisition) phase.

***16.2. Morris water maze test***

The Morris water maze is a circular, plastic, grey-painted pool (diameter, 120 cm; height, 60 cm), filled with water (up to about 48 cm below the edge, to prevent an animal from jumping out) that was maintained at 23 ±1 °C. The pool was divided into four equal quadrants (compass locations: NE, NW, SE, SW) by a computerized video tracking system (SMART, ver. 3.0; Panlab, Spain). An escape platform (diameter, 11 cm; height, 47 cm) at a fixed location (the center of the NW quadrant; i.e., the target quadrant). The escape platform was made of transparent Plexiglas and was immersed 1 cm under the surface of water; this was invisible to the swimming mouse. The maze was lighted with the intensity of 45 lx.

During the spatial acquisition trial (six consecutive days), the mice were assigned to training sessions (four training sessions a day; 4 h apart) in which the mice were trained to escape from the water by reaching the hidden platform, the location of which could be identified using distal extra-maze cues (A4-sized sheets of black laminated paper with colored geometric symbols) attached to the room walls to provide navigation points31. Visual cues had different colors and dimensions, and were kept constant during the whole experiment31,32. The whole experiment was conducted by an experimenter who remained always stationary in a constant location, thus serving as an additional distal cue for the swimming mice. For each trial, the mouse was placed in the water starting from a different randomly chosen quadrant that did not contain the platform, whereas the platform was always positioned in the same place. If an animal did not find the hidden platform within 60 s, it was gently placed on the platform for 15 s. The time taken to reach the hidden platform (escape latency time), distance travelled to reach the platform, and mean speed were recorded in each experimental group and analyzed.

On the seventh day (24 h after the last training) the platform was removed from the pool and a probe trial was performed (without drug treatment). Each animal was released from a different start point and was allowed to swim for 60 s. If a mouse did not find the platform place within 60 s, it was given a latency score of 60 s32,33. Latency time to the first crossing of the former platform location (target zone), number of crossings of the target zone, time spent in the target NW quadrant, total distance, distance spent in NW quadrant, entries into the NW quadrant, and mean speed were measured31.

***16.3. Two-day radial-arm water maze***

C57BL/6J mice were subjected to the radial arm water maze paradigm34,35. The main part of the apparatus (Panlab Harvard Apparatus, Spain) consists of a 6-arm maze in a pool filled with water to approximately 10 cm from the top, to be high enough to cover the hidden platform by about 0.5 cm, but also low enough that the visible platform was not covered. The pool was regularly cleaned of any debris left by the mice. The temperature of the water was kept constant during testing (23 ±1 °C). The tester remained in the same position throughout the testing, thus providing an additional visual cue for the mice.

On day 1, 15 trials were run in five blocks of three. Each trial lasted up to 60 s. The start arm was varied for each trial, with the goal arm remaining constant for a given individual for both days^34,35^. For the first 12 trials, the platform was alternately visible, then hidden, and then hidden for the last three trials. On day 2, the mice (without drug treatment on this day) were run in the same manner as on day 1, but the platform was hidden for all of the trials. As errors have been reported to be the most sensitive measure in this assay34, the number of errors (i.e., incorrect arm entries made) was counted in a 60 s time-frame. Incorrect arm entries occurred when the mouse selected an arm that was not the goal arm. Entries into the goal arm were not counted as errors, even if the platform was not located. An entry was considered to occur when all four legs of the mice had entered the alley completely. A failure to select an arm after 15 s was counted as an error, and mice that failed to make any arm choice in 15 s were assigned one error. If the platform was not located within 60 s, the mice were gently guided through the water by placing a hand behind the animal to direct its swimming direction toward the platform. If the mouse located the platform within 60 s or was guided towards the platform, it was allowed to stay on the platform for 15 s. After that, the mouse was gently removed from the platform and thoroughly dried using cellulose paper before placing it back into its home cage under a heat source (a heat lamp).

For the statistical analysis of the data obtained, to minimize the impact of individual trial variability, each mouse error for the finding of the platform for three consecutive trials were averaged, giving five data points (5 trial blocks): T1 (trials 1–3), T2 (trials 4–6), T3 (trials 7–9), T4 (trials 10–12) and T5 (trials 13–15), separately for each day of testing.

***16.4. Influence on locomotor activity***

The locomotor activity test was performed using activity cages (40 × 40 × 30 cm; supplied with infrared beam emitters) (Activity Cage 7441, Ugo Basile, Italy), connected to a counter for the recording of light-beam interruptions. Sixty minutes before the experiment, the mice were intraperitoneally pretreated with 30 mg kg^–1^ test compound **2** or vehicle, then individually placed in the activity cages in a sound-attenuated room. The mouse locomotor activity (i.e., number of light-beam crossings) was measured during the next 30 min of the test36.

***16.5. Influence on motor coordination***

Before the test, the mice were trained for three consecutive days on the rotarod apparatus (Rotarod apparatus, May Commat RR0711, Turkey; rod diameter, 2 cm) that was rotating at a fixed speed of 18 rotations per minute (rpm). In each training session, the mice were put on the rotating rod for 3 min, with an unlimited number of trials. The correct experimentation was performed 24 h after the last training session. Sixty minutes after the administration of 30 mg kg^–1^ and 100 mg kg^–1^ compound **2** or the vehicle, the mice were tested on the rod that revolved at 6, 18 or 24 rpm. Motor impairments were defined as the inability to remain on the rotarod apparatus for 1 min, and were expressed as the mean time spent on the rotarod36.

**17. SUPPLEMENTARY REFERENCES**

1. Girisha, H. R. *et al.* Active site directed docking studies: synthesis and pharmacological evaluation of *cis*-2,6-dimethyl piperidine sulfonamides as inhibitors of acetycholinesterase. *Eur. J. Med. Chem.* **44,** 4057–4062 (2009).
2. Kang, J. E. *et al.* Inhbitory evaluation of sulfonamides chalcones on β-secretase and acycholinesterase. *Molecules* **18,** 140–153 (2013).
3. Khalid, H. et al. Synthesis, biological evaluation, and molecular docking of N’-(aryl/alkylsulfonyl)-1-(phenylsulfonyl) piperidine-4-carbohydrazide dervatives. *Turk. J. Chem.* **38,** 189–201 (2014).
4. Bag, S. Sulfonamides as multifunctional agents for Alzheimer’s disease. *Bioorg. Med. Chem. Lett.* **25,** 626–630 (2015).
5. Mutahir, S. *et al.* Novel biphenyl bis-suflonamides as acetyl and butyrylcholinesterase inhibitors: synthesis, biological evaluation and molecular modeling studies. *Bioorg. Med. Chem.* **64,** 13–20 (2016).
6. Abbasi, M. A. *et al.* Sulfonamide derivatives of 2-amino-1-phenylethane as suitable cholinesterase inhibitors. *Trop. J. Pharm. Res.* **13,** 739–745 (2014).
7. Khalid, H., Rehman, A., Abbasi, M. A. & Khan, K. M. Synthesis, spectral characterization and structure-activity relationship studies on some sulfonamides bearing piperidine nucleus. *Int. J. Pharm. Pharm. Sci.* **4,** 443–448 (2012).
8. Rehman, A. *et al.* Synthesis, characterization and biological screening of sulfonamides derived form 2-phenylethylamine. *Pak. J. Pharm. Sci.* **25,** 809–814 (2012).
9. Abbasi, M. A. *et al.* 2-Phenitidine derivatives as suitable inhibitors of butyrylcholinesterase. *Braz. J. Pharm. Sci.* **49,** 127–133 (2013).
10. Košak, U., Brus, B. & Gobec, S. Straightforward synthesis of orthogonally protected piperidin-3-ylmethanamine and piperidin-4-ylmethanamine derivatives. *Tetrahedron Lett.* **55,** 2037–2039 (2014).
11. Brus, B. *et al.* Discovery, biological bvaluation, and crystal structure of a novel nanomolar selective butyrylcholinesterase inhibitor. *J. Med. Chem.* **57,** 8167–8179 (2014).
12. Lawrence, H. R. *et al*. Synthesis and biological evaluation of naphthoquinone analogs as a novel class of proteasome inhibitors. *Bioorg. Med. Chem.* **18,** 5576–5592 (2010).
13. Abdel-Magid, A. F., Carson, K. G., Harris, B. D., Maryanoff, C. A. & Shah, R. D. Reductive amination of aldehydes and ketones with sodium triacetoxyborohydride: studies on direct and indirect reductive amination procedures. *J. Org. Chem.* **61,** 3849–3862 (1996).
14. Knez, D. *et al.* Structure-based development of nitroxoline derivatives as potential multifunctional anti-Alzheimer agents. *Bioorg. Med. Chem.* **23,** 4442–4452 (2015).
15. Brazzolotto, X. *et al.* Human butyrylcholinesterase produced in insect cells: huprine-based affinity purification and crystal structure. *FEBS J.* **279,** 2905–2916 (2012).
16. Kabsch, W. XDS. *Acta Crystallogr. D Biol. Crystallogr.* **66,** 125–132 (2010).
17. McCoy, A. J. *et al.* *Phaser* crystallographic software. *J. Appl. Crystallogr.* **40,** 658–674 (2007).
18. Adams, P. D. *et al.* *PHENIX*: a comprehensive Python-based system for macromolecular structure solution. *Acta Crystallogr. D Biol. Crystallogr.* **66,** 213–221 (2010).
19. Headd, J. J. *et al.* Use of knowledge-based restraints in *phenix.refine* to improve macromolecular refinement at low resolution. *Acta Crystallogr. D Biol. Crystallogr.* **68,** 381–390 (2012).
20. Emsley, P., Lohkamp, B., Scott, W. G. & Cowtan, K. Features and development of *Coot*. *Acta Crystallogr. D Biol. Crystallogr.* **66,** 486–501 (2010).
21. Schüttelkopf, A. W. & van Aalten, D. M. F. *PRODRG*: a tool for high-throughput crystallography of protein–ligand complexes. *Acta Crystallogr. D Biol. Crystallogr.* **60,** 1355–1363 (2004).
22. Koelle, G. B. & Friedenwald, J. A. A histochemical method for localizing cholinesterase activity. *Proc. Soc. Exp. Biol. Med. Soc. Exp. Biol. Med.* **70,** 617–622 (1949).
23. LeVine 3^rd^, H. Thioflavine T interaction with synthetic Alzheimer's disease beta-amyloid peptides: detection of amyloid aggregation in solution. *Protein Sci. Publ. Protein Soc.* 2, 404–410 (1993).
24. Žakelj, S., Berginc, K., Roškar, R., Kraljič, B. & Kristl, A. Do the recommended standards for *in vitro* biopharmaceutic classification of drug permeability meet the “passive transport” criterion for biowaives? *Curr. Drug Metab.* **14,** 21–27 (2013).
25. Banker, M. J., Clark, T. H. & Williams, J. A. Development and validation of a 96-well equilibrium dialysis apparatus for measuring plasma protein binding. *J. Pharm. Sci.* **92,** 967–974 (2003).
26. Di, L., Kerns, E. H., Hong, Y. & Chen, H. Development and application of high throughput plasma stability assay for drug discovery. *Int. J. Pharm.* **297,** 110–119 (2005).
27. Obach, R. S. *et al.* The prediction of human pharmacokinetic parameters from preclinical and in vitro metabolism data. *J. Pharmacol. Exp. Ther.* **283,** 46–58 (1997).
28. U.S. Department of Health and Human Services, Food and Drug Administration, Center for Drug Evaluation and Research (CDER), Center for Veterinary Medicine (CMV) Guidance for industry, bioanalytical method validation. 1–22, 2011.
29. Matuszewski, B.K., Constanzer M.L. & Chavez-Eng C.M. Strategies for the assessment of matrix effect in quantitative bioanalytical methods based on HPLC-MS/MS. *Anal Chem.* **75,** 3019–30 (2003).
30. Park, S. J. *et al.* The ameliorating effects of stigmasterol on scopolamine-induced memory impairments in mice. *Eur. J. Pharmacol.* **676,** 64–70 (2012).
31. Puzzo, D., Lee, L., Palmeri, A., Calabrese, G. & Arancio, O. Behavioral assays with mouse models of Alzheimer’s disease: practical considerations and guidelines. *Biochem. Pharmacol.* **88,** 450–467 (2014).
32. Patil, S. S., Sunyer, B., Höger, H. & Lubec, G. Evaluation of spatial memory of C57BL/6J and CD1 mice in the Barnes maze, the Multiple T-maze and in the Morris water maze. *Behav. Brain Res.* **198,** 58–68 (2009).
33. Bialuk, I., Dobosz, K., Potrzebowski, B. & Winnicka, M. M. CP55,940 attenuates spatial memory retrieval in mice. *Pharmacol. Rep. PR* **66,** 931–936 (2014).
34. Alamed, J., Wilcock, D. M., Diamond, D. M., Gordon, M. N. & Morgan, D. Two-day radial-arm water maze learning and memory task; robust resolution of amyloid-related memory deficits in transgenic mice. *Nat. Protoc.* **1,** 1671–1679 (2006).
35. Wilcock, D. M. *et al.* Passive immunotherapy against Abeta in aged APP-transgenic mice reverses cognitive deficits and depletes parenchymal amyloid deposits in spite of increased vascular amyloid and microhemorrhage. *J. Neuroinflammation* **1,** 24 (2004).
36. Sałat, K. *et al.* Anticonvulsant active inhibitor of GABA transporter subtype 1, tiagabine, with activity in mouse models of anxiety, pain and depression. *Pharmacol. Rep.* **67,** 465–472 (2015).
